# Exploring the microbial landscape of the nasopharynx in children: a systematic review of studies using next generation sequencing

**DOI:** 10.3389/frmbi.2023.1231271

**Published:** 2023-10-19

**Authors:** Petra Zimmermann

**Affiliations:** ^1^Department of Community Health, Faculty of Science and Medicine, University of Fribourg, Fribourg, Switzerland; ^2^Department of Paediatrics, Fribourg Hospital, Fribourg, Switzerland; ^3^Infectious Diseases Research Group, Murdoch Children’s Research Institute, Melbourne, VIC, Australia; ^4^Department of Paediatrics, The University of Melbourne, Melbourne, VIC, Australia

**Keywords:** 16S rRNA, shotgun analysis, nasal, respiratory tract infection, microbiome, asthma, bronchiolitis, otitis media

## Abstract

**Introduction:**

The nasopharynx harbours a diverse and dynamic microbial community, which plays an important role in maintaining the health and homeostasis of the respiratory tract, as well as in immune system development. Understanding factors that influence the composition of the nasopharyngeal microbiome in children and its association with diseases is of particular importance, as children are at a heightened risk for respiratory infections and other adverse health outcomes.

**Objectives:**

This review systematically summarises studies which investigated the nasopharyngeal microbiome in children, including its dynamics, stability over time, and the influence of intrinsic and extrinsic factors on its composition.

**Methods:**

MEDLINE was searched using the OVID interface. Original studies which investigated the nasopharyngeal microbiome using next generation sequencing in children were summarised.

**Results:**

The search identified 736 studies, of which 77 were included. The studies show that the nasopharyngeal microbiome in children is dynamic and influenced by many external factors. A high abundance of *Haemophilus*, *Moraxella*, and *Streptococcus* and a low abundance of *Corynebacterium* and *Dolosigranlum* are associated with adverse health outcomes such as respiratory tract infections, wheezing and asthma exacerbations. Factors which have been identified as risk factors for these adverse health outcomes, such as being born by Caesarean section, not being breast-fed, having siblings, day-care attendance, and antibiotic exposure have been shown to be associated with the aforementioned features in the nasopharyngeal microbiome.

**Conclusion:**

The association between specific nasopharyngeal microbial profiles and adverse health outcomes highlights the potential of the nasopharyngeal microbiome as a marker for identifying children at risk for disease and even more importantly, as an avenue for targeted interventions and preventive strategies.

## Introduction

The nasopharynx is the gateway to the respiratory tract and a site of continuous contact with the environment. It harbours a diverse and dynamic microbial community, collectively known as the nasopharyngeal microbiome, which plays an important role in maintaining the health and homeostasis of the respiratory tract (Cleary and Clarke, 2017). The colonising bacteria not only influence the development of the immune system but are also important for metabolism (Enoksson et al., 2020; Chun et al., 2021; Hou et al., 2022). Commensals and pathogens of the microbiome have agonistic and antagonistic interactions and disturbance of homeostasis can lead to overgrowth, diminished resilience to pathogen invasion and infections. From the nasopharynx pathogens can spread to cause acute otitis media (AOM), pneumonia or invade the bloodstream to cause sepsis and meningitis. Some children are more susceptible to infections, especially respiratory tract infections.

Recent advances in next-generation sequencing technologies have revolutionised our ability to comprehensively characterise the nasopharyngeal microbiome and its dynamics in health and disease. Understanding the nasopharyngeal microbiome in children is of particular importance, as children are at a heightened risk for respiratory infections and other diseases, such as recurrent wheezing or asthma.

While it is well known that the nasopharynx is rapidly colonised by *Streptococcus pneumoniae*, non-typeable *Haemophilus influenzae* and *Moraxella catarrhalis* in early infancy, less is known about the colonisation dynamics of commensal bacteria (van Meel et al., 2020). Various environmental factors, such as temperature, humidity, nutrient and oxygen availability, can influence the colonisation of the nasopharynx (Camarinha-Silva et al., 2012). In addition, the composition of the nasopharyngeal microbiome is also shaped by age, genetics, and microbial interactions (Enoksson et al., 2020; Reyman et al., 2021).

This systematic review summarises studies that have investigated the nasopharyngeal microbiome in children using next generation sequencing, including its dynamics, stability over time, and the influence of intrinsic and extrinsic factors on its composition. Studying the nasopharyngeal microbiome in children is crucial for advancing our knowledge of respiratory health and disease and for developing effective interventions to improve paediatric health outcomes.

## Methods

### Data sources

In May 2023, MEDLINE (1946 to present) was searched using the Ovid interface with the following search terms: ‘bacteria or microbiome or DNA or sequencing’ AND ‘nasopharynx’ AND ‘neonates or infant or children’ (see **Supplementary Data** for detailed search terms). No language or geographical limitations were used. References of retrieved articles were searched for additional publications.

### Study selection

Original studies which investigated the bacterial composition of the nasopharyngeal microbiome in neonates and (less than 28 days of age), infants (29 days to 12 months of age), and children and adolescents (1 to 18 years of age) with next-generation sequencing were included. Exclusion criteria were studies which: (i) investigated the microbiome of the anterior nares; (ii) investigated only certain bacteria and not the overall composition of the nasopharyngeal microbiome (iii) did not report results from children separately from these of adults; (iv) investigated children with cystic fibrosis; (v) investigated immunocompromised children; and (vi) investigated the effect probiotics on the nasopharyngeal microbiome.

### Data extraction

The following variables were extracted from the included studies: author, publication year, country, study type, number and characteristics of participants, age of participants, number of samples, timing of testing, collection method and storage conditions, analysis technique, important findings, strengths, limitations, and potential bias.

The studies were identified, selected, appraised, and synthesised following the Preferred Reporting Items for Systematic Reviews and Meta-Analyses (PRISMA) guidelines for systematic reviews, ensuring a comprehensive and rigorous approach to the synthesis of evidence (Page et al., 2021). Findings are presented as described in the original publications, as data to re-calculate the associations was not available in many publications.

### Primary aim

The aim of the study was to describe the composition of the nasopharyngeal microbiome in children, including its dynamics, stability over time, and the influence of intrinsic and extrinsic factors on its composition.

### Level of evidence

The level of evidence for each study was graded using the classification from the Oxford Centre for evidence-based medicine (Phillips et al., 2009).

## Results

**Figure 1:**
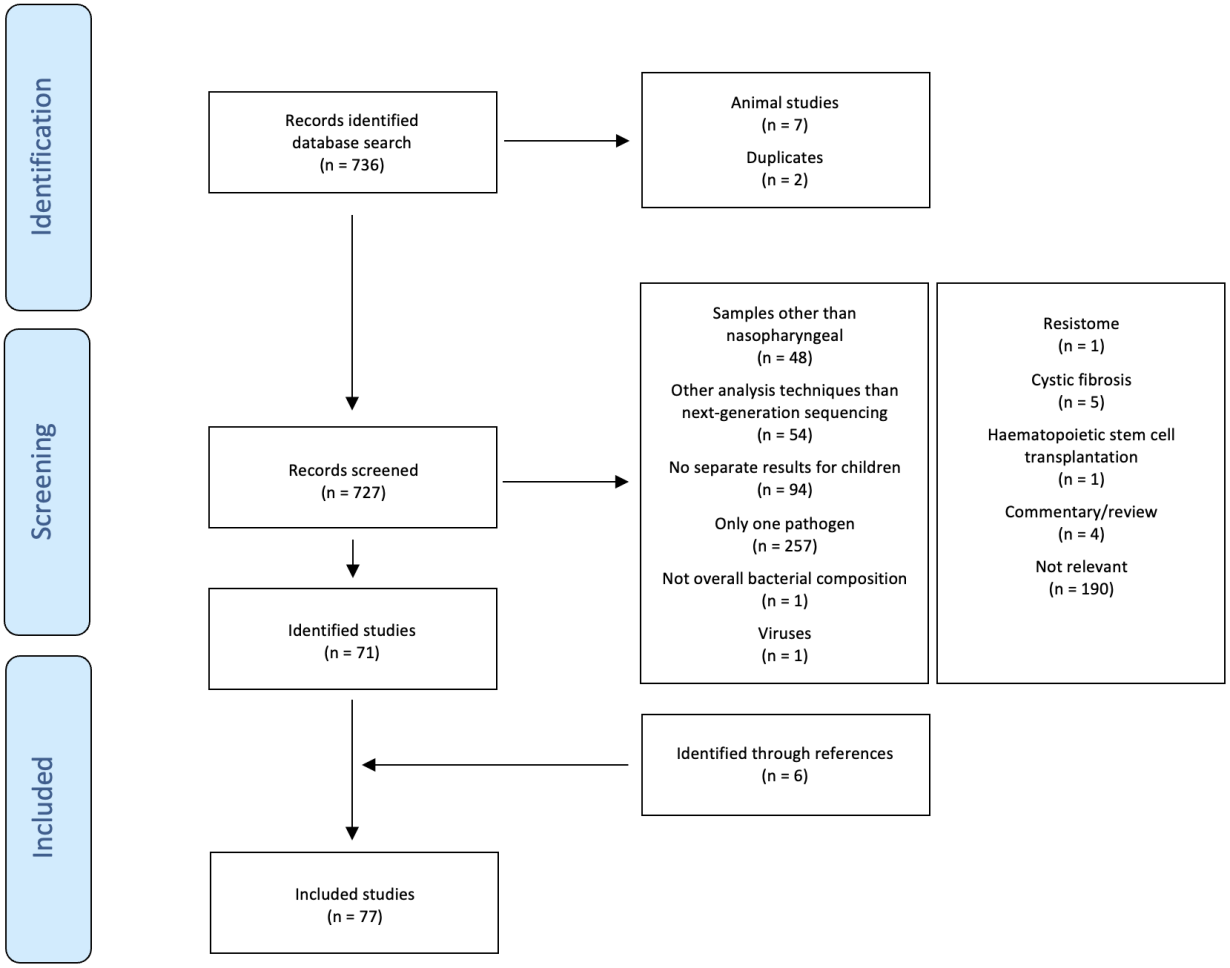
Selection of studies.

The search identified 736 studies. Of these, 71 fulfilled the inclusion criteria (Bogaert et al., 2011; Hilty et al., 2012; Biesbroek et al., 2014b; Biesbroek et al., 2014c; Sakwinska et al., 2014; Feazel et al., 2015; Jervis-Bardy et al., 2015; Stearns et al., 2015; Teo et al., 2015; Bosch et al., 2016; Hasegawa et al., 2016; Pérez-Losada et al., 2016; Rosas-Salazar et al., 2016a; Rosas-Salazar et al., 2016b; Shilts et al., 2016; Bosch et al., 2017; Chonmaitree et al., 2017; Hasegawa et al., 2017; Kelly et al., 2017; Pérez-Losada et al., 2017; Perez et al., 2017; Salter et al., 2017; Stewart et al., 2017; Dai et al., 2018; Ederveen et al., 2018; Kelly et al., 2018; Lappan et al., 2018; Luna et al., 2018; Pérez-Losada et al., 2018; Rosas-Salazar et al., 2018; Teo et al., 2018; Toivonen et al., 2018; Wen et al., 2018; Boelsen et al., 2019; Man et al., 2019c; Man et al., 2019b; Man et al., 2019a; Mansbach et al., 2019; McCauley et al., 2019; Stewart et al., 2019; Toivonen et al., 2019; Walker et al., 2019; Yau et al., 2019; Accorsi et al., 2020; Chapman et al., 2020; Enoksson et al., 2020; Haro et al., 2020; Liu et al., 2020; Mansbach et al., 2020; Salgado et al., 2020; Shilts et al., 2020; Thapa et al., 2020; Zhou et al., 2020; Aydin et al., 2021; Binia et al., 2021; Chun et al., 2021; Coleman et al., 2021; Elling et al., 2021; Folino et al., 2021; Fujiogi et al., 2021; Henares et al., 2021; McCauley et al., 2021; Reyman et al., 2021; Tang et al., 2021; Tozzi et al., 2021; Verhagen et al., 2021; Xu et al., 2021b; Hou et al., 2022; Kelly et al., 2022; McCauley et al., 2022; Tan et al., 2023). An additional six relevant studies were identified by hand-searching of references (Biesbroek et al., 2014a; Zhou et al., 2016; Lu et al., 2017; Man et al., 2020; Raita et al., 2021; Xu et al., 2021a). The selection of included studies is summarised in **Figure 1**. Of the 77 included studies, 36 reported findings from overlapping participants (Bogaert et al., 2011; Biesbroek et al., 2014b; Biesbroek et al., 2014c; Biesbroek et al., 2014a; Teo et al., 2015; Bosch et al., 2016; Hasegawa et al., 2016; Pérez-Losada et al., 2016; Rosas-Salazar et al., 2016a; Rosas-Salazar et al., 2016b; Bosch et al., 2017; Hasegawa et al., 2017; Kelly et al., 2017; Lu et al., 2017; Pérez-Losada et al., 2017; Stewart et al., 2017; Dai et al., 2018; Kelly et al., 2018; Luna et al., 2018; Pérez-Losada et al., 2018; Rosas-Salazar et al., 2018; Teo et al., 2018; Toivonen et al., 2018; Wen et al., 2018; Man et al., 2019a; Mansbach et al., 2019; Stewart et al., 2019; Toivonen et al., 2019; Mansbach et al., 2020; Zhou et al., 2020; Fujiogi et al., 2021; Raita et al., 2021; Reyman et al., 2021; Xu et al., 2021b; Xu et al., 2021a; Tan et al., 2023). Counting these studies only once, the following study designs were used: randomised placebo-controlled trial (2), prospective birth cohort study (6), prospective cohort study (17), retrospective cohort study (4), prospective case-control study (5), and cross-sectional study (17) (one study had two sub-studies with different study designs). The studies were done in Australia (4), Bangladesh (1), Botswana (2), Brazil (1), Canada (1), China (2), Fiji (1), Germany (1), Hong Kong (2), Israel (1), Italy (2), Japan (1), Kenya (1), the Netherlands (5), Venezuela (1), New Zealand (1), Spain (1), Switzerland (2), Sweden (1), Thailand (1), United Kingdom (1), and United States of Amerika (USA) (17). Excluding overlapping participants, the studies investigated 7,780 children (mean 151 children/study, range 11 to 1,005). The children included were healthy children (2,619), preterm-born children (487), children with an acute respiratory infection (ARTI) (200), an upper respiratory tract infection (URTI) (341), acute or chronic otitis media or middle ear effusion (777), a lower respiratory tract infection (LRTI) (1,987), allergic rhinoconjunctivitis (23), chronic wheezing (23), asthma (963), atopy, allergic rhinitis or recurrent wheezing (36), gastrointestinal infection (28), invasive pneumococcal disease (27), a chronic illness not further specified (11), and children prone to infection and allergy (492). Excluding overlapping samples, 20,571 samples (mean 375 samples/study, range 11 to 3,122) were analysed. Of these, 12,169 were nasopharyngeal swabs (30 taken with brushes), 2,115 nasopharyngeal washes, 3,332 nasopharyngeal aspirates, 2,922 swabs or aspirates, and 33 nasal filters. The analysis techniques used for the evaluation of the nasopharyngeal microbiome were shotgun metagenomic sequencing (2), 16S rRNA sequencing (50) (V1-V3 (8), V3 (3), V1-V4 (1), V3-V4 (13), V3-V5 (2), V4 (23), V5-V6 (1), V5-V7 (1), V4+ITS2 (1)), shotgun metagenomic plus 16S rRNA sequencing (V4) (3) and metatranscriptomics (1). The following platforms were used for sequencing: NovaSeq 6000 (1), NextSeq 500 (3), HiSeq (4), MiSeq (37), MiSeq plus NovaSeq 6000 (1), GS FLX Titanium (9), PacBio RS II (1), and 3130xl Genetic Analyzer (1). The following databases were used for taxonomic identification of 16S rRNA sequencing: SILVA (25), GreenGenes (9), Expanded Human Oral Microbiome (1), Human Oral Microbiome plus National Center for Biotechnology Information (NCBI) (1), Live Tree Project (1), MiSeq standard operating procedure plus SILVA (1), Ribosomal Database Project (RDP) (8), RDP plus SILVA (3), RDP, SILVA plus NCBI (1). The studies which used shotgun metagenomics for taxonomic identification used the NCBI RefSeq (1), MetaPhlAn2 (1), the GOTTCHA (1) or custom databases (2).

**Figure 2:**
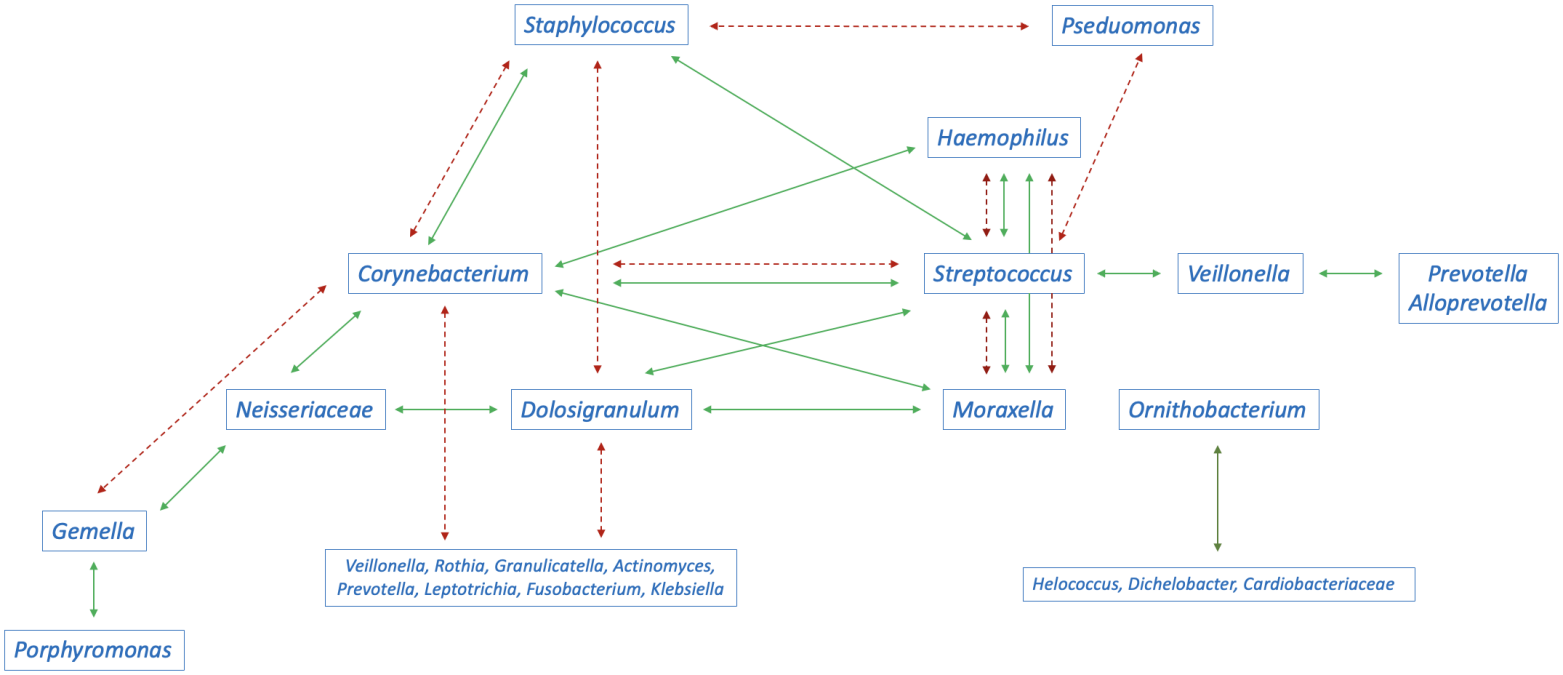
Interaction between the abundance of bacteria in the nasopharyngeal microbiome in children (green continuous arrows indicate positive correlations, red dashed arrows indicate negative correlations). Conflicting arrows result from studies reporting conflicting results.

**Figure 3:**
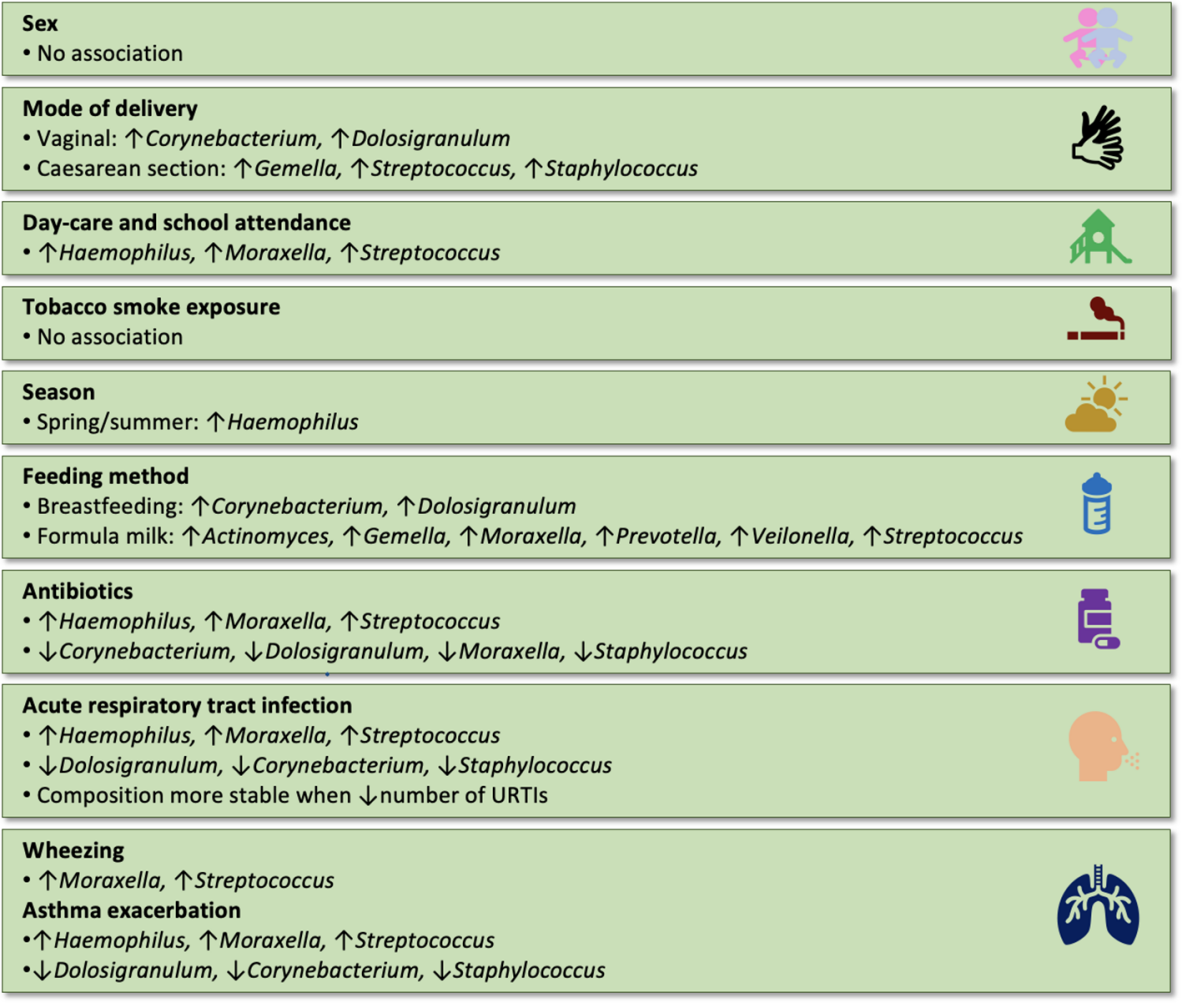
Summary of the associations between extrinsic factors and the composition of the nasopharyngeal microbiome in children (results were included when reported in at least two independent studies).

The main results of the studies are summarised in **Table 1**, **Figures 2**, **3** and **Supplementary Table 1**.

**Table 1:** Summary of findings of studies investigating the association between intrinsic and extrinsic factors and the composition of the nasopharyngeal microbiome in children using next generation sequencing.

HOST FACTORS
**Age** • ↓Diversity (Biesbroek et al., 2014a; Kelly et al., 2022) ↑richness (Kelly et al., 2022), ↑stability (Accorsi et al., 2020) during first 12m • ↑Diversity (Bosch et al., 2016) during first 3m • ↑Diversity with age, especially in children >24m (Teo et al., 2018) • Positive association between diversity at 1m and diversity up to 9m (Chonmaitree et al., 2017) • Composition more stable in infants compared with children 12 to 59m of age (Feazel et al., 2015) • At birth: most abundant genera *Staphylococcus, Dolosigranulum, Streptococcus* (Bosch et al., 2017; Man et al., 2019a), *S. viridans* (Bosch et al., 2016), *Gemella* (Bosch et al., 2016), ↑*Acinetobacter* (Kelly et al., 2022), ↑*Gardnerella* (Kelly et al., 2022), ↑*Lactobacillus* (Kelly et al., 2022), ↑*Sneathia* (Kelly et al., 2022) • First 1.5-3m: ↑*Staphylococcus* (Biesbroek et al., 2014b; Salter et al., 2017), ↑*Corynebacterium* (Biesbroek et al., 2014b; Salter et al., 2017), ↑*Dolosigranulum* (Biesbroek et al., 2014b), ↓*Haemophilus* (Biesbroek et al., 2014b) • First 6m: ↓*S. aureus* (Biesbroek et al., 2014b), ↑*H. influenzae* (Biesbroek et al., 2014b), ↑*S. pneumoniae* (Biesbroek et al., 2014b), ↑*M. catarrhalis* (Biesbroek et al., 2014b) • First 12m: increase in unclassified *Flavobacteriaceae (* Salter et al., 2017*)*, decrease in *Streptococcus (* Salter et al., 2017) • First 21m: ↑*Moraxella* (Salter et al., 2017) • Younger children higher abundance of *Moraxella, Haemophilus*, *Dolosigranulum* (McCauley et al., 2022) • Older children higher abundance of *Staphylococcus* and *Corynebacterium* (McCauley et al., 2022) • Two studies did not find an influence of age on diversity and composition during first 9 to 24m of life (Hilty et al., 2012; Chonmaitree et al., 2017), one on diversity and density in the first 24m of life (Biesbroek et al., 2014b)
Sex • ↑*Moraxella* in males during healthy periods but not during ARTI (Teo et al., 2015) • No association (Hilty et al., 2012; Feazel et al., 2015; Boelsen et al., 2019; Henares et al., 2021; Kelly et al., 2022)
**Ethnicity** • Non-Hispanic White children more frequently *Haemophilus*-dominated profile than children from other ethnicities (Hasegawa et al., 2016; Hasegawa et al., 2017; Toivonen et al., 2018; Toivonen et al., 2019; Fujiogi et al., 2021) • ↑Diversity in Fijian children of Indian descent than Fijian Indigenous children (Boelsen et al., 2019) • Fijian Indigenous children ↑*Moraxella*, ↑*Haemophilus*, ↑*Helococcus*, ↓*Staphylococcus*, ↑*Dolosigranulum*, ↑*Corynebacterium* than Fijian children of Indian descent (Boelsen et al., 2019) • Association without specifying further details (McCauley et al., 2021) • No association (Shilts et al., 2016; Chonmaitree et al., 2017)
PERINATAL FACTORS
**Delivery mode** Vaginally • Prolonged predominance of *Corynebacterium* (Bosch et al., 2017) and *Dolosigranulum* (Bosch et al., 2017) • Earlier switch to *Moraxella*- and *Corynebacterium/Dolosigranulum-*dominated profiles (Bosch et al., 2016) • Late enrichment of *Moraxella* (Bosch et al., 2017) • ↑*Corynebacterium* (Shilts et al., 2016), ↑*C. pseudodiphteriticum/propinquum (* Bosch et al., 2016*)*, ↑*D. pigrum (* Bosch et al., 2016) Caesarean section • ↑Richness (Shilts et al., 2016), ↑diversity (Shilts et al., 2016) • Prolonged predominance of *Actinomyces* (Bosch et al., 2017), *Granulicatella* (Bosch et al., 2017), *Neisseria* (Bosch et al., 2017), *Prevotella* (Bosch et al., 2017) • Longer persistence of *S. aureus*-dominated profile (Bosch et al., 2016) • ↑*Gemella* (Bosch et al., 2016; Bosch et al., 2017), ↑*Streptococcus* (Bosch et al., 2017) (*S. viridans (* Bosch et al., 2016*)*, *S. salivarius (* Bosch et al., 2016)), ↑*Staphylococcu*s (Shilts et al., 2016) (*S. aureus* (Bosch et al., 2016)) • Association without specifying details (Reyman et al., 2021) • No association (Teo et al., 2015; Bosch et al., 2016; Thapa et al., 2020; Chonmaitree et al., 2017; Zhou et al., 2020; Binia et al., 2021)
**Feeding method** Breast milk • ↑Evenness (Biesbroek et al., 2014b), ↑stability (Biesbroek et al., 2014b) • Early abundance of *Dolosigranulum* (Bosch et al., 2017), prolonged predominance of *Corynebacterium* (Bosch et al., 2017), *Dolosigranulum* (Bosch et al., 2017) • ↑*Corynebacterium/Dolosigranulum*-dominated profiles (Biesbroek et al., 2014b) • Late enrichment of *Moraxella* (Bosch et al., 2017) • ↑*Corynebacterium* (Biesbroek et al., 2014b; Kelly et al., 2022) (*C. pseudodiphteriticum* (Biesbroek et al., 2014b)*, C. propinquum* (Biesbroek et al., 2014b)*, C. accolens* (Biesbroek et al., 2014b)*, C. fastidiosum* (Biesbroek et al., 2014b)*, C. segmentosum* (Biesbroek et al., 2014b)), ↑*Dolosigranulum* (Biesbroek et al., 2014b) (*D. pigrum* (Biesbroek et al., 2014b)) (Biesbroek et al., 2014b) Formula milk • ↑Richness (Shilts et al., 2016) • Prolonged predominance of *Actinomyces* (Bosch et al., 2017)*, Granulicatella* (Bosch et al., 2017)*, Neisseria* (Bosch et al., 2017), *Prevotella* (Bosch et al., 2017) • ↑*Actinomyces* (Biesbroek et al., 2014b), ↑*Gemella* (Biesbroek et al., 2014b; Bosch et al., 2017), ↑*Granulicatella* (Biesbroek et al., 2014b), ↑*Haemophilus* (Kelly et al., 2022), ↑*Moraxella* (Shilts et al., 2016; Kelly et al., 2022), ↑*Porphyromonas* (Bosch et al., 2017), ↑*Prevotella* (Biesbroek et al., 2014b; Bosch et al., 2017), ↑*Rothia* (Biesbroek et al., 2014b), ↑*Staphylococcus* (Biesbroek et al., 2014b), ↑*Streptococcus* (Bosch et al., 2017; Kelly et al., 2022), ↑*Veillonella* (Biesbroek et al., 2014b; Bosch et al., 2017) • Association without specifying further details (Reyman et al., 2021) • No association (Teo et al., 2015; Thapa et al., 2020; Chonmaitree et al., 2017; Boelsen et al., 2019; Man et al., 2019c; Zhou et al., 2020; Binia et al., 2021; Henares et al., 2021)
ENVIRONMENTAL FACTORS
**Siblings and household size** Siblings • Accelerated microbiome maturation (Bosch et al., 2017) • ↑*Pasteurellaceae* (Bosch et al., 2017), ↑*Haemophilus* (Teo et al., 2015), ↑*Moraxella* (Teo et al., 2015), ↑*Streptococcus* (Teo et al., 2015), • ↓*Staphylococcus* (Teo et al., 2015) • Correlation between number of children in household and abundance of *S. pneumoniae* (Bogaert et al., 2011) • Association without specifying further details (Reyman et al., 2021) Household size • No association (Coleman et al., 2021)
**Day-care attendance** • Accelerated microbiome maturation (Bosch et al., 2017) • ↑*Moraxella* (Teo et al., 2015; Bosch et al., 2017) (↑*M. catarrhalis* (Accorsi et al., 2020)), ↑*Haemophilus* (Teo et al., 2015) (↑*H. influenzae* (Accorsi et al., 2020)), ↑*Streptococcus* (Teo et al., 2015) (*S. pneumoniae* (Xu et al., 2021b; Xu et al., 2021a)) • ↓*Staphylococcus* (Bosch et al., 2017) • Association without specifying further details (Reyman et al., 2021) • No assosciation (Bogaert et al., 2011; Hilty et al., 2012; Man et al., 2019c)
**Pets** • ↓*Streptococcus* (Teo et al., 2015) (furry pets) • Association without specifying further details (Reyman et al., 2021) • No association (Shilts et al., 2016)
**Tobacco smoke exposure** • No assosication (Bogaert et al., 2011; Boelsen et al., 2019)
**Season** • Spring: ↑*Moraxella* (McCauley et al., 2022), ↑*Bacillus* (Bogaert et al., 2011) (*B. fragilis*) (Bogaert et al., 2011), ↑*Brevibacillus* (Bogaert et al., 2011), ↑*Flavobacterium* (Bogaert et al., 2011), ↑*Lactobacillus* (Bogaert et al., 2011), ↑*Malassezia* (during ARTI) (McCauley et al., 2022), ↑*Haemophilus*-dominated profile (Teo et al., 2015; McCauley et al., 2021) • Summer: ↑*Haemophilus* (Pérez-Losada et al., 2017), ↑*Dolosigranulum/Corynebacterium*-dominated profile (McCauley et al., 2021), ↑*Haemophilus*-dominated profile (Teo et al., 2015) • Autumn: ↑*Staphylococcus* (McCauley et al., 2022), ↑*Dolosigranulum/Corynebacterium*-dominated profile (McCauley et al., 2021), ↑*Moraxella-dominated* profile (Teo et al., 2015), ↑*Candida* (during ARTI) (McCauley et al., 2022), ↑*Cladosporium* (during ARTI) (McCauley et al., 2022) • Winter: ↑*Haemophilus*-dominated profile (McCauley et al., 2021), ↑*Moraxella-dominated profile* (Teo et al., 2015) • Association without specifying further details (Reyman et al., 2021) • No association (Boelsen et al., 2019; Man et al., 2019c; Coleman et al., 2021)
HEALTH-CARE ASSOCIATED FACTORS
**Vaccination** • PCV: ↑diversity (Biesbroek et al., 2014c; Henares et al., 2021) • Hib/PCV10: ↑richness (Salgado et al., 2020) • PCV7/PCV13: ↓*S. pneumoniae* (Boelsen et al., 2019; Kelly et al., 2022) • PCV7: ↑*Haemophilus (* Biesbroek et al., 2014c*)*, ↑*Staphylococcus* (Biesbroek et al., 2014c), ↑*Veilonella* (Biesbroek et al., 2014c), ↑*Prevotella* (Biesbroek et al., 2014c), ↑*Bacteroidetes* (Biesbroek et al., 2014c), ↑*Leptorichia* (Biesbroek et al., 2014c), ↑*Streptococcus* (Biesbroek et al., 2014c) ↓*Streptococcaceae* (Hilty et al., 2012), ↓*Corynebacteriacea* (Hilty et al., 2012) • PCV7 (Hilty et al., 2012; Biesbroek et al., 2014b; Henares et al., 2021) or Hib/PCV10 (Feazel et al., 2015): no association with diversity or composition
**Antibiotics** • ↑Diversity (Chonmaitree et al., 2017) • ↑*Bifidobacterium* (Chonmaitree et al., 2017), ↑*Brachybacterium* (Salter et al., 2017), ↑*Dolosigranulum* (Salter et al., 2017), ↑*Firmicutes incertae sedis* (Salter et al., 2017), ↑*Haemophilus* (Teo et al., 2015; Hasegawa et al., 2016; Thapa et al., 2020; Hasegawa et al., 2017; Luna et al., 2018; Toivonen et al., 2018; Toivonen et al., 2019; Fujiogi et al., 2021; Raita et al., 2021; Kelly et al., 2022), ↑*Moraxella* (Teo et al., 2015; Kelly et al., 2022), ↑*Pasteurellaceae* (Hilty et al., 2012), ↑*Streptococcus* (Teo et al., 2015; Salter et al., 2017; Kelly et al., 2022), • ↓*Dolosigranulum* (Teo et al., 2015; Bosch et al., 2017; Chonmaitree et al., 2017), ↓*Corynebacterium* (Teo et al., 2015; Bosch et al., 2017; Chonmaitree et al., 2017; Kelly et al., 2022), ↓*Enterobacter* (Chonmaitree et al., 2017), ↓*Lactobacillus* (Kelly et al., 2022), ↓*Moraxellaceae* (Hilty et al., 2012) (↓*Moraxella* (Zhou et al., 2016; Salter et al., 2017)), ↓*Staphylococcacea* (Hilty et al., 2012) (↓*Staphylococcus* (Chonmaitree et al., 2017)), ↓*Streptococcaceae* (Hilty et al., 2012) • Association without specifying further details (Reyman et al., 2021) • No association (Bogaert et al., 2011; Feazel et al., 2015; Boelsen et al., 2019; Man et al., 2019c)
DISEASE-ASSOCIATED FACTORS
**ARTI** During ARTI • *Haemophilus-* (Teo et al., 2015; Teo et al., 2018) (*H. influenzae-* (Tang et al., 2021))*, Moraxella-* (Teo et al., 2015; Teo et al., 2018) (*M. catarrhalis-* (Tang et al., 2021)*), Streptococcus-* (Teo et al., 2015; Teo et al., 2018) (*S. pneumoniae-* (Tang et al., 2021))dominated profiles more frequent • *Dolosigranulum-* (Teo et al., 2015) (*D. pigrum-* (Tang et al., 2021)*), Corynebacterium-* (Teo et al., 2015) (*C. pseudodipththeriticum-* (Tang et al., 2021)), *Staphylococcus-* (Teo et al., 2015; Tang et al., 2021), *S. mitis-* (Tang et al., 2021) dominated profiles less frequent • Most abundant genera *Moraxella* (Rosas-Salazar et al., 2016a; Salgado et al., 2020), *Streptococcus* (Rosas-Salazar et al., 2016a; Salgado et al., 2020), *Corynebacterium* (Rosas-Salazar et al., 2016a), *Haemophilus* (Rosas-Salazar et al., 2016a; Salgado et al., 2020), *Dolosigranulum* (Rosas-Salazar et al., 2016a) • ↑*Fusobacterium* (Man et al., 2019a), ↑*J. lividum* (Man et al., 2019a), ↑*Neisseria* (Teo et al., 2015) (*N. lactamica* (Man et al., 2019a)), ↑*P. nanceiensis (* Man et al., 2019a) ↑*Streptococcus* (Man et al., 2019a) • ↓*Corynebacterium* (Teo et al., 2018; Man et al., 2019a; Verhagen et al., 2021), ↓*Dolosigranulum* (Teo et al., 2018; Man et al., 2019a), ↓*Staphylococcus* (Teo et al., 2018) • Positive association between abundance of *Haemophilius* (Teo et al., 2018), *Moraxella* (Teo et al., 2018), *Streptococcus* (Teo et al., 2018) and severity of ARTI Risk for ARTI • Frequent ARTI associated with ↓stability (Bosch et al., 2017),*H. influenzae*-dominated profile (Bosch et al., 2016) • Frequent ARTI associated ↑*Moraxella* (Teo et al., 2015; Bosch et al., 2017), ↑*Haemophilus* (Bosch et al., 2017), ↑*Neisseria* (Bosch et al., 2017), ↑*Prevotella* (Bosch et al., 2017),↑*Alloprevotella* (Bosch et al., 2017) • Frequent ARTI associated with ↓*Streptococcus* (Bosch et al., 2017),↓*Corynebacterium* (Teo et al., 2015; Bosch et al., 2017), ↓*Dolosigranulum* (Teo et al., 2015; Bosch et al., 2017) • ↑*S. pneumoniae* (Kelly et al., 2018) associated with ↑risk for ARTIs • ↑*S. gordonni* (Teo et al., 2018), ↑*S. thermophilus/salivarius/vestibularis* (Teo et al., 2018) associated with ↓risk for ARTIs • ↑*Moraxella* before ARTI (Teo et al., 2018) • No assosciation (Binia et al., 2021) RSV infection • ↓Diversity (Rosas-Salazar et al., 2016a), ↓richness (Rosas-Salazar et al., 2016a; Rosas-Salazar et al., 2016b) • Most abundant genera were *Moraxella* (Rosas-Salazar et al., 2016b; Rosas-Salazar et al., 2018; Tan et al., 2023), *Streptococcus* (Rosas-Salazar et al., 2016b; Rosas-Salazar et al., 2018; Tan et al., 2023)*, Staphylococcus* (Tan et al., 2023)*, Haemophilus* (Rosas-Salazar et al., 2016b; Rosas-Salazar et al., 2018; Tan et al., 2023)*, Corynebacterium* (Rosas-Salazar et al., 2016b; Rosas-Salazar et al., 2018; Tan et al., 2023), *Dolosigranulum* (Rosas-Salazar et al., 2018; Tan et al., 2023) • Association with *S. pneumoniae-*dominated profile (Toivonen et al., 2019; Raita et al., 2021) • *Haemophilus*-dominated profile associated with delayed RSV clearance (Mansbach et al., 2019) • ↑*Achromobacter* (Ederveen et al., 2018), ↑*Haemophilus* (Rosas-Salazar et al., 2016b; Ederveen et al., 2018), ↑*Moraxella* (Rosas-Salazar et al., 2016b), ↑*Streptococcus* (Rosas-Salazar et al., 2016b) • ↓*Staphylococcus* (Rosas-Salazar et al., 2016b), ↓*Corynebacterium* (Rosas-Salazar et al., 2016b) ↓*Veillonella* (Ederveen et al., 2018) • Colonisation with *Dolosigranulum* associated with had fewer RSV infections, especially RSV LRTIs (Teo et al., 2015) • Positive correlation between abundance of *Haemophilus* and CXCL8 levels (indicative for higher disease severity) (Ederveen et al., 2018) Rhinovirus infection • Most abundant genera *Streptococcus* (Perez et al., 2017)*, Moraxella*, ^ (Perez et al., 2017) *Staphylococcus* (Perez et al., 2017), *Burkholderia* (Perez et al., 2017)*, Neisseria* (Perez et al., 2017), *Haemophilus* (Perez et al., 2017), *Janthinobacterium* (Perez et al., 2017) • ↑*Moraxella* (Tozzi et al., 2021) • ↓*Streptococcus* (Toivonen et al., 2019) • *Haemophilus*-dominant profile associated with rhinovirus A infection (Toivonen et al., 2019; Raita et al., 2021) • *Moraxella*-dominant profile associated with rhinovirus C infection (Toivonen et al., 2019; Raita et al., 2021) Influenza infection • ↑Diversity (Wen et al., 2018; Zhou et al., 2020) • 5 profiles: *Moraxella- (* Zhou et al., 2020*)*, *Streptococcus-* (Zhou et al., 2020), *Staphylococcus-* (Zhou et al., 2020), *Corynebacterium-* (Zhou et al., 2020*), Dolosigranulum-*dominant profiles (Zhou et al., 2020) • ↓*Corynebacterium (* Wen et al., 2018; Zhou et al., 2020*)*, ↓*Dolosigranulum (* Wen et al., 2018; Zhou et al., 2020*)*, ↓*Moraxella (* Wen et al., 2018; Zhou et al., 2020*)*, ↓*Staphylococcus* (Wen et al., 2018; Zhou et al., 2020) • ↑*Acinetobacter (* Wen et al., 2018; Zhou et al., 2020*)*, ↑*Acidobacteria (* Wen et al., 2018; Zhou et al., 2020*)*, ↑*Halomonas (* Wen et al., 2018; Zhou et al., 2020*)*, ↑*Lachnoclstiridum (* Wen et al., 2018; Zhou et al., 2020*)*, ↑*Phyllobacterium* (Wen et al., 2018; Zhou et al., 2020), ↑*Pseudomonas (* Wen et al., 2018; Zhou et al., 2020*)*, ↑*Ralstonia* (Wen et al., 2018; Zhou et al., 2020) Mycoplasma infection • ↓Diversity (Zhou et al., 2020) • More often a *Staphylococcus*-dominated profile (Zhou et al., 2020) • ↑*Acidobacteria (* Zhou et al., 2020*)*, ↑*Ralastonia* (Zhou et al., 2020) Pertussis infection • ↑*Alcaligenaceae (* Tozzi et al., 2021*)*, ↑*Achromobacter* (Tozzi et al., 2021)
**URTI** During URTI • ↑*Moraxella-* (Kelly et al., 2017), ↑*Streptococcus-* (Kelly et al., 2017)domianted profiles • ↑*Haemophilus* (Chonmaitree et al., 2017; Kelly et al., 2017; Boelsen et al., 2019), ↑*Moraxella* (Chonmaitree et al., 2017; Kelly et al., 2017; Boelsen et al., 2019), ↑*Streptococcus* (Chonmaitree et al., 2017; Kelly et al., 2017; Boelsen et al., 2019) • ↓*Corynebacterium (* Boelsen et al., 2019*)*, ↓*Dolosigranulum* (Boelsen et al., 2019), ↓*Myroides* (Chonmaitree et al., 2017), ↓*Neisseriaceae* (Coleman et al., 2021), ↓*Pseudomonas* (Chonmaitree et al., 2017), ↓*Staphylococcus* (Kelly et al., 2017; Coleman et al., 2021) (↓*S.aureus* (Coleman et al., 2021)), ↓*Sphingomonas* (Chonmaitree et al., 2017), ↓*Yersinia* (Chonmaitree et al., 2017) • ↑*Haemophilus and* ↑*Streptococcus* associated with presence of virus (Chonmaitree et al., 2017) Risk for URTI • Frequent ARTI associated with ↓stability (Biesbroek et al., 2014b) • Early colonisation with *Moraxella* associated with earlier occurrence of URTI (Teo et al., 2015) • *Haemophilus*- (McCauley et al., 2021), *Moraxella-* (McCauley et al., 2021)dominated profiles more frequent URTIs • *Corynebacterium/Dolosigranulum*- (Biesbroek et al., 2014b; McCauley et al., 2021), *Moraxella-* (Biesbroek et al., 2014b)dominated profile less frequent URTIs • ↑*Gemella* (Biesbroek et al., 2014a), ↑*Moraxella* (McCauley et al., 2021) • ↓*Acetobacteraceae* (McCauley et al., 2021), ↓*Chryseobacterium* (McCauley et al., 2021), ↓*Dolosigranulum* (McCauley et al., 2021), ↓*Prevotella* (McCauley et al., 2021)
**AOM** During AOM • ↓Diversity (Hilty et al., 2012), ↓richness (Hilty et al., 2012), ↑density (Hilty et al., 2012) No difference in diversity (Chonmaitree et al., 2017) • ↑*Moraxella* (Chonmaitree et al., 2017), ↑*Haemophilus* (Chonmaitree et al., 2017), ↑*Streptococcus* (Chonmaitree et al., 2017) • ↓*Acidaminococcaceae* (Hilty et al., 2012*)*, ↓*Comamonadaceae (* Hilty et al., 2012*)*, ↓*Corynebacteriaceae (* Hilty et al., 2012*)*, ↓*Staphylococcacea* (Hilty et al., 2012) • Abundance of *Acinetobacter, Klebsiella, Neisseria, Haemophilus* associated with longer duration of otorrhoea (Man et al., 2019b) • Abundance of *Corynebacterium, Dolosigranulum, Haemophilus* associated with shorter duration of otorrhoea (Man et al., 2019b) Risk for AOM • ↑*Staphylococcus*, ↑*Sphingobium* associated with ↓risk of developing AOM after URTI (Chonmaitree et al., 2017) • Infants who develop AOM ↑*Bifidobacterium* (Chonmaitree et al., 2017), ↑*Enterobacter* (Chonmaitree et al., 2017), ↑*Haemophilus* (Chonmaitree et al., 2017), ↑*Yersinia* (Chonmaitree et al., 2017), ↓*Corynebacterium* (Chonmaitree et al., 2017), ↓*Myroides* (Chonmaitree et al., 2017), ↓*Pseudomonas* (Chonmaitree et al., 2017) Recurrent AOM • ↓Diversity at 6 but not 12m of age (Xu et al., 2021b; Xu et al., 2021a) • ↑Diversity (Lappan et al., 2018) • Most abundant genera *Moraxella* (Folino et al., 2021)*, Streptococcus* (Folino et al., 2021)*, Haemophilus* (Folino et al., 2021), *Dolosigranulum* (Folino et al., 2021)*, Corynebacterium* (Folino et al., 2021) • ↑*Alloprevotella* (Lappan et al., 2018), ↑*Dolosigranulum* (Xu et al., 2021b; Xu et al., 2021a), ↑*Fusobacterium (* Lappan et al., 2018*)*, ↑*Gemella (* Lappan et al., 2018*)*, ↑*Moraxella* (Coleman et al., 2021; Xu et al., 2021b; Xu et al., 2021a), ↑*Neisseria* (Lappan et al., 2018), ↑*Porphyromonas (* Lappan et al., 2018*)*, • ↓*Bacillus* (Xu et al., 2021b; Xu et al., 2021a), ↓*Corynebacterium* (Lappan et al., 2018), ↓*Dolosigranulum* (Lappan et al., 2018), ↓*Gemella* (Xu et al., 2021b; Xu et al., 2021a), ↓*Fusobacterium* (Xu et al., 2021b; Xu et al., 2021a), ↓*Micrococcus* (Chonmaitree et al., 2017), ↓*Prevotella* (Xu et al., 2021b; Xu et al., 2021a) ↓*Veillonella* (Xu et al., 2021b; Xu et al., 2021a) • Carrier of a fucosyltransferase 2 variant (associated with ↑risk for AOM) ↑*Cutibacterium*, ↓*Actinobacillus*, ↓*Selenomonas*, ↓*Saccharibacteria* (Elling et al., 2021) • Carrier of Ras interacting protein 1 variant (associated with ↑risk for AOM) ↑*Cutibacterium*, ↑*Escherichia-Shigella*, ↑*Staphylococcus*, ↓*Acintobacillus* (Elling et al., 2021) • No difference in diversity (Coleman et al., 2021), no association with composition (Hilty et al., 2012) **Otitis media with effusion** • Most abundant genera *Corynebacterium* (Enoksson et al., 2020)*, Dolosigranulum* (Enoksson et al., 2020)*, Haemophilus* (Enoksson et al., 2020), *Moraxella* (Enoksson et al., 2020), *Ornithobacterium* (Jervis-Bardy et al., 2015), *Streptococcus* (Jervis-Bardy et al., 2015; Enoksson et al., 2020) • Most abundant taxa *C. pseudodiphtheriticum* (Jervis-Bardy et al., 2015)*, D. pigrum* (Jervis-Bardy et al., 2015)*, M. catarrhalis* (Jervis-Bardy et al., 2015)*, H. influenzae* (Jervis-Bardy et al., 2015), • ↑*Ornithobacterium* (Coleman et al., 2021) • *Haemophilus, C. propinquum* associated with anti-inflammatory mediators (Enoksson et al., 2020) • *Turicella, Dolosigranulum* associated with pro-inflammatory mediators (Enoksson et al., 2020) **Chronic otitis media** • ↓Diversity (Walker et al., 2019) • ↑*Corynebacterium-* (Walker et al., 2019), ↑*Moraxella*- (Walker et al., 2019), ↑*Streptococcus*- (Walker et al., 2019) dominated profiles • ↑*H. influenzae* (Walker et al., 2019), ↑*M. catarrhalis* (Walker et al., 2019), ↑*M. caprae* (Walker et al., 2019), ↑*S. pneumoniae* (Walker et al., 2019), • ↓*C. acnes* (Walker et al., 2019), ↓*Capnocytophaga* (Walker et al., 2019), ↓*Lactococcus* (Walker et al., 2019), ↓*Lautropia* (Walker et al., 2019), ↓*Neisseria* (Walker et al., 2019), ↓*Oxalobacteraceae* (Walker et al., 2019), ↓*S. infantis* (Walker et al., 2019)
**LRTI** • Most abundant species *M. catarrhalis/nonliquefaciens* (Man et al., 2019c)*, H. influenzae/haemolyticus* (Man et al., 2019c)*, S. pneumoniae* (Man et al., 2019c) • ↑*H. influenzae/haemolyticus-*, ↑*S. pneumoniae-*dominated profiles (Man et al., 2019c) • ↓*M. catarrhalis/nonliquefaciens-* ↓*C. propinquum/D. pigrum-*dominated profiles (Man et al., 2019c) • ↑*H. influenzae/haemolyticus* (Man et al., 2019c), ↑*S. pneumoniae* (Man et al., 2019c), ↑*Actinomyces (* Man et al., 2019c*)*, ↑*Prevotella* (Man et al., 2019c) • ↓*Moraxella* (Man et al., 2019c), ↓*C. propinqsuum* (Man et al., 2019c), ↓*D. pigrum* (Man et al., 2019c), ↓*Helococcus* (Man et al., 2019c) • Early colonisation with *Streptococcus* associated with earlier LRTI (Teo et al., 2015) • Acquisition of a new S*. pneumoniae* serotype not associated with LRTI (Salter et al., 2017) Bronchiolitis • Most abundant genera *Streptococcus* (Hasegawa et al., 2016; Hasegawa et al., 2017; Luna et al., 2018; Toivonen et al., 2018; Stewart et al., 2019; Toivonen et al., 2019; Fujiogi et al., 2021), *Moraxella* (Hasegawa et al., 2016; Hasegawa et al., 2017; Luna et al., 2018; Toivonen et al., 2018; Stewart et al., 2019; Toivonen et al., 2019; Fujiogi et al., 2021), *Haemophilus* (Hasegawa et al., 2016; Hasegawa et al., 2017; Luna et al., 2018; Toivonen et al., 2018; Stewart et al., 2019; Toivonen et al., 2019; Fujiogi et al., 2021), *Prevotella* (Hasegawa et al., 2016; Hasegawa et al., 2017; Toivonen et al., 2018; Toivonen et al., 2019; Fujiogi et al., 2021), *Staphylococcus (* Hasegawa et al., 2016; Hasegawa et al., 2017; Toivonen et al., 2018; Toivonen et al., 2019; Fujiogi et al., 2021) • *Haemophilus-dominant* profile associated with ↑severity and ↑length of hospital stay • *Moraxella-dominant* microbiome was associated with less frequent admission to an intensive care unit (Hasegawa et al., 2016; Hasegawa et al., 2017; Luna et al., 2018; Toivonen et al., 2018; Toivonen et al., 2019; Fujiogi et al., 2021) Pneumonia • ↓Diversity (Dai et al., 2018 ,Sakwinska et al., 2014 ,Lu et al., 2017), ↓richness (Sakwinska et al., 2014) • ↑*Haemophilus-* (Kelly et al., 2017*)*, ↑*Staphylococcus-* (Kelly et al., 2017), ↑*Streptococcus*- (Kelly et al., 2017) dominant microbiome profiles • ↑Firmicutes (Lu et al., 2017; Dai et al., 2018), ↑*Acinetobacter* (Lu et al., 2017; Dai et al., 2018), ↑*Actinomyces* (Lu et al., 2017; Dai et al., 2018),↑*Escherichia* (Kelly et al., 2017), ↑*Haemophilus* (Sakwinska et al., 2014; Kelly et al., 2017), ↑*Klebsiella* (Kelly et al., 2017), ↑*Lactobacillus* (Lu et al., 2017; Dai et al., 2018), ↑*Moraxella* (Sakwinska et al., 2014), ↑*Mycoplasma* (Lu et al., 2017; Dai et al., 2018), ↑*Ralstonia* (Lu et al., 2017; Dai et al., 2018), ↑*Staphylococcus* (Lu et al., 2017; Dai et al., 2018), ↑*Streptococcus* (Sakwinska et al., 2014; Kelly et al., 2017; Lu et al., 2017; Dai et al., 2018) • ↓Bacteroidetes (Lu et al., 2017; Dai et al., 2018), ↓*Corynebacterium* (Kelly et al., 2017)*, Dolosigranulum* (Kelly et al., 2017; Lu et al., 2017; Dai et al., 2018),↓*Moraxella* (Lu et al., 2017; Dai et al., 2018), ↓*Prevotella* (Lu et al., 2017; Dai et al., 2018)
**Atopy** • ↑*Burkholderiaceae* (Aydin et al., 2021), ↑*Enterobacteriaceae* (Aydin et al., 2021), ↑*Sphingomonadaeae* (Aydin et al., 2021), ↑*Staphylococcaceae* (Aydin et al., 2021), ↑*Xanthobacteraceae* (Aydin et al., 2021) • Cat allergy: ↓diversity (Chun et al., 2021), ↓*Corynebacterium* (Chun et al., 2021), ↓*S. epidermidis* (Chun et al., 2021) • Dog allergy: ↓*Corynebacterium* (Chun et al., 2021) • Pollen allergy: no association (Chun et al., 2021)
**Wheezing** • Acute wheezing: ↑*S. pneumoniae*-dominated profile (Tang et al., 2021), ↓*D. pigrum-*dominated profile (Tang et al., 2021) • Chronic wheezing: ↑*Burkholderiaceae* (Aydin et al., 2021), ↑*Enterobacteriaceae* (Aydin et al., 2021), ↑*Sphingomonadaeae* (Aydin et al., 2021), ↑*Staphylococcaceae* (Aydin et al., 2021) (↑*S. aureus* (Aydin et al., 2021)), ↑*Xanthobacteraceae* (Aydin et al., 2021), ↑*Haemophilus* (Aydin et al., 2021) (*H. influenzae* (Aydin et al., 2021)), ↑*Moraxella* (Zhou et al., 2016; Mansbach et al., 2020; Aydin et al., 2021) (*M. catarrhalis* (Aydin et al., 2021)*), Streptococcus* (Teo et al., 2015; Mansbach et al., 2020) ↑*S. pneumoniae* (Aydin et al., 2021) • ↓*Lactobacillus* (Rosas-Salazar et al., 2018), ↓*Staphylococcus* (Rosas-Salazar et al., 2018)
**Asthma** • ↑Firmicutes, ↑*Staphylococcaceae* than healthy children (Aydin et al., 2021) • Most abundant genera: *Moraxella* (Pérez-Losada et al., 2017; Pérez-Losada et al., 2018), *Staphylococcus* (Pérez-Losada et al., 2017; Pérez-Losada et al., 2018), *Dolosigranulum* (Pérez-Losada et al., 2017; Pérez-Losada et al., 2018), *Corynebacterium* (Pérez-Losada et al., 2017; Pérez-Losada et al., 2018), *Prevotella* (Pérez-Losada et al., 2017; Pérez-Losada et al., 2018), *Streptococcus* (Pérez-Losada et al., 2017; Pérez-Losada et al., 2018), *Haemophilus* (Pérez-Losada et al., 2017; Pérez-Losada et al., 2018), *Fusobacterium* (Pérez-Losada et al., 2017; Pérez-Losada et al., 2018) • *Moraxella*-dominated profiles (McCauley et al., 2019), ↑*Haemophilus* (McCauley et al., 2022), ↑*Moraxella* (Hou et al., 2022; McCauley et al., 2022) associated with ↑exacerbation risk • *Staphylococcus*- (McCauley et al., 2019), Dolosigranulum- (McCauley et al., 2019), *Corynebacterium*- (McCauley et al., 2019; Hou et al., 2022) dominated profiles ↓exacerbation risk • Association between microbiome composition of the nasopharynx and the asthmatic phenotype (McCauley et al., 2019) • ↑*H. influenzae* (Raita et al., 2021), ↑*S. pneumoniae (* Raita et al., 2021) during hospitalisation with severe bronchiolitis in infancy associated with ↑risk of asthma at 5y • ↑*Haemophilus* (Man et al., 2020), ↑*S. pneumoniae* (Man et al., 2020) associated with ↑reversible airway obstruction • ↓*Moraxella* (Man et al., 2020), ↓*Corynebacterium* (Man et al., 2020), ↓*Dolosigranulum* (Man et al., 2020), ↓*Staphylococcus* (Man et al., 2020) associated with ↑reversible airway obstruction

AOM, acute otitis media; m, month; ARTI, acute respiratory tract infection; PCV, pneumococcus conjugated vaccine; CXCL8, C-X-C motif chemokine ligand 8; RSV, respiratory syncytial virus; Hib, Haemophilus influenzae type b; URTI, upper respiratory tract infection; LRTI, lower respiratory tract infection; y, years.

### Host factors

#### Age

The composition of microbial communities in the nasopharynx in different age groups of children has been investigated in numerous studies, revealing varying levels of diversity and taxonomic identification. In healthy infants up to six months of age, 13 bacterial phyla (Biesbroek et al., 2014a), 29 to 328 genera (Shilts et al., 2016; Chapman et al., 2020), 60 species (Shilts et al., 2020), and 895 to 1,354 operational taxonomic units (OTUs) (Biesbroek et al., 2014a; Bosch et al., 2016; Reyman et al., 2021) were identified. In studies including healthy children up to 24 months of age, ten phyla (Biesbroek et al., 2014b; Biesbroek et al., 2014c), and 297 to 314 OTUs (Biesbroek et al., 2014b; Biesbroek et al., 2014c; Salter et al., 2017) were identified and in studies in toddlers up to 73 genera (Feazel et al., 2015; Coleman et al., 2021) and 121 OTUs (Verhagen et al., 2021). Studies including both healthy children and children with ARTI identified up to 15 phyla (Bosch et al., 2017; Tozzi et al., 2021), 95 families (Tozzi et al., 2021), 21 genera (Chonmaitree et al., 2017), and 245 (Tozzi et al., 2021) to 13,982 OTUs (Bosch et al., 2016; Bosch et al., 2017; Chonmaitree et al., 2017; Boelsen et al., 2019; McCauley et al., 2021). Studies including children with asthma identified 30 phyla (Hou et al., 2022), 396 to 436 genera (Pérez-Losada et al., 2017; Hou et al., 2022), and 21 to 8,034 OTUs (Shilts et al., 2016; Pérez-Losada et al., 2017; Pérez-Losada et al., 2018).

Several prospective birth cohort studies have investigated the dynamics of the nasopharyngeal microbiome during early life, revealing diverse and sometimes contrasting findings regarding the relationship between age and microbial diversity, richness, and density. Two prospective birth cohort studies found a higher diversity of the nasopharyngeal microbiome at birth compared with later periods during the first year of life (Biesbroek et al., 2014a; Kelly et al., 2022). The first study also found a positive association between increasing age and an increase in bacterial richness (Kelly et al., 2022). In contrast to these findings, a decrease in richness between the age of two to four months, without any difference in diversity has been reported in another study (Binia et al., 2021). An increase in the stability of species composition with increasing age has been described during the first year of life (Accorsi et al., 2020). Another prospective birth cohort study reported an increase in diversity with age, especially in children older than two years of age (Teo et al., 2018), while another study reported a positive individual correlation between diversity at one month of age and during the following eight months, but no overall association between age and diversity (Chonmaitree et al., 2017). Yet another prospective birth cohort study reported an increase in diversity during the first three months of life with the most changes in the microbiome composition during the first two months of life (Bosch et al., 2016), while another study reported a decrease in diversity and evenness during the first six months of life (Biesbroek et al., 2014a). One study found no differences in diversity or density during the first two years of life (Biesbroek et al., 2014b). An increase in bacterial density during the first month of life has been reported in a prospective birth cohort study (Bosch et al., 2017; Man et al., 2019a), while a retrospective cohort study found no change in density during the first two years of life (Biesbroek et al., 2014b).

The composition of the nasopharyngeal microbiome in infants and young children exhibits distinct patterns, with specific phyla and genera dominating at different stages. In infants, the most abundant phyla identified were Proteobacteria (39%), Actinobacteria (26%), Firmicutes (26%), and Bacteroidetes (6%) (Chonmaitree et al., 2017). The most abundant genera present at birth were *Staphylococcus* (22%)*, Corynebacterium* (5%)*, Lactobacillus* (4%)*, Gardnerella* (3%)*, Prevotella* (3%), and *Gemella* (3%) (Kelly et al., 2022) and in infants *Moraxella* (4-31%), *Corynebacterium* (3-28%)*, Streptococcus* (6-22%)*, Staphylococcus* (10-22%)*, Prevotella* (20%), *Dolosigranulum* (4-14%)*, Veillonella* (12%), *Haemophilus* (4-11%), and *Neisseria* (3%) (Teo et al., 2015; Bosch et al., 2016; Shilts et al., 2016; Bosch et al., 2017; Man et al., 2019a; Chapman et al., 2020; Shilts et al., 2020; Kelly et al., 2022). One prospective birth cohort study found higher abundances of *Acinetobacter, Gardnerella, Lactobacillus*, and *Sneathia* at birth compared with later periods during the first year of life (Kelly et al., 2022). Other prospective birth cohort studies reported that the most abundant species and genera at birth were *Streptococcus (S. viridans*), *Gemella*, *Staphylococcus*, and *Dolosigranulum* (Bosch et al., 2016; Bosch et al., 2017; Man et al., 2019a). On species level, *S. pneumoniae, H. influenzae, M. catarrhalis, Moraxella nonliquefaciens, Moraxella lincolnii, Dolosigranulum pigrum*, *Corynebacterium pseudodiphteriticum/propinquum*, *Streptococcus viridans* and *Staphylococcus aureus* were commonly identified in infants (Bosch et al., 2016; Bosch et al., 2017; Binia et al., 2021). Three studies reported a decrease in the abundance of *S. aureus* and an increase in the abundance of *Moraxella* (*M. catarrhalis/nonliquefaciens*), *Corynebacterium* (*C. propinquum*), *Dolosigranulum* (*D. pigrum*)*, H. influenzae*, and *S. pneumoniae* during the first six months of life *(* Biesbroek et al., 2014a; Bosch et al., 2016; Reyman et al., 2021*).* Another study reported a decrease in the abundance *Staphylococcus* and *Corynebacterium* and an increase in the abundance of *Dolosigranulum* and *Moraxella* during the first year of life (Teo et al., 2015). Yet another study a decrease in the abundance of *Corynebacterium, Dolosigranulum* and *Staphylococcus* and an increase of *Haemophilus* (Biesbroek et al., 2014b). At six weeks of age five different microbiome profiles were found dominated by either *Streptococcus, Moraxella, Staphylococcus, Corynebacterium* or *Corynebacterium/Dolosigranulum.* Afterwards the *Staphylococcus*-dominated profile disappeared, and a *Haemophilus*-dominated profile emerged, the *Corynebacterium/Dolosigranulum*-dominated profile was replaced by *Moraxella/Dolosigranulum*-dominated profile (Biesbroek et al., 2014b).

In studies including children less than 24 months of age, the most commonly identified phyla were Proteobacteria (64%), Firmicutes (21%), Bacteroidetes (11%), Actinobacteria (3%), and Fusobacteria (1.4%) (Bogaert et al., 2011) and the most commonly identified genera *Moraxella, Streptococcus, Haemophilus, Dolosigranulum, Corynebacterium*, *Staphylococcus*, *Flavobacteria*, *Prevotella, Pasteurella, Neisseria, Pseudomonas*, *Acinetobacter*, and *Helococcus* (Bogaert et al., 2011; Kinabo et al., 2013; Biesbroek et al., 2014b; Feazel et al., 2015; Mika et al., 2015; Chonmaitree et al., 2017; Salter et al., 2017; Boelsen et al., 2019; Tang et al., 2021*).* A prospective cohort study reported a higher abundance of *Staphylococcus* and *Corynebacterium* in the first 3 months of life compared with later timepoints during the first two years of life. The study also reported a decrease in the abundance of *Streptococcus* and an increase of unclassified *Flavobacteriaceae* during the first year of life and an increase in abundance of *Moraxella* in the first 21 months (Salter et al., 2017). A further prospective birth cohort study identified four different microbiome profiles in the nasopharyngeal microbiome during the first six months of life: a *Dolosigranulum/Corynebacterium-*, a *Moraxella-*, a *Staphylococcus-*, and a *Streptococcus*-dominated microbiome profile. At the age of two years, all children had mixed composition profiles, but many were dominated by *Moraxella* (Tang et al., 2021).

In toddlers, *Moraxella, Streptococcus, Haemophilus*, *Dolosigranulum* and *Corynebacterium* were identified on a genera level (Feazel et al., 2015; Verhagen et al., 2021), and *M. catarrhalis, S. pneumoniae, H. influenzae*, *D. pigrum, C. pseudodiphteriticum, S. aureus, M. nonliquefaciens, S. epidermidis*, and *S. mitis* on species or OTU level (Feazel et al., 2015; Tang et al., 2021). A prospective cohort study including children aged 6 to 17 years, reported that younger children had a higher abundance of *Dolosigranulum, Haemophilus*, and *Moraxella;* as well as *Ascochyta, Cladosporium*, and *Verticillium* in their nasopharyngeal microbiome, while older children had a higher abundance of *Corynebacterium* and *Staphylococcus* (McCauley et al., 2022).

#### Sex

The impact of sex on the composition of the nasopharyngeal microbiome in children has been investigated in several studies. One prospective birth cohort study reported a higher abundance of *Moraxella* in males in the first year of life during healthy periods but not during ARTI (Teo et al., 2015). Ten other studies did not find an association between sex and the composition of the nasopharyngeal microbiome in children (Hilty et al., 2012; Feazel et al., 2015; Shilts et al., 2016; Thapa et al., 2020; Boelsen et al., 2019; Man et al., 2019c; Liu et al., 2020; Zhou et al., 2020; Kelly et al., 2022*).*

#### Ethnicity

Studies exploring the influence of ethnicity on the nasopharyngeal microbiome in children reported varied findings. A prospective cohort study from the USA reported that non-Hispanic White children more frequently had a Hae*mophilus*-dominated microbiome profile compared with children from other ethnicities (Hasegawa et al., 2016; Hasegawa et al., 2017; Toivonen et al., 2018; Toivonen et al., 2019; Fujiogi et al., 2021). Another prospective cohort study from the USA found an association between ethnicity and the nasopharyngeal microbiome composition without specifying further details (McCauley et al., 2021). A retrospective cohort study from Fiji reported that infants of Indian descent had a higher diversity compared with Indigenous infants, while ethnicity was not associated with differences in richness (Boelsen et al., 2019). Furthermore, Indigenous infants had a higher abundance of *Moraxella*, *Haemophilus*, and *Helcococcus* and lower abundance of *Staphylococcus, Dolosigranulum*, and *Corynebacterium* compared with infants of Indian descent (Boelsen et al., 2019). Two other studies from the USA did not find an association between ethnicity and diversity or the composition of the nasopharyngeal microbiome (Shilts et al., 2016; Chonmaitree et al., 2017).

### Perinatal factors

#### Delivery mode

Multiple studies investigating the potential influence of delivery mode on the composition of the nasopharyngeal microbiome in children have reported a range of associations. A prospective birth cohort study reported that in the first year of life infants born by Caesarean section (CS) had a prolonged predominance of *Actinomyces, Granulicatella, Neisseria*, and *Prevotella* and a higher abundance of *Gemella* and *Streptococcus* in their nasopharyngeal microbiome, while infants born vaginally had a prolonged predominance of *Corynebacterium* and *Dolosigranulum*, and a late enrichment of *Moraxella* (Bosch et al., 2017). Another prospective birth cohort study reported that during the first six months of life infants born by CS had a higher abundance of *Gemella, S. aureus, S. viridans*, and *S. salivarius*, while infants born vaginally had a higher abundance of *C. pseudodiphteriticum/propinquum* and *D. pigrum*. Furthermore, infants born by CS stayed longer in a *S. aureus*-dominated microbiome profile, while infants born vaginally switched earlier to a *Moraxella*- or *Corynebacterium/Dolosigranulum-*dominated microbiome profile (Bosch et al., 2016). A third prospective birth cohort study reported an association between mode of delivery and the composition of the nasopharyngeal microbiome in the first six months of life without specifying further details (Reyman et al., 2021). A cross-sectional study reported a higher richness and diversity of the nasopharyngeal bacterial microbiome with a lower abundance of *Corynebacterium* and a higher abundance of *Staphylococcus* in infants born by CS compared with these born vaginally (Shilts et al., 2016).

Six other studies did not find an association between mode of delivery and the composition of the nasopharyngeal microbiome in the first year of life (Teo et al., 2015; Bosch et al., 2016; Binia et al., 2021) or at an older age (Thapa et al., 2020; Chonmaitree et al., 2017; Zhou et al., 2020).

#### Feeding method

The influence of breastfeeding on the composition of the nasopharyngeal microbiome in infants has been investigated in several studies, yielding diverse results and highlighting potential associations between breastfeeding and specific microbial taxa. A prospective birth cohort study observed an early abundance of *Dolosigranulum* and a prolonged predominance of *Corynebacterium* and *Dolosigranulum*, as well as a late enrichment of *Moraxella* in breastfed compared to formula-fed infants in the first year of life. In contrast, formula-fed infants were reported to have a higher abundance of *Gemella* and *Streptococcus* from birth onwards; and a higher abundance of *Streptococcus, Porphyromonas, Prevotella*, and *Veillonella* and a prolonged predominance of *Actinomyces, Granulicatella, Neisseria*, and *Prevotella* after one month of age (Bosch et al., 2017). Another prospective birth cohort study reported that being breastfed was associated with a higher abundance of *Corynebacterium* and lower abundances of *Haemophilus, Moraxella*, and *Streptococcus* during the first year of life (Kelly et al., 2022). A third prospective birth cohort study reported an association between being breastfed and the composition of the nasopharyngeal microbiome in the first six months of life without further specifying details (Reyman et al., 2021). A prospective cohort study reported a higher richness with a higher abundance of *Moraxella* in the nasopharyngeal microbiome in infants who were fed formula milk (Shilts et al., 2016). A retrospective cohort study reported that breastfed infants had a higher evenness, and more stable composition of their nasopharyngeal microbiome in the first two years of life (Biesbroek et al., 2014b). Furthermore, breastfeeding was associated more frequently with a *Corynebacterium/Dolosigranulum*-dominated microbiome profile and a higher abundance of *Corynebacterium* (*C. pseudodiphteriticum, C. propinquum, C. accolens, C. fastidiosum, C. segmentosum*) and *Dolosigranulum* (*D. pigrum*), while formula-fed infants were reported to have a higher abundance of *Actinomyces, Gemella, Granulicatella, Prevotella, Rothia*, *Staphylococcus*, and *Veillonella* at the age of six weeks, and a higher abundance of *Granulicatella* at the age of six months (Biesbroek et al., 2014b).

Eight other studies did not find an association between breastfeeding and richness, diversity or the composition of the nasopharyngeal microbiome in infants or older children (Teo et al., 2015; Thapa et al., 2020; Chonmaitree et al., 2017; Boelsen et al., 2019; Man et al., 2019c; Zhou et al., 2020; Binia et al., 2021; Henares et al., 2021). One prospective cohort study reported that there was no association between the presence of fucosylated oligosaccharides in breast milk and the composition of the nasopharyngeal microbiome in infants between two to four months of age (Binia et al., 2021).

### Environmental factors

#### Siblings and household size

The presence of siblings, particularly those under the age of five, has been investigated in relation to the nasopharyngeal microbiome composition in infants, revealing potential associations and accelerated microbiome maturation in the first year of life. A prospective birth cohort study reported that infants with siblings less than five years of age had an accelerated microbiome maturation and a higher abundance of *Pasteurellaceae* in their nasopharynx in the first year of life (Bosch et al., 2017). Another prospective birth cohort study found a higher abundance of *Haemophilus*, *Moraxella*, and *Streptococcus* and a lower abundance of *Staphylococcus* in infants who had older siblings at 12 months of age (Teo et al., 2015). A third prospective birth cohort study found an association between having siblings less than five years of age and the composition of the nasopharyngeal microbiome in the first six months of life without further specifying details (Reyman et al., 2021). A retrospective cohort study reported that in children under the age of 18 months, the abundance of *S. pneumoniae* was positively associated with increasing number of children in the household (Bogaert et al., 2011). A cross-sectional study found no association between household size and the composition of the nasopharyngeal microbiome in healthy children and children with otitis media (Coleman et al., 2021).

#### Day-care attendance

Day-care attendance during the first year of life has been examined in relation to the nasopharyngeal microbiome composition, revealing potential associations with specific microbial profiles, including increased abundance of certain bacteria such as *M. catarrhalis* and *H. influenzae*, as reported in prospective birth cohort studies. A prospective birth cohort study reported that in the first year of life day-care attendance was associated with an increase in the abundance of *M. catarrhalis* and *H. influenzae* in the nasopharynx (Accorsi et al., 2020). Another prospective birth cohort study observed that day-care attendance was associated with a higher abundance of *Haemophilus, Streptococcus*, and *Moraxella* and a lower abundance of *Staphylococcus* in the first year of life (Teo et al., 2015). A third prospective birth cohort study reported that day-care attendance was associated with an accelerated microbiome maturation and a higher abundance of *Moraxella* and a lower abundance of *Staphylococcus* (Bosch et al., 2017). A further prospective birth cohort study reported an association between day-care attendance and the composition of the nasopharyngeal microbiome in the first six months of life without further specifying details (Reyman et al., 2021). A prospective cohort study reported that infants in the first year of life who attended day-care attendance were more frequently colonised with *S. pneumoniae* (Xu et al., 2021b; Xu et al., 2021a). Three other studies did not find an association between day-care attendance and the composition of the nasopharyngeal microbiome (Bogaert et al., 2011; Hilty et al., 2012; Man et al., 2019c).

#### Pets

The influence of pets, particularly furry animals, on the nasopharyngeal microbiome during early life has been investigated in several studies, with varying results. One prospective birth cohort study reported that having furry pets was associated with a lower abundance of *Streptococcus* in the nasopharynx in the first year of life (Teo et al., 2015). Another prospective birth cohort study reported an association between having pets and the composition of the nasopharyngeal microbiome in the first six months of life without specifying further details (Reyman et al., 2021). A small prospective cohort study did not find an association between having pets and the composition of the nasopharyngeal microbiome (Shilts et al., 2016).

*Tobacco smoke exposure* The impact of tobacco smoke exposure on the composition of the nasopharyngeal microbiome has been a subject of limited research. There were only two studies which investigated the effect of tobacco smoke exposure on the composition of the nasopharyngeal microbiome. Both studies did not find an association (up to five months of age (Boelsen et al., 2019) or at 18 months of age (Bogaert et al., 2011)).

#### Season

The influence of seasonal variations on the composition of the nasopharyngeal microbiome has been investigated through several studies. These studies have provided insights into the association between different microbial profiles and specific seasons in healthy children and those with upper respiratory tract infections. Findings from these studies have been diverse, with some reporting significant associations between season and microbiome composition while others did not find any such relationship. A prospective cohort study from the USA reported that in either healthy children or children with an URTI an *Dolosigranulum/Corynebacterium*-dominated microbiome profile was more frequent during summer and autumn, while a *Haemophilus*-dominated microbiome profile was more frequent during winter and spring (McCauley et al., 2021). A prospective birth cohort study from Australia reported that during the first year of life in healthy infants or infants with an ARTI a *Haemophilus*-dominated microbiome profile was more frequent during spring/summer and a *Moraxella-*dominated microbiome profile more frequent during autumn/winter (Teo et al., 2015). A retrospective cohort study from the Netherlands observed a higher abundance of Proteobacteria, Fusobacteria, and Cyanobacteria during autumn/winter and a higher abundance of Bacteroides during spring. Furthermore, the study reported a higher abundance of *Bacillus, Brevibacillus, Flavobacterium*, and *Lactobacillus* during spring (Bogaert et al., 2011). On the species level a higher abundance of *B. fragilis* was found during spring (Bogaert et al., 2011). A prospective cohort study from the USA reported that children with asthma had a higher abundance of *Moraxella* in their nasopharynx during spring and a higher abundance of *Staphylococcus* during autumn (McCauley et al., 2022). In the same cohort, season had no influence on the fungal composition of the nasopharyngeal microbiome in children with asthma, expect during ARTI, when a higher abundance of *Malassezia* was found during spring and of *Candida* and *Cladosporium* during autumn (McCauley et al., 2022). Another prospective cohort study from the USA reported that in children with asthma, season did not influence the diversity of the nasopharyngeal microbiome but that a higher abundance of *Haemophilus* was found during summer (Pérez-Losada et al., 2017). Another reported an association between season and the overall microbiome composition without specifying further details (Reyman et al., 2021). Three other studies did not find an association between season and the composition of the nasopharyngeal microbiome (Boelsen et al., 2019; Man et al., 2019c; Coleman et al., 2021).

### Health-care associated factors

#### Vaccination

The impact of vaccination, particularly with pneumococcal conjugate vaccines (PCVs), on the composition of the nasopharyngeal microbiome has been investigated in several studies. These studies have explored the association between vaccination status and the presence or abundance of specific bacterial taxa in the nasopharynx during early childhood. However, findings from these studies have been inconsistent, with some reporting differences in colonization patterns or microbial diversity between vaccinated and non-vaccinated children, while others did not observe significant changes. A prospective cohort study found that children who were vaccinated with 7-valent pneumococcal conjugate vaccine (PCV7) were less frequently colonised with *Streptococcaceae* and *Corynebacteriaceae* in the nasopharynx during the first two years of life (Hilty et al., 2012). A prospective birth cohort study reported that infants who were vaccinated with PCV13 had a lower abundance of *S. pneumoniae* in the first year of life (Kelly et al., 2022). A cross-sectional study reported that children who were vaccinated with *H. influenzae* type b (Hib)/PCV10 had a higher richness but similar diversity of the nasopharyngeal microbiome compared with these who were not vaccinated (Salgado et al., 2020). The study did not find a difference in the abundance of bacteria between vaccinated and non-vaccinated children (Salgado et al., 2020). A prospective case-control study reported that children who were vaccinated with PCV had a higher diversity in their nasopharyngeal microbiome compared with these who were not vaccinated (Henares et al., 2021). A retrospective cohort study did not find differences in diversity, richness or overall composition of the microbiome between children who were vaccinated with PCV7 and these who were not. However, vaccinated children had a lower abundance of *S. pneumoniae* (Boelsen et al., 2019). Another retrospective cohort study reported that infants who were vaccinated with 3 doses of PCV7 had a higher diversity in their nasopharyngeal microbiome with a higher abundance of *Haemophilus, Staphylococcus*, *Veillonella, Prevotella, Bacteroidetes, Leptotrichia*, and *Streptococcus* at one year of age. At two years of age, no differences in the microbiome composition were found between vaccinated and non-vaccinated children (Biesbroek et al., 2014c). Further retrospective cohort studies did not find that Hib/PCV10 (Feazel et al., 2015) or PCV7 (Hilty et al., 2012; Biesbroek et al., 2014b) were associated with changes in diversity or composition of the nasopharyngeal microbiome.

#### Antibiotic exposure

The impact of antibiotic exposure on the nasopharyngeal microbiome in infants has been a subject of investigation in several prospective and retrospective cohort studies. These studies have explored the association between antibiotic use and the abundance and diversity of specific bacterial taxa in the nasopharynx during early life. However, findings from these studies have been varied, with some reporting changes in microbial composition following antibiotic exposure, while others did not observe significant associations. A prospective birth cohort study reported that infants who had been exposed to antibiotics (drug not specified) in the previous four months had a higher abundance of *Haemophilus*, *Streptococcus*, and *Moraxella* and lower abundance of *Dolosigranulum* and *Corynebacterium* in their nasopharynx (Teo et al., 2015). Another prospective birth cohort study reported that infants who had been exposed to antibiotics (mostly amoxicillin for seven to ten days) in the previous six months had a higher diversity and lower abundance of *Corynebacterium* and *Dolosigranulum* within seven days, *Enterobacter* within seven to 14 days, and *Staphylococcus* within 14 to 30 days after exposure, and a higher abundance of *Bifidobacterium* and *Firmicutes incertae sedis* within seven days after exposure (Chonmaitree et al., 2017). A third prospective birth cohort study, which did not specify the drug or time interval which was investigated, reported that antibiotic exposure was associated with a lower abundance of *Corynebacterium* and *Dolosigranulum* (Bosch et al., 2017). A further prospective birth cohort study reported that infants who were exposed to antibiotics during the first year of life (amoxicillin, metronidazole or trimethoprim/sulfamethoxazole) had a lower abundance of *Corynebacterium* and *Lactobacillus* and a higher abundance of *Haemophilus, Moraxella*, and *Streptococcus* in their nasopharynx (Kelly et al., 2022).

A prospective cohort study reported a lower abundance of *Moraxella* and a higher abundance of *Brachybacterium, Dolosigranulum*, and *Streptococcus* during antibiotic treatment (mostly oral amoxicillin given for LRTI) (Salter et al., 2017). Another prospective cohort study reported antibiotic exposure in the previous two months was associated with a lower abundance of *Moraxellaceae*, *Staphylococcaceae*, and *Streptococcaceae*, and a higher abundance *Pasteurellaceae* (Hilty et al., 2012). A prospective birth cohort study reported an association between exposure to antibiotics (drug not specified) in the 30 days prior to sampling and the composition of the nasopharyngeal microbiome in the first six months of life without further specifying details (Reyman et al., 2021). A randomised placebo-controlled trial reported a lower abundance of *Moraxella* after exposure to azithromycin for 14 days. A higher abundance of *Dolsigranulum* and *Corynebacterium* and a lower abundance of *Streptococcus* was observed after exposure to placebo for 14 days (Zhou et al., 2016).

A cross-sectional study reported that exposure to antibiotics in the three previous months was associated with a higher abundance of *Haemophilus* (Zhou et al., 2016). Several prospective cohort studies with overlapping participants reported that infants who were hospitalised in the first few months of life with bronchiolitis and exposed to antibiotics (drug not specified) more frequently had a *Haemophilus*-dominated microbiome profile and also had a higher a higher abundance of *H. influenzae* compared with infants who were hospitalised with bronchiolitis and did not receive antibiotics (Hasegawa et al., 2016; Hasegawa et al., 2017; Luna et al., 2018; Toivonen et al., 2018; Toivonen et al., 2019; Fujiogi et al., 2021; Raita et al., 2021).

A retrospective cohort study found no association between antibiotic exposure two weeks before sampling (drug not specified) and the composition of the nasopharyngeal microbiome (Boelsen et al., 2019), and a cross-sectional study between antibiotic exposure six months before sampling (drug not specified) (Man et al., 2019c). Two retrospective studies, which did not specify the time interval or drugs that were investigated, also did not find an association between antibiotic exposure and the composition of the nasopharyngeal microbiome (Bogaert et al., 2011; Feazel et al., 2015).

### Disease-associated factors

#### Acute respiratory tract infection

The nasopharyngeal microbiome composition in infants and children during ARTIs has been the focus of several prospective cohort and cross-sectional studies. A prospective cohort study reported that in infants during the first few months of life the most abundant genera in the nasopharynx during ARTI were *Moraxella*, *Streptococcus*, *Corynebacterium*, *Haemophilus*, and *Dolosigranulum* (Rosas-Salazar et al., 2016a). A cross-sectional study reported that the genera with the highest abundance in children under the age of two years with ARTI were *Streptococcus, Haemophilus*, and *Moraxella* (Salgado et al., 2020). Another prospective birth cohort study reported that children less than two years of age with an ARTI more often had a *H. influenzae-, M. catarrhalis-*, or *S. pneumoniae*-dominated microbiome profile, and less frequently a *D. pigrum-, C. pseudodiphtheriticum-, S. mitis-* or *Staphylococcus-*dominated microbiome profile than healthy children (Tang et al., 2021). Another prospective birth cohort study observed a lower abundance of *Corynebacterium* (in children less than three years of age) and of *Dolosigranulum* and *Staphylococcus* (in children less than four years of age) in children with ARTI compared with healthy children. Furthermore, a higher frequency of *Haemophilus-, Streptococcus-*, and *Moraxella-*dominated microbiome profiles were observed during ARTIs and a high abundance of one of these three bacteria was associated with a decrease in the diversity of the nasopharyngeal microbiome and an increase in severity of the ARTI (Teo et al., 2018). A third prospective birth cohort study reported that *Haemophilus-*, *Moraxella-* and *Streptococcus-*dominated microbiome profiles were more frequent during ARTI, while *Dolosigranulum-, Staphylococcus-*, and *Corynebacterium-*dominated microbiome profiles were less frequent during ARTI. Furthermore, *Neisseria* was more commonly found in nasopharyngeal samples taken during ARTI (Teo et al., 2015). A further prospective birth cohort study reported a decrease in presence and abundance of *Corynebacterium, Dolosigranulum*, and *Moraxella* and an increase in *Fusobacterium* and *Streptococcus* before and during an ARTI (Man et al., 2019a). On species level, the study found an increase in abundance of *Janthinobacterium lividum, Neisseria lactamica*, and *Prevotella nanceiensis* before and during ARTIs in infants (Man et al., 2019a).

A prospective birth cohort study reported that infants with frequent ARTIs during the first year of life had a less stable composition of the nasopharyngeal microbiome with a higher abundance of *Moraxella* and *Haemophilus* early in life, and a higher abundance of *Neisseria, Prevotella*, and *Alloprevotella* from the age of two months onwards. Infants with frequent ARTIs also had an absence or lower abundance of *Corynebacterium*, *Dolosigranulum*, and *Streptococcus* (Bosch et al., 2017). Another prospective birth cohort study observed an increase in the abundance of *Moraxella* one to two months before the occurrence of an ARTI. The study also reported a negative corelation between the abundance of *Streptococcus* closely matching to *S. gordonni* or *S. thermophilus/salivarius/vestibularis* and the risk for an ARTI (Teo et al., 2018). A third prospective birth cohort study reported that frequent ARTIs were associated with a higher abundance of *Moraxella* and lower abundance of *Dolosigranulum* and *Corynebacterium* (Teo et al., 2015). In another prospective birth cohort study a *H. influenzae*-dominated microbiome profile was associated with frequent ARTIs (Bosch et al., 2016). A cross-sectional study reported that ARTIs are more frequent in children colonised with *S. pneumoniae* (Kelly et al., 2018). while a prospective cohort study did not find an association between the composition of the nasopharyngeal microbiome and the risk for ARTI (Binia et al., 2021).

In a prospective cohort study of children with asthma a higher abundance of *Cladosporium* was associated with longer duration from baseline to the occurrence of an ARTI (McCauley et al., 2022). During a ARTI, a higher abundance of *Moraxella* and *Haemophilus* was associated with the presence of a virus in the nasopharynx (McCauley et al., 2022).

Two studies did not find an association between a prior ARTI and the composition of the nasopharyngeal microbiome (Man et al., 2019c; Binia et al., 2021).

#### Upper respiratory tract infection

The nasopharyngeal microbiome composition during URTIs in infants and children has been investigated in various prospective and retrospective cohort studies, as well as cross-sectional studies. A prospective birth cohort study found a higher abundance of *Haemophilus, Moraxella*, and *Streptococcus* and a lower abundance of *Myroides, Pseudomonas, Sphingomonas*, and *Yersinia* in infants less than nine months of age with URTI compared with healthy infants (Chonmaitree et al., 2017). A higher abundance of *Moraxella* and *Streptococcus* during URTI was associated with the presence of a virus (Chonmaitree et al., 2017). A retrospective cohort study found a higher abundance of *Haemophilus, Moraxella*, and *Streptococcus* and lower abundance of *Dolosigranulum* and *Corynebacterium* during URTI in infants at one year of age (Boelsen et al., 2019). A cross-sectional study reported that children with URTI had a lower abundance of *Staphylococcus* (*S. aureus*) and *Neisseriaceae* compared with healthy children (Coleman et al., 2021). Another cross-sectional study observed that in infants *Moraxella-* and *Streptococcus*-dominant microbiome profiles were more frequent during URTI than during healthy periods (Kelly et al., 2017). A further cross-sectional study reported a higher abundance of *Haemophilus, Moraxella*, and *Streptococcus* and a lower abundance of *Staphylococcus* during an URTI (Kelly et al., 2017). A prospective birth cohort study did not find an influence of an URTI on the diversity of the nasopharyngeal microbiome (Chonmaitree et al., 2017). A prospective cohort study reported that during an URTI the most abundant genera were *Haemophilus, Moraxella, Streptococcus Pseudomonas*, *Novosphingobium, Corynebacterium*, and *Dolosigranulum* (Haro et al., 2020).

A prospective birth cohort study reported that a higher diversity at one month of age was associated with frequent URTIs within the first six months of life without increasing the risk for acute otitis media (AOM) (Chonmaitree et al., 2017). A retrospective cohort study found that children with *Corynebacterium/Dolosigranulum*-dominated microbiome profile at six weeks and six months of age and *Moraxella-*dominated microbiome profiles at six weeks, six and 12 months had less frequent URTIs. The composition of the nasopharyngeal microbiome was more stable over time in children with less frequent URTIs (Biesbroek et al., 2014b).

A prospective birth cohort study reported that early colonisation with *Moraxella* was associated with an earlier occurrence of an URTI (Teo et al., 2015). Another prospective cohort study reported that in pre-school children a *Haemophilus*- and *Moraxella*-dominated microbiome profile was associated with an increased risk of URTIs and sinusitis, while an *Dolosigranulum/Corynebacterium*-dominated microbiome profile (which was also richer and more diverse) was associated with less frequent URTIs and sinusitis. Furthermore, children who developed URTI or sinusitis had a higher abundance of *Moraxella* and a lower abundance of *Prevotella, Acetobacteraceae*, and *Chryseobacterium* (McCauley et al., 2021).

A further prospective cohort study reported that children with URTI who were colonised with *S. pneumoniae* had a higher abundance of *Haemophilus* and *Streptococcus*, while children without URTI colonised with *S. pneumoniae* had a higher abundance of *Streptococcus* and a lower abundance of *Corynebacterium*_1 at one year of age (Xu et al., 2021b; Xu et al., 2021a). A retrospective cohort study reported that a higher abundance of *Dolosigranulum* was associated with less frequent URTIs, while a higher abundance of *Gemella* was associated with more frequent URTI (Biesbroek et al., 2014a).

#### Acute otitis media

The nasopharyngeal microbiome composition has been extensively investigated in relation to acute otitis media (AOM) and chronic otitis media, utilizing various prospective cohort studies, cross-sectional studies, and case-control studies. A prospective birth cohort study found that infants who develop AOM in the first year of life had a higher abundance of *Bifidobacterium*, *Enterobacter, Haemophilus*, and *Yersinia*, and a lower abundance of *Corynebacterium*, *Myroides*, and *Pseudomonas* in their nasopharynx. A higher abundance of *Staphylococcus* and *Sphingobium* was associated with a reduced risk of developing AOM after an URTI. Compared with healthy infants, no difference in diversity, but a higher abundance of *Haemophilus*, *Moraxella*, and *Streptococcus* was observed in the nasopharynx of infants with AOM. Furthermore, in infants with recurrent AOM a lower abundance of *Micrococcus* was found (Chonmaitree et al., 2017). Another prospective cohort study reported that compared with healthy children, children with AOM had a lower richness and diversity, but a higher density in their nasopharyngeal microbiome with a lower abundance of *Acidaminococcaceae, Comamonadaceae*, *Corynebacteriaceae*, and *Staphylococcaceae.* This study did not find an association between recurrent AOM and the composition of the nasopharyngeal microbiome (Hilty et al., 2012). A small prospective cohort study reported that in children with otitis media with effusion the most abundant genera in the nasopharynx were *Moraxella, Corynebacterium, Dolosigranulum, Haemophilus*, and *Streptococcus.* The presence of *Haemophilus* and *C. propinquum* was associated with anti-inflammatory mediators, while the presence of *Turicella* and *Dolosigranulum* was associated with pro-inflammatory mediators (Enoksson et al., 2020). Another prospective cohort study reported that in children with otitis media with effusion the most prevalent OTUs were *M. catarrhalis, H. influenzae, Streptococcus, Ornithobacterium, D. pigrum*, and *C. pseudodiphtheriticum* (Jervis-Bardy et al., 2015). A cross-sectional study reported that the abundances of *Acinetobacter, Klebsiella, Neisseria*, and *Haemophilus* were associated with longer duration of otorrhoea, while the abundances of *Corynebacterium, Dolosigranulum*, and *Haemophilus* were associated with shorter duration (Man et al., 2019b).

Another cross-sectional study reported that in children with recurrent AOM the most abundant genera were *Moraxella* (42%)*, Streptococcus* (20%)*, Haemophilus* (11%)*, Dolosigranulum* (17%), and *Corynebacterium* (9%) (Folino et al., 2021). A further cross-sectional study reported that there was no difference in diversity in children who had recurrent AOM compared with healthy children, but that the former had a higher abundance of *Moraxella.* Furthermore, children with middle ear effusion had a higher abundance of *Ornithobacterium* compared with children who never had AOM (Coleman et al., 2021). A cross-sectional study reported that children who were carrier of a variant of the fucosyltransferase genes, which encodes an enzyme involved in the production of the H antigen in body fluids and associated with an increased risk for AOM, had a higher abundance of *Cutibacterium* and a lower abundance of *Actinobacillus*, *Selenomonas*, and *Saccharibacteria*. Furthermore, children who were carrier of a Ras interacting protein 1 variant which is also associated with an increased risk for AOM, had a higher abundance of *Cutibacterium, Escherichia-Shigella*, and *Staphylococcus* and a lower abundance of *Acintobacillus* (Elling et al., 2021). Another cross-sectional study reported that there was no difference in diversity between children with recurrent AOM and children with atopy. However, the former had a lower abundance of *Dolosigranulum* and *Corynebacterium* and a higher abundance of *Haemophilus*, especially these children with recurrent AOM with tympanic membrane perforation (Folino et al., 2021). A prospective cohort study reported that compared with infants without AOM, these with recurrent AOM had a lower diversity of their nasopharyngeal microbiome at six but not 12 months of age. Infants with recurrent AOM had a lower abundance *Bacillus, Gemella, Fusobacterium, Prevotella*, and *Veillonella* and a higher abundance of *Moraxella* and *Dolosigranulum* (Xu et al., 2021b; Xu et al., 2021a). A cross-sectional study reported that compared with healthy children, children with recurrent AOM had a higher diversity in their nasopharyngeal microbiome with a lower abundance of *Corynebacterium* and *Dolosigranulum* and a higher abundance of *Neisseria, Gemella, Porphyromonas, Alloprevotella*, and *Fusobacterium* (Lappan et al., 2018).

A prospective case-control study reported that compared with healthy children, children with chronic otitis media had a lower diversity of their nasopharyngeal microbiome and more frequently had *Corynebacterium-, Moraxella*-, and *Streptococcus*-dominated microbiome profiles. Furthermore, a higher abundance of *H. influenzae*, *M. catarrhalis, Moraxella caprae*, and *S. pneumoniae*, and a lower abundance of *C. acnes*, *Capnocytophaga, Lactococcus, Lautropia*, *Neisseria*, two *Oxalobacteraceae* OTUs, and *Salmonella infantis* was found in children with chronic otitis media (Walker et al., 2019).

#### Lower respiratory tract infection

The nasopharyngeal microbiome has been extensively studied in infants and children with LRTIs, including bronchiolitis and pneumonia, providing valuable insights into the microbial profiles associated with these conditions. A prospective case-control study reported that in infants who were admitted to an intensive care unit with a LRTI the most abundant species in the nasopharynx were *M. catarrhalis/nonliquefaciens, H. influenzae/haemolyticus*, and *S. pneumoniae.* Compared to healthy children, children with LRTI more frequently had *H. influenzae/haemolyticus-* and *S. pneumoniae-*dominated microbiome profiles and less frequently *M. catarrhalis/nonliquefaciens-* and *C. propinquum/D. pigrum-*dominated microbiome profiles. Children with LRTI also had a higher abundance of *H. influenzae/haemolyticus, S. pneumoniae, Actinomyces, Prevotella* and a lower abundance of *Moraxella, C. propinquum, D. pigrum*, and *Helococcus* in the nasopharynx (Man et al., 2019c). A prospective birth cohort reported that early colonisation with *Streptococcus* was associated with earlier LRTI (Teo et al., 2015), while a prospective cohort study found that the acquisition of a new S*. pneumoniae* serotype was not associated with LRTI (Salter et al., 2017).

Several studies reported that during bronchiolitis the most abundant genera in the nasopharynx were *Streptococcus*, *Moraxella, Haemophilus* (Hasegawa et al., 2016; Hasegawa et al., 2017; Luna et al., 2018; Toivonen et al., 2018; Stewart et al., 2019; Toivonen et al., 2019; Fujiogi et al., 2021*)*, and *Prevotella* and *Staphylococcus* (Hasegawa et al., 2016; Hasegawa et al., 2017; Toivonen et al., 2018; Toivonen et al., 2019; Fujiogi et al., 2021*).* The studies identified between four to six different microbiome profiles: *Haemophilus-*, *Moraxella-, Streptococcus*-, *Staphylococcus*-, *Corynebacterium*-, *Enterobacter*-dominant profiles and a mixed profile (Hasegawa et al., 2016; Hasegawa et al., 2017; Luna et al., 2018; Toivonen et al., 2018; Mansbach et al., 2019; Toivonen et al., 2019; Fujiogi et al., 2021). A prospective cohort study reported *Haemophilus*-dominant microbiome profile associated with increased severity of bronchiolitis and length of hospital stay, while a *Moraxella*-dominant microbiome profile was associated with less frequent admission to an intensive care unit (Hasegawa et al., 2016; Hasegawa et al., 2017; Luna et al., 2018; Toivonen et al., 2018; Toivonen et al., 2019; Fujiogi et al., 2021).

A cross-sectional study reported that compared with healthy children, children with pneumonia more frequently had *Haemophilus-, Staphylococcus-*, and *Streptococcus*-dominant microbiome profiles, a higher abundance of *Haemophilus, Streptococcus, Escherichia*, and *Klebsiella* and a lower abundance of *Corynebacterium* and *Dolosigranulum* (Kelly et al., 2017). A prospective cohort study, reported that compared with healthy children, children with pneumonia had a lower diversity of their nasopharyngeal microbiome with a higher abundance of Firmicutes, *Mycoplasma, Streptococcus, Staphylococcus, Lactobacillus, Ralstonia, Acinetobacter*, and *Actinomyces*, and a lower abundance of Bacteroidetes, *Prevotella*, *Moraxella*, and *Dolosigranulum* (Lu et al., 2017; Dai et al., 2018). A prospective case-control study reported that children with pneumonia had a lower richness and diversity with a higher abundance of *Moraxella, Haemophilus*, and *Streptococcus* during non-viral pneumonia and a higher abundance of *Moraxella lacunata* during viral pneumonia (Sakwinska et al., 2014).

#### Viral infections

The nasopharyngeal microbiome plays a crucial role in respiratory viral infections, and several prospective cohort and cross-sectional studies have shed light on the microbial profiles associated with specific pathogens. Several prospective cohort studies reported that during RSV infection the most abundant genera in the nasopharynx were *Moraxella* (38-39%)*, Streptococcus* (20-27%)*, Staphylococcus* (27%), *Haemophilus* (11-14%)*, Corynebacterium* (5-19%), and *Dolosigranulum* (4-5%) (Rosas-Salazar et al., 2016b; Rosas-Salazar et al., 2018; Tan et al., 2023*).* Compared to healthy children, a lower richness and diversity at OTU level was reported. Children infected with RSV also had a lower abundance of *Staphylococcus*, and *Corynebacterium* and higher abundance of *Haemophilus, Moraxella*, and *Streptococcus* (Rosas-Salazar et al., 2016a; Rosas-Salazar et al., 2016b).

A higher abundance of *Moraxella* was found in children with RSV-A compared with children with RSV-B infection (Tan et al., 2023). A cross-sectional study reported that RSV infection was associated with a *S. pneumoniae-*dominated microbiome profile (Toivonen et al., 2019; Raita et al., 2021). Another cross-sectional study reported a delayed clearance of RSV after bronchiolitis in infants with a *Haemophilus*-dominated microbiome profile (Mansbach et al., 2019). A further cross-sectional study found an association between RSV viral load and the overall composition of the nasopharyngeal microbiome. A lower abundance of *Veillonella*, and a higher abundance of *Achromobacter* and *Haemophilus* was found in children infected with RSV compared with healthy children. The study found a positive correlation between the abundance of *Haemophilus* and C-X-C motif chemokine ligand 8 levels, which are indicative for a higher disease severity (Ederveen et al., 2018). A prospective birth cohort study reported that children who are colonised with *Dolosigranulum* had fewer RSV infections, especially RSV LRTIs (Teo et al., 2015).

A cross-sectional study reported that during rhinovirus infection the most abundant genera in the nasopharynx were *Streptococcus* (34%), *Moraxella* (19%), *Staphylococcus* (10%), *Burkholderia* (9%), *Neisseria* (6%), *Haemophilus* (6%), and *Janthinobacterium* (5%) (Perez et al., 2017). Another cross-sectional study reported that infants with rhinovirus infection had a lower abundance of *Streptococcus* compared with infants with other viral infections (Toivonen et al., 2019). Rhinovirus A infection was associated with a *Haemophilus*-dominant microbiome profile and rhinovirus C infection with a *Moraxella*-dominant microbiome profile (Toivonen et al., 2019; Raita et al., 2021). Children with a *Haemophilus*-dominated microbiome profile were more often infected with rhinovirus only (compared with co-infection with other or several viruses) (Hasegawa et al., 2016; Hasegawa et al., 2017; Toivonen et al., 2018; Toivonen et al., 2019; Fujiogi et al., 2021). A further cross-sectional study reported that rhinovirus infection was associated with a higher abundance of *Moraxella* (Tozzi et al., 2021).

A prospective cohort study reported that compared with healthy children, children with an influenza infection had a higher diversity with a lower abundance of *Moraxella, Staphylococcus, Corynebacterium*, and *Dolosigranulum*, and a higher abundance of *Phyllobacterium*, *Acinetobacter*, unclassified *Acidobacteria, Ralstonia, Pseudomonas, Lachnoclostridium*, and *Halomonas* (Wen et al., 2018; Zhou et al., 2020). Five microbiome profiles were identified: *Moraxella-,Streptococcus-, Staphylococcus-, Corynebacterium-*, and *Dolosigranulum-*dominant microbiome profiles (Zhou et al., 2020).

Another prospective cohort study reported that children infected with alpha coronaviruses more often had a *Haemophilus*-dominated microbiome profile compared with children infected with beta coronaviruses (Fujiogi et al., 2021).

A prospective cohort study reported that compared to healthy children, children with *Mycoplasma pneumoniae* pneumonia had a lower diversity, more often a *Staphylococcus*-dominated microbiome profile and a higher abundance of *Ralstonia* and *Acidobacteria* (Zhou et al., 2020).

A cross-sectional study reported that pertussis infection was associated with a higher abundance of *Alcaligenaceae* and *Achromobacter* (Tozzi et al., 2021).

#### Invasive pneumococcal disease

Invasive pneumococcal disease poses a significant health threat, and understanding the nasopharyngeal microbiome composition in affected children can provide valuable insights into the disease. A prospective case-control study reported that compared with healthy children, children who suffered from an invasive pneumococcal disease were more frequently colonised with *S. pneumoniae* and had a lower abundance of *D. pigrum* and *M. lincolnii* in their nasopharynx (Henares et al., 2021).

#### Atopy

The relationship between the nasopharyngeal microbiome and the development of allergic conditions has attracted significant attention, and several studies have explored this association. A prospective cohort study reported that children with atopy had a higher abundance of *Burkholderiaceae, Enterobacteriaceae, Sphingomonadaceae, Staphylococcaceae*, and *Xanthobacteraceae* in their nasopharynx compared with healthy children (Aydin et al., 2021). A prospective case-control study found that children with allergic rhinoconjunctivitis had a higher diversity of their nasopharyngeal microbiome and that there was an association between increasing diversity and disease severity (Yau et al., 2019). A cross-sectional study observed a lower diversity and lower abundance of *Corynebacterium* and *S. epidermidis* in children with cat allergy and a lower abundance of *Corynebacterium* in children with dog allergy. The study did not find association between bacterial composition and pollen allergy (Chun et al., 2021).

In a retrospective cohort study, healthy children with an increased risk for developing allergies were reported to have a higher abundance of *M. catarrhalis* in their nasopharynx (Chapman et al., 2020).

#### Wheezing

The relationship between the nasopharyngeal microbiome and wheezing, a common respiratory symptom in children, has been a subject of extensive investigation. During acute wheezing, a prospective cohort study more frequently observed a *S. pneumoniae*- and less frequently a *D. pigrum-*dominated microbiome profile in the nasopharynx of children (Tang et al., 2021).

In a prospective cohort study, a lower abundance of *Lactobacillus* and *Staphylococcus* during RSV infection was associated with an increased rate of recurrent wheezing at the age of two years (Rosas-Salazar et al., 2018). In a randomised double-blinded, placebo-controlled trial, which evaluated the effect of azithromycin in infants hospitalised with RSV bronchiolitis, a higher abundance of *Moraxella* in the nasopharynx was associated with higher rates of recurrent wheezing in the future (Zhou et al., 2016).

A prospective cohort study reported a higher abundance of Proteobacteria, *Burkholderiaceae, Enterobacteriaceae, Sphingomonadaceae, Staphylococcaceae*, and *Xanthobacteraceae*, as well as a higher abundance of *Haemophilus* (*H. influenzae*)*, Moraxella* (*M. catarrhalis*)*, S. aureus*, and *S. pneumoniae* in the nasopharynx of children with chronic wheezing (Aydin et al., 2021).

A prospective birth cohort found an association between a high abundance of *Streptococcus* before first the first ARTI and the development of chronic wheezing (Teo et al., 2015). Another prospective cohort study reported that children with a higher abundance of *Moraxella* or *Streptococcus* three weeks after hospitalisation for bronchiolitis and a higher abundance of *Streptococcus* in summer had a higher risk for recurrent wheezing at three years of age (Mansbach et al., 2020).

#### Asthma

The nasopharyngeal microbiome has emerged as a significant factor in the development and progression of asthma in children. A prospective cohort study reported that compared with healthy children, children with asthma had a higher abundance of Firmicutes and *Staphylococcaceae* in their nasopharynx (Aydin et al., 2021). Another prospective cohort study reported that the most abundant genera in the nasopharynx of children with asthma were *Moraxella* (35%), *Staphylococcus* (14%), *Dolosigranulum* (9%), *Corynebacterium* (9%), *Prevotella* (6%), *Streptococcus* (5%), *Haemophilus* (4%), and *Fusobacterium* (3%) (Pérez-Losada et al., 2017). A third prospective cohort study reported that children with asthma had a stable composition of their nasopharyngeal microbiome over time despite viral infections or exacerbations. Six microbiome profiles were identified: *Moraxella-, Staphylococcus-, Corynebacterium-, Streptococcus-, Dolosigranulum*-, and *Haemophilus*-dominated profiles. *Moraxella*-dominated microbiome profiles were associated with an increased risk for exacerbation, while *Staphylococcus*- or *Corynebacterium*-dominated microbiome profiles were associated with reduced risk for respiratory illness and exacerbations (McCauley et al., 2019). A further prospective cohort study reported that the most abundant genera in the nasopharynx of children with asthma were *Moraxella* (28%), *Staphylococcus* (18%), *Corynebacterium* (10%), *Dolosigranulum* (8%), *Prevotella* (6%), *Streptococcus* (6%), *Fusobacterium* (3%), and *Haemophilus* (3%). The study found an association between the microbiome composition of the nasopharynx and the asthmatic phenotype. Children who were older when they were diagnosed with asthma, had persisting symptoms despite treatment or a higher body mass index (BMI) had a higher abundance of *Corynebacterium* and *Prevotella* and a lower abundance of *Moraxella* and *Dolosigranulum*. Children who were youngest when they were diagnosed, had a high rate of positive allergen tests, high blood eosinophil and immunoglobulin E levels and a high rate of needing inhaled corticosteroids had a lower diversity and a higher abundance of *Moraxella* and a lower abundance of *Corynebacterium, Staphylococcus*, and *Prevotella*. Children with a lower BMI, low rate of positive skin prick test and a better response to treatment with bronchodilators had an intermediate abundance of the main genera (Pérez-Losada et al., 2018). Another prospective cohort study, reported that a higher abundance of *Moraxella* and *Haemophilus* was associated with asthma exacerbations (McCauley et al., 2022). Furthermore, a further prospective cohort study a lower diversity was observed during an asthma exacerbation with a higher abundance of *Moraxella.* Additionally, metabolic pathways associated with *Moraxella* (methane, ketone bodies, and vitamin B3 metabolism) were enhanced during an exacerbation (Hou et al., 2022). In the same study, *Dolosigranulum-* and *Corynebacterium* 1*-*dominated microbiome profiles were more frequent at baseline and in healthy controls compared with children during an asthma exacerbation (Hou et al., 2022).

A high abundance of *H. influenzae* and *S. pneumoniae* during hospitalisation with severe bronchiolitis in infancy was associated with a higher risk of developing asthma at the age of five years, while a high abundance of *M. nonliquefaciens* was associated with lower risk (Raita et al., 2021). Children with reversible airway obstruction had a higher abundance of *Haemophilus* and *S. pneumoniae* and lower abundance of *Moraxella, Corynebacterium, Dolosigranulum*, and *Staphylococcus* at the age of six years (Man et al., 2020). A cross-sectional study reported a negative correlation between the abundance of *Corynebacterium* and *S. epidermidis* and the expression of genes involved in inflammatory processes (Chun et al., 2021).

#### Positive pressure ventilation

The composition of the nasopharyngeal microbiome in infants during hospitalization for bronchiolitis has been linked to the severity of the respiratory condition. In a cross-sectional study, infants requiring positive pressure ventilation exhibited distinct microbial profiles in their nasopharynx compared to those who did not require such intervention. A cross-sectional study reported that infants needing positive pressure ventilation during hospitalisation for bronchiolitis had a higher abundance of *Haemophilus, Klebsiella, Rothia*, and *Streptococcus*, and a lower abundance of *M. catarrhalis* in their nasopharyngeal microbiome. Sphingolipid metabolites were enriched in infants needing positive pressure ventilation and correlated to the abundance of *S. pneumoniae* (Stewart et al., 2017). The abundance of *Streptococcus* positively correlated with metabolites (glucuronate and 1-palmitoyl-2-palyitoleoyl-GPC 16:0/16:1) associated with a higher risk of needing positive pressure ventilation and negatively correlated with metabolites (plasmalogen sub-pathway) associated with a lower risk of needing positive pressure ventilation, for the abundance *Moraxella* an opposite correlation was found (Stewart et al., 2019).

### Other factors

The composition of the nasopharyngeal microbiome in infants has been found to be influenced by various factors and has implications for their respiratory health. A prospective cohort study reported that infants with low vitamin D levels had a lower richness and diversity and a higher abundance of *Staphylococcus* in their nasopharyngeal microbiome. In infants with low vitamin D levels a *Haemophilus-*dominant microbiome profile was associated with a higher risk of intensive care admission during hospitalisation with bronchiolitis (Toivonen et al., 2018). A cross-sectional study reported that infants who were perinatally exposed to HIV had a higher abundance of *Klebsiella* in their nasopharynx (Kelly et al., 2017). A prospective birth cohort study reported an association between pacifier use and composition of the nasopharyngeal microbiome in the first 6 months of life without further specifying any details (Reyman et al., 2021). A randomised, placebo-controlled trial reported that children who were born preterm and had received palivizumab had a lower frequency of *Staphylococcus*-dominated microbiome profiles and a higher abundance of biomarker species, such as *Klebsiella*, as well as a more diverse set of oral taxa, including *Streptococcus* in their nasopharynx at one year of age. Furthermore, they had a higher abundance of *Haemophilus* and a lower abundance of *Moraxella* and *Neisseriaceae* at six years of age (Man et al., 2020).

### Collection method

The collection method used to assess the nasopharyngeal microbiome in children can have a impact on the observed microbial composition. In children with asthma a higher diversity and a higher mean number of OTUs was found in samples taken by nasal brushes compared to nasal washes. Furthermore, a higher abundance of *Bacteroides* and *Pseudomonas* and a lower abundance of *Haemophilus, Fusobacterium, Moraxella, Prevotella, Staphylococcus, Streptococcus*, and *Treponema* was found in samples taken by nasal brushes (Pérez-Losada et al., 2016). A study which compared results from nasal filters with nasal washed found very similar results. The only genus that was differently abundant between the two collection methods was *Sphingobium* (Shilts et al., 2020).

### Interaction between bacteria

An early presence and high abundance of *Moraxella, Dolosigranulum*, and *Corynebacterium*, as well as *Moraxella*-, *Dolosigranulum-, Haemophilus-*, and *Streptococcus*-dominated microbiome profiles have been reported to be associated with a more stable composition of the nasopharyngeal microbiome over time (Biesbroek et al., 2014b; Hou et al., 2022; Kelly et al., 2022). In contrast, a high abundance of *Streptococcus, Haemophilus*, and *Bacteroidetes*, and a *H. influenzae*-dominated microbiome profile have been reported to be associated with a less stable microbial composition over time (Biesbroek et al., 2014b; Bosch et al., 2016; Kelly et al., 2022). Colonisation with *M. catarrhalis, S. pneumoniae*, and *H. influenzae* have been associated with a lower bacterial diversity (Chonmaitree et al., 2017; Chapman et al., 2020).

A positive correlation has been found between the abundance of *Dolosigranulum* (*D. pigrum*) and *Corynebacterium* (*C. pseudodiphteriticum*) (Biesbroek et al., 2014a; Lappan et al., 2018; Coleman et al., 2021), *Moraxella* (Coleman et al., 2021), and *Neisseriaceae* (Coleman et al., 2021). A positive correlation has also been observed between the abundance of *Streptococcus* (*S. pneumoniae*) and *Moraxella* (Kelly et al., 2018; Boelsen et al., 2019), *Haemophilus* (Boelsen et al., 2019; Coleman et al., 2021), *Corynebacterium* (including *C. accolens*) (Salter et al., 2017), and *Staphylococcus* (Salter et al., 2017). Furthermore, a positive correlation between the abundance of *Corynebacterium* (including *C. accolens*) and *Staphylococcus* (Salter et al., 2017), and between the abundance of *Ornithobacterium* and *Helococcus*, *Dichelobacter*, and *Cardiobacteriaceae* has been reported (Coleman et al., 2021). Furthermore, a positive correlation has also been observed between the abundance of *Haemophilus* and *Moraxella*, and *Gemella* and *Porphyromonas* and *Neisseria* (Lappan et al., 2018), as well as between the abundance of *Veillonella* and *Streptococcus, Prevotella* and *Alloprevotella* (**Figure 2**) (Hasegawa et al., 2016; Hasegawa et al., 2017; Toivonen et al., 2018; Toivonen et al., 2019; Fujiogi et al., 2021). On species level, positive correlations between the abundance of *M. nonliquefaciens* and *S. pneumoniae, H. influenzae* and *M. catarrhalis* (Binia et al., 2021), and between *Acinetobacter*, and *Streptococcus parasanguinis, Streptococcus salivarius*, and *Veillonella* have been reported (Accorsi et al., 2020).

Negative correlations have been reported between the abundance of *Corynebacterium* (*C. pseudodiphtheriticum/propinquum, C. accolens/macginleyi*, and *C. tuberculostearicum*), *Dolosigranulum*, and *Streptococcus* (including *S. pneumoniae*) (Biesbroek et al., 2014b; Biesbroek et al., 2014a; Salter et al., 2017; Kelly et al., 2018; Kelly et al., 2022), *Staphylococcus* (Biesbroek et al., 2014b; Biesbroek et al., 2014a; Salter et al., 2017)*, Moraxella* (Biesbroek et al., 2014a)*, Veillonella* (Biesbroek et al., 2014a)*, Rothia* (Biesbroek et al., 2014a)*, Granulicatella* (Biesbroek et al., 2014a)*, Actinomyces* (Biesbroek et al., 2014a) *Prevotella* (Biesbroek et al., 2014a)*, Gemella* (Biesbroek et al., 2014a)*, Leptotrichia* (Biesbroek et al., 2014a)*, Fusobacterium* (Biesbroek et al., 2014a), and *Klebsiella* (Biesbroek et al., 2014a). A negative correlation has also been reported between the abundance of *Streptococcus* (Hasegawa et al., 2016; Hasegawa et al., 2017; Toivonen et al., 2018; Toivonen et al., 2019; Fujiogi et al., 2021) and *Haemophilus* (Hasegawa et al., 2016; Hasegawa et al., 2017; Toivonen et al., 2018; Toivonen et al., 2019; Fujiogi et al., 2021)*, Moraxella* (Hasegawa et al., 2016; Hasegawa et al., 2017; Toivonen et al., 2018; Toivonen et al., 2019; Fujiogi et al., 2021)*, Staphylococcus* (Salter et al., 2017; Kelly et al., 2018), and *Pseudomonas* (**Figure 2**) (Boelsen et al., 2019). Children colonised with *S. pneumoniae* have been reported to have a lower abundance of *Actinomyces, Prevotella, Dolosigranulum, Veillonella, Corynebacterium*_1*, Gemella*, and *Anoxybacillus* (Xu et al., 2021b; Xu et al., 2021a). On species level, a negative correlation has been observed between the abundance *D. pigrum* and the acquisition of *S. aureus* (Accorsi et al., 2020). Furthermore, children not colonised with *S. pneumoniae, H. influenzae* or *M. catarrhalis* have been reported to have a higher abundance of *Lactococcus lactis* subsp. *cremoris, Cutibacterium acnes, Moraxella osloensis, Acinetobacter pittii, C. accolens, Staphylococcus hominis*, *Staphylococcus epidermidis, S. aureus, S. viridans, Staphylococcus haemolyticus, D. pigrum*, and *Staphylococcus arlettae* (Binia et al., 2021).

## Discussion

The findings of this comprehensive review show that the nasopharyngeal microbiome in children is dynamic and influenced by many extrinsic factors. Colonisation of the nasopharynx with certain bacteria is a risk factor for developing infections and other diseases, while other bacteria are protective (Salter et al., 2017). A high abundance of *Haemophilus*, *Moraxella*, and *Streptococcus* and a low abundance of *Corynebacterium* and *Dolosigranlum* are associated with respiratory tract infections (including AOM, bronchiolitis and pneumonia), wheezing and asthma exacerbations (Teo et al., 2015; Hasegawa et al., 2016; Rosas-Salazar et al., 2016a; Zhou et al., 2016; Chonmaitree et al., 2017; Hasegawa et al., 2017; Kelly et al., 2017; Pérez-Losada et al., 2017; Luna et al., 2018; Pérez-Losada et al., 2018; Teo et al., 2018; Toivonen et al., 2018; Boelsen et al., 2019; Man et al., 2019a; Stewart et al., 2019; Toivonen et al., 2019; Mansbach et al., 2020; Salgado et al., 2020; Aydin et al., 2021; Fujiogi et al., 2021; Tang et al., 2021). Importantly, this review also shows that the extrinsic factors identified as risk factors for these adverse health outcomes (Schuez-Havupalo et al., 2017; Sandall et al., 2018; Branger et al., 2022; Duong et al., 2022), are associated with the aforementioned changes in the nasopharyngeal microbiome. This includes being born by CS (Bosch et al., 2016; Shilts et al., 2016), not being breastfed (Biesbroek et al., 2014b; Shilts et al., 2016; Kelly et al., 2022), antibiotic exposure (Teo et al., 2015; Hasegawa et al., 2016; Thapa et al., 2020; Chonmaitree et al., 2017; Hasegawa et al., 2017; Salter et al., 2017; Luna et al., 2018; Toivonen et al., 2018; Toivonen et al., 2019; Fujiogi et al., 2021; Raita et al., 2021; Kelly et al., 2022), having siblings (Teo et al., 2015), and day-care attendance (Teo et al., 2015; Xu et al., 2021b; Xu et al., 2021a).

Understanding the dynamics, composition, and function of the nasopharyngeal microbiome in healthy children is a prerequisite to investigate the role of the microbiome in children with diseases. The presence or abundance of an individual bacteria affects those of others due to ecologic interactions. Research on the pathogenesis and prevention of diseases should, therefore, not only focus on pathogens but also on commensals, and even more importantly on interactions between bacterial communities, as well as interactions with the immune system and metabolism. In addition to identifying microbial composition, identification of microbial community function is likely even more important.

The interaction between bacteria within the microbiome can be direct, such as competition for nutrients or receptors (for example iron or epithelial bindings sites) or metabolites (antimicrobial products such as bacteriocins), or indirect trough other bacteria or the immune system (Hertli and Zimmermann, 2022). Several studies have found a negative association between the abundance of *Corynebacterium* and *Streptococcus* (*S. pneumoniae)* (Laufer et al., 2011; Biesbroek et al., 2014b; Biesbroek et al., 2014a; Teo et al., 2015; Bomar et al., 2016; Salter et al., 2017; Kelly et al., 2018; Kelly et al., 2022). *C. accolens* hydrolyses human triacylglycerols into free fatty acids which have antimicrobial activity and inhibit growth of *S. pneumoniae* (Bomar et al., 2016; Kelly et al., 2022). *In vitro* studies have also shown that *C. propinquum* and *C. pseudodiphtheriticum* inhibit growth of *S. pneumoniae* (Xu et al., 2021b). *Corynebacterium* is often found together with *Dolosigranulum. Dolosigranulum* is thought to lower the pH trough the production of lactic acids, creating a more favourable condition for *Corynebacterium* to grow (de Steenhuijsen Piters et al., 2015). A negative relationship has also been described between the abundance of *Streptococcus* and *Staphylococcus* (Salter et al., 2017; Kelly et al., 2018), more specifically between *S. pneumoniae* and *S. aureus* (Veenhoven et al., 2003; Bogaert et al., 2004; Regev-Yochay et al., 2004; McNally et al., 2006; Watson et al., 2006; Zemlicková et al., 2006; Madhi et al., 2007; Pettigrew et al., 2008). It has been hypothesised that *S. pneumoniae* inhibits the growth of *S. aureus* trough the production of hydrogen peroixide (Pericone et al., 2000; Regev-Yochay et al., 2006; Park et al., 2008). *In vitro*, hydrogen peroxide produced by *S. pneumoniae* has been shown to be bactericidal against *H. influenzae, N. meningitidis*, and *M. catarrhalis* (Pericone et al., 2000). However, in clinical studies, both negative (Madhi et al., 2007; Pettigrew et al., 2008) and positive (Jacoby et al., 2007; Pettigrew et al., 2008; Shiri et al., 2013; Feazel et al., 2015; Boelsen et al., 2019) correlations between the abundance of *S. pneumoniae* and *H. influenzae* have been described.

Most respiratory tract infections are polymicrobial and the bacteria involved in the infection influence each other. In AOM, for example, co-infection with *H. influenzae* and *M. catarrhalis* leads to an increased resistance of biofilms to antibiotics and to a decreased host clearance (Armbruster et al., 2010). Vaccination against one species (e.g. *H. influenzae*), might therefore also impact colonisation or infection with another species (e.g. *M. catarrhalis)*, as the bacteria might be easier cleared by the host if not in a biofilm maintained by the other bacteria.

In addition to biofilm production, bacteria might also influence each other through interaction with the immune system. In infants, a *Haemophilus*-dominant microbiome profile, has been associated with upregulated T helper (TH)2 and TH17-type inflammatory response in the airways (Hasegawa et al., 2016). In a murine study, colonisation of the nasopharynx by either *H. influenzae* or *S. pneumoniae* results in persistent colonisation. However, co-administration of these bacteria leads to rapid clearance of *S. pneumoniae* from the nasopharynx. This clearance is likely attributed to enhanced opsonophagocytic killing, as components of *H. influenzae* stimulate complement-dependent phagocytic elimination of *S. pneumoniae* (Lysenko et al., 2005). Consequently, this raises the question of whether underlying host factors, such as immunity parameters, independently drive both outcomes, thereby compromising microbiome stability and increasing susceptibility to infections. Changes in the nasopharyngeal microbiome can be observed before ARTIs with a decrease of beneficial microbes and increased abundance of potential pathogenic bacteria with an influx of oral species into the nasopharyngeal (Man et al., 2019a).

Furthermore, there are also interactions between viruses and bacteria. Respiratory viruses disrupt the nasopharyngeal microbiome and lead to a higher abundance of *Haemophilus, Moraxella*, and *Streptococcus* (Rosas-Salazar et al., 2016a; Rosas-Salazar et al., 2016b; Ederveen et al., 2018; Toivonen et al., 2019; Raita et al., 2021; Tozzi et al., 2021). On the other hand, children colonised with *Dolosigranulum* have fewer RSV infections, especially RSV LRTIs (Teo et al., 2015). RSV has been reported to increase the virulence of *S. pneumoniae* (Smith et al., 2014) and influenza adhesion of *S. pneumoniae* to host cells (Avadhanula et al., 2006).

In addition to URTIs, the nasopharyngeal microbiome also influences the risk for LRTIs, including pneumonia (Sakwinska et al., 2014; Kelly et al., 2017; Lu et al., 2017; Dai et al., 2018). Pneumonia is the leading cause of childhood death globally and a leading cause of hospitalization in children in developed countries (World Health Association, [[NoYear]]; Jain et al., 2015). Children with pneumonia have lower diversity of their nasopharyngeal microbiome with a higher abundance of *Moraxella, Haemophilus, Staphylococcus*, and *Streptococcus* (Sakwinska et al., 2014; Kelly et al., 2017; Lu et al., 2017; Dai et al., 2018). Microbiome manipulations that enhance stability of the upper airway microbiome in infants could conceivably diminish childhood pneumonia.

A Cochrane meta-analysis shows that oral administration of probiotics (mainly *Lactobacillus* and *Streptococcus*-containing products) decreases the number of AOM and other respiratory tract infection in healthy children, but not in children with recurrent AOM (Scott et al., 2019). However, the range of species that have been tested is limited and includes mostly bacteria deriving from the intestine. Furthermore, the studies included in the review did not investigate local administration of probiotics to the nasopharynx. Probiotics are able to modulate the epithelial barrier function. For *Lactobacillus*, for example, it has been shown that it promotes the expression and regulation of tight junctions and adherence junctions, resulting in the restoration of a defective epithelial barrier. Furthermore, probiotic bacteria also interact with the immune system through pattern recognition receptors, such as Toll-like receptors, which upon activation can stimulate or suppress various immune responses (Martens et al., 2018). Further studies are needed to investigate, nasopharyngeal microbiome-manipulation in prevention and treatment of respiratory disease.

In the studies included in this review, antibiotic exposure has been associated with a lower abundance of bacteria in the nasopharynx which have been associated with respiratory health such as *Dolosigranulum* (Teo et al., 2015; Bosch et al., 2017; Chonmaitree et al., 2017), and *Corynebacterium* (Teo et al., 2015; Bosch et al., 2017; Chonmaitree et al., 2017; Kelly et al., 2022), but an increase in genera associated with ARTI such as *Haemophilus* (Teo et al., 2015; Hasegawa et al., 2016; Thapa et al., 2020; Hasegawa et al., 2017; Luna et al., 2018; Toivonen et al., 2018; Toivonen et al., 2019; Fujiogi et al., 2021; Raita et al., 2021; Kelly et al., 2022), and *Moraxella* (Teo et al., 2015; Kelly et al., 2022). However, there are other studies which show that children treated with erythromycin are less commonly colonised by *S. pneumoniae* and *M. catarrhalis*, while colonisation by *H. influenzae* remains unchanged (Sundberg et al., 1984). Furthermore, even a single dose of ceftriaxone has been shown to significantly decrease the abundance of *M. catarrhalis, H. influenzae*, and *S. pneumoniae* in the nasopharynx (Heikkinen et al., 2000), and ten days of amoxicillin/clavulanate leads to an even greater decrease in the abundance of *M. catarrhalis* and *H. influenzae* (Cohen et al., 1999). As with the intestinal microbiome (Zimmermann and Curtis, 2019).

the effect of antibiotics on the nasopharyngeal microbiome likely depends on the spectrum of activity, route of administration, as well as dose and duration of administration.

## Conclusions and future directions

The association between the composition of the nasopharyngeal microbiome and health outcomes in children is intriguing and highlights the potential of the nasopharyngeal microbiome as a marker for identifying children at risk for disease and even more importantly, as an avenue for targeted interventions and preventive strategies.

Future studies should include larger numbers of participants and longitudinal follow-up. Samples need to be sequenced sufficiently deep and/or combine short- with long-read sequencing approaches. Standardising methods and reporting will help comparison and pooling of results from different studies. This will enable stronger quantitative analysis of the contribution of each risk factor.

## Limitations

One of the main limitations of current studies that have investigating the nasopharyngeal microbiome in children is the large overlap of participants (Bogaert et al., 2011; Biesbroek et al., 2014b; Biesbroek et al., 2014c; Biesbroek et al., 2014a; Teo et al., 2015; Bosch et al., 2016; Hasegawa et al., 2016; Pérez-Losada et al., 2016; Rosas-Salazar et al., 2016a; Rosas-Salazar et al., 2016b; Bosch et al., 2017; Hasegawa et al., 2017; Kelly et al., 2017; Lu et al., 2017; Pérez-Losada et al., 2017; Stewart et al., 2017; Dai et al., 2018; Kelly et al., 2018; Luna et al., 2018; Pérez-Losada et al., 2018; Rosas-Salazar et al., 2018; Teo et al., 2018; Toivonen et al., 2018; Wen et al., 2018; Man et al., 2019a; Mansbach et al., 2019; Stewart et al., 2019; Toivonen et al., 2019; Mansbach et al., 2020; Zhou et al., 2020; Fujiogi et al., 2021; Raita et al., 2021; Reyman et al., 2021; Xu et al., 2021b; Xu et al., 2021a; Tan et al., 2023). Furthermore, many of the studies were underpowered and might therefore not have identified factors that influence the composition of the nasopharyngeal microbiome. Furthermore, as with all microbiome studies, it is difficult to account for effect modifiers. For example, differences in the nasopharyngeal microbiome between breastfed and formula-fed infants might contribute to the protective effect of breastfeeding from respiratory tract infections (Biesbroek et al., 2014a). However, formula-fed infants more often have older siblings and are more often exposed to tobacco smoke (Biesbroek et al., 2014a). Similarly, infants born by CS, are more often exposed to antibiotics. Moreover, geographic location will likely also influence the composition of the nasopharyngeal microbiome.

Other limitations include those associated with diagnostics. Results obtained by metagenomic sequencing are influenced by sampling methods, DNA extraction, library preparation, as well as by the choice of primers, sequencing method and platform, bioinformatic pipelines and tools. In 16S rRNA sequencing, usually only a short segment of the gene is sequenced, which can lead to incomplete taxonomic identification. The choice of variable (V) region can significantly impact results and interpretation of microbial community analysis. Different V regions have varying levels of sequence conservation and discriminatory power, leading to differences in taxonomic resolution and the ability to detect specific microbial taxa. Identification by the use of references databases relieson previously sequenced bacteria, hence the use of different databases also influences results It is known that *Dolosigranulum* is misclassified as *Alloiococcus* in the GreenGenes database and therefore *Dolosigranulum* was used throughout the manuscript.

These limitations partly explain why some studies report conflicting results (as summarised in **Table 1** and **Figure 2**) and that further research to clarify the association between different components of the nasopharyngeal microbiome and external factors are needed.

## Author contributions

PZ did the literature research, wrote the manuscript, and designed the figures.

## Supplementary material

Supplementary Table 1 Summary of findings of studies investigating the nasopharyngeal microbiome in children using next generation sequencing.
