# Supplementary material for: Exploring the microbial landscape of the nasopharynx in children: a systematic review of studies using next generation sequencing
Source: Front Microbiomes. 2023 Oct 19;2:1231271. doi: 10.3389/frmbi.2023.1231271 (PMC12993585; doi:10.3389/frmbi.2023.1231271)
Supplement: Supplementary file 1 [file DataSheet_1.pdf]

Supplementary data

Search terms

(*bacteria/de, ge, cl, gd, jp or *microbiome/ or (microbiome* or microbiome*).tw,kf. or DNA Bacterial/ge or DNA Ribosomal/ge or *RNA Ribosomal 16S/ge or *Sequence Analysis DNA/ or *Carrier State/mi) AND (Nose/de or Nasal microbiome.mp. or *Nose/mi or *Nasopharyngeal Diseases/ep, mi or Nasopharynx/mi or Respiratory Tract Infections/mi [mp=ti, ab, hw, tn, ot, dm, mf, dv, kf, fx, dq, nm, ox, px, rx, an, ui, sy]) and ((newborn* or new-born* or baby or babies or neonat* or neo-nat* or infan* or toddler* or pre-schooler* or preschooler* or kinder or kinders or kindergarten* or kinder-aged or boy or boys or girl or girls or child or children or childhood or pediatric* or paediatric* or adolescen* or youth or youths or teen or teens or teenage* or school-age* or schoolage* or school-child* or schoolchild* or school-girl* or schoolgirl* or school-boy* or schoolboy*).af.
