# Supplementary material for: Exploring the microbial landscape of the nasopharynx in children: a systematic review of studies using next generation sequencing
Source: Front Microbiomes. 2023 Oct 19;2:1231271. doi: 10.3389/frmbi.2023.1231271 (PMC12993585; doi:10.3389/frmbi.2023.1231271)
Supplement: Supplementary Table 1 — Summary of findings of studies investigating the nasopharyngeal microbiome in children using next generation sequencing. [file Table_1.pdf]

Supplementary table Summary of findings of studies investigating the nasopharyngeal microbiome in children using next generation sequencing

**Author**<br><br>**Country**<br><br>**Publication year**	**Study type (level of evidence)**	**Total no of children, total no of swabs**<br>**% male**	**Clinical condition at sample collection (no of children, no of swabs)**<br>**Age and/or sampling time points**<br><br>**Sample type, swab/medium**<br>**Storage conditions**<br><br>**DNA extraction kit**<br>**Primers**<br>**Sequencing technique, platform**<br>**Sequencing length, depth**<br>**Database used for taxonomic identification**	**Important findings**	**Comments**<br><br>**Strengths**<br><br>**Limitations and potential bias**
Tan *et al.*(1)<br><br>USA<br><br>2023	Multi-centre, prospective cohort study (2b)	270, 270<br>nr	RSV infection (270, 270)<br>nr<br><br>Nasal wash with sterile saline, nr<br>-80°C time nr<br><br>PowerSoil Kit (*Qiagen*)<br>515F, 806R<br>16S rRNA, V4, Miseq (*Illumina*)<br>300 bp, median 10,454, range 7,218-15,046 reads/sample<br>SILVA 138	**Composition**<br>• Most abundant genera at birth: *Moraxella* (39%), *Streptococcus* (27%), *Staphylococcus* (27%), *Haemophilus* (11%), *Corynebacterium* (5%), and *Dolosigranulum* (4%)<br><br>**RSV infection**<br>• Higher abundance of *Moraxella* in children with RSV-A compared to RSV-B	• -<br><br>• -<br><br>• Storage medium nr<br>• Clinical data only available for whole cohort, not for infants with microbiome analysis<br>• Overlap with participants from(2-4)
Hou *et al.*(5)<br><br>Hong Kong<br><br>2022	Single-centre, prospective cohort study (2b)	33, 144<br>nr	Healthy (9, 9)<br>Asthma (24, 130 during regular visits every 2-4w, 5 during exacerbation)<br>Mean 12y, SD 3y, range 6-17y<br><br>Nasopharyngeal swab, FLOQSwab (*Copan*), nr<br>On ice immediately, -80°C time nr<br><br>Power Soil DNA Isolation Kit (*Mo Bio*)<br>515F, 806R<br>16S rRNA, V4, HiSeq 2500<br>2x250, median 75,365, IQR 46,501-79,697 reads/sample<br>SILVA 123	**Composition**<br>• 30 phyla, 736 genera, 5209 ASVs (mean 99, range 10-476 ASV/sample)<br>• Firmicutes (35%), Proteobacteria (35%), Actinobacteria (29%), Bacteroidetes (0.7%), and Fusobacteria (0.3%)<br>• 6 profiles: *Moraxella-, Corynebacterium 1-, Dolosigranulum-, Staphylococcus-, Streptococcus-*, and *Anoxybacillus*-dominant<br>• *Moraxella-*, and *Dolosigranulum*-dominated profiles more stable composition over time<br>• *Dolosigranulum-* and *Corynebacterium 1*-dominated profile more frequent in baseline and healthy controls higher abundance of<br><br>**Asthma exacerbation**<br>• Lower diversity<br>• Higher abundance of *Moraxella* during exacerbation<br>• Metabolic pathways associated with *Moraxella* (methane, ketone bodies, and vitamin B3 metabolisms) enhanced during exacerbation<br>• None of the children with an exacerbation had a *Corynebacterium 1*-dominated profile	• 0% AB exposure 4w prior to enrolment (asthmatic children)<br>• 79% siblings (asthmatic children)<br>• 17% pets (asthmatic children)<br>• 21% tobacco smoke exposure (asthmatic children)<br>• Season of collection: 100% autumn/winter<br><br>• Longitudinal sample collection<br>• Inclusion of healthy controls<br><br>• Includes children across a wide age range<br>• Storage medium nr<br>• No clinical information for healthy controls<br>• No information on vaccination status<br>• Only includes a small number of children with asthma exacerbation
Kelly *et al.*(6)	Multi-centre, prospective	179, 1,368<br>45	Healthy (179, 1,368)<br>0, 1, 2, 3, 4, 5, 6, 8, 10, 12m	**Composition**	• 28% perinatally HIV exposed<br>• 100% vaginally born<br>• 85% breastfed

Botswana<br><br>2022	birth cohort study (2b)		Nasopharyngeal swab, MSwab (*Copan*)<br>-80°C within 4h<br><br>PowerSoil Pro Kit (*Qiagen*)<br>515F, 806R<br>16S rRNA, V4, MiSeq (*Illumina)*<br>2x250, >1,000 reads/sample<br>Human Oral Microbiome 15.1 and NCBI	• Most abundant genera at birth: *Staphylococcus* (22%), *Corynebacterium* (5%), *Lactobacillus* (4%)*, Gardnerella* (3%)*, Prevotella* (3%), and *Gemella* (3%)<br>• Most abundant genera 1 to 12m: *Corynebacterium* (20%), *Dolosigranulum* (14%), *Haemophilus* (9%)*, Moraxella* (30%), *Staphylococcus* (22%)*, Streptococcus* (10%)<br>• *S. pneumoniae* detected in 80% of infants, median at 2.4m<br>• *Moraxella*-dominated profile associated with more stable composition over time<br>• *Dolosigranulum-, Haemophilus-,* and *Streptococcus*-dominated profiles associated with less stable composition over time<br>• Negative association between abundance of *S. pneumoniae* and *Corynebacterium (C. pseudodiphtheriticum/propinquum, C. accolens/macginleyi, C. tuberculostearicum)*<br><br>**Age**<br>• Higher diversity at birth compared with later<br>• Increase in richness with increasing age<br>• At birth: higher abundance of *Acinetobacter, Gardnerella, Lactobacillus,* and *Sneathia*<br><br>**Breastfeeding**<br>• Higher abundance of *Corynebacterium*<br>• Lower abundance of *Haemophilus, Moraxella,* and *Streptococcus*<br><br>**Antibiotic exposure (amoxicillin, metronidazole, trimethoprim/sulfamethoxazole)**<br>• Lower abundance of *Corynebacterium, Lactobacillus*<br>• Higher abundance *of Haemophilus, Moraxella, Streptococcus*<br><br>**Season**<br>• Lower abundance of *Corynebacterium* during winter<br>• Higher abundance of *Haemophilus* during winter<br>• Higher abundance of *S. pneumoniae* during winter<br><br>**Household size**<br>• Abundance of *S. pneumoniae* positively associated with increasing number of children in the household<br><br>**PCV13**<br>• Lower abundance of *S. pneumoniae*<br><br>**No association between sex, urban residence, and microbiome composition**	• 85% ≥1dose of PCV13<br>• 64% exposed to AB (46% amoxicillin, 13% metronidazole, 24% trimethoprim/sulfamethoxazole)<br>• Median 1 sibling, IQR 1-3<br>• 40% living in rural area, 60% in urban area<br>• 37% enrolled in summer, 63% in winter<br><br>• Longitudinal sample collection<br>• Large sample number<br><br>• Sequencing depth nr<br>• No information on gestational age, day-care attendance, pets, tobacco smoke exposure
McCauley *et al.*(7)<br><br>USA<br><br>2022	Multi-centre, prospective cohort study (2b)	208, 278<br>nr	Exacerbation-prone asthma (208, 181 at baseline, 97 during ARTI)<br>Range 6 to 17y<br><br>Nasal wash with saline, M4RT transport media (*Thermo Fisher Scientific*)	**Age**<br>• Younger children higher abundance of *Moraxella, Haemophilus, Dolosigranulum; Ascochyta, Cladosporium*, and *Verticillium*<br>• Older children higher abundance of *Staphylococcus* and *Corynebacterium*	• Season of collection for samples taken during ARTI: 35% autumn, 34% winter, 23% spring, 8% summer<br><br>• Analysis of fungal composition<br><br>• Includes children with corticosteroid treatment

			Fridge <24h<br><br>Modified cetyltrimethylammonium bromide buffer<br>515F, 806R<br>16S rRNA, V4, NextSeq 500 (*Illumina)*<br>nr, nr<br>nr<br><br>Modified cetyltrimethylammonium bromide buffer<br>fITS7, ITS4<br>ITS2, MiSeq (*Illumina)*<br>nr, nr<br>UNITE	**Season**<br>• Higher abundance of *Staphylococcus* in autumn<br>• Higher abundance of *Moraxella* in spring<br>• No influence on fungal composition in healthy children, higher abundance of *Malassezia* in spring and *Candida and Cladosporium* in autumn in children with ARTI<br><br>**ARTI**<br>• Higher abundance of *Moraxella* and *Haemophilus* associated with presence of virus-positive ARTI<br>• Higher abundance of *Cladosporium* associated with longer duration from baseline to ARTI<br><br>**Asthma**<br>• Higher abundance of *Moraxella* and *Haemophilus* associated with exacerbation	• Includes children across a wide age range<br>• Sequencing length and depth nr<br>• Database used for bacterial taxonomic identification nr<br>• No information on sex, vaccination status, AB exposure, siblings, pets, tobacco smoke exposure
Aydin *et al.*(8)<br><br>Germany<br><br>2021	Multi-centre, prospective cohort study (2b)	64, 64<br>63	Healthy (18, 18, mean 8.1y, SD 3.4y, median 8 y), chronic wheezing with acute exacerbation (23, 23, mean 2.6y, SD 1.3y), asthma with acute exacerbation (23, 23, mean 9.4y, SD 3.5y)<br><br>Nasopharyngeal swab, ESwab (*Copan*)<br>-80°C immediately<br><br>QIAmp DNA Microbiome Kit (*Qiagen*)<br>nr<br>16S rRNA, V3-V4, MiSeq (*Illumina)*<br>2x300, >75,000 reads/sample<br>SILVA v132	**Atopy**<br>• Higher abundance of *Staphylococcaceae, Enterobacteriaceae, Burkholderiaceae, Xanthobacteraceae,* and *Sphingomonadaceae*<br><br>**Chronic wheezing**<br>• Higher abundance of Proteobacteria<br>• Higher abundance of *Burkholderiaceae, Enterobacteriaceae Sphingomonadaceae*, *Staphylococcaceae,* and *Xanthobacteraceae*<br>• Higher abundance of *Haemophilus* and *Moraxella*<br>• Higher abundance of *S. pneumoniae, M. catarrhalis, S. aureus,* and *H. influenzae*<br><br>**Asthma**<br>• Higher abundance of Firmicutes<br>• Higher abundance of *Staphylococcaceae*	• -<br><br>• Inclusion of healthy controls<br><br>• Small cohort<br>• Includes children with corticosteroid treatment<br>• Includes children across a wide age range<br>• No information on vaccination status, AB exposure, siblings, day-care attendance, pets, tobacco smoke exposure for children in whom16S rRNA sequencing was done
Binia *et al.*(9)<br><br>Bangladesh<br><br>2021	Single-centre, prospective cohort study (2b)	240, 422<br>48	Healthy (240, 422)<br>2, 4m<br><br>Nasopharyngeal swab, nr, nr<br>-20°C immediately, -80°C time nr<br><br>QIAamp MinElute Virus Spin Kit (*Qiagen)*<br>NEBNext Ultra II library Prep Kit (*New England Biolabs*)<br>Shotgun metagenomics, HiSeq (*Illumina*)<br>2x150, >10^7 reads/sample<br>NCBI RefSeq	**Composition**<br>• Most abundant species *S. pneumoniae, H. influenzae, M. catarrhalis, D. pigrum,* and *S. aureus*<br>• Positive correlation between abundance of *M. nonliquefaciens* and *S. pneumoniae, H. influenzae* and *M. catarrhalis*<br>• *Lactococcus lactis* subsp. *cremoris, C. acnes, M. osloensis, A. pittii, C. accolens, Streptococcus, S. hominis*, *Sphingomonas, S. epidermidis, S. aureus, A. viridans, S. haemolyticus, D. pigrum, S. arlettae* more abundant in infants not colonised with *S. pneumoniae, H. influenzae* or *M. catarrhalis*<br><br>**Age**<br>• Decrease in richness from 2 to 4m, no difference in diversity<br><br>**No association between delivery mode, breastfeeding, presence of fucosylated oligosaccharides in breast milk,[2] and prior infection and composition**	• 7% preterm born<br>• 74% vaginally born<br>• 100% breastfed (89% exclusively at 4m, 71% at 6m)<br>• 58% siblings<br>• Season of birth: pre-monsoon 33%, monsoon 43%, dry winter 24%<br><br>• Shotgun metagenomic analysis<br>• High sequencing depth<br>• Analysis at species level*<br><br>• Swabs and storage medium nr<br>• No information on vaccination status, AB exposure, day-care attendance, pets, tobacco smoke exposure

				**No association with composition and risk for ARTI**	
Coleman *et al.*(10)<br><br>Australia<br><br>2021	Community-based, cross-sectional study (4)	101, 101<br>48	Healthy (17), history of AOM (55), middle ear effusion (18), perforated tympanic membrane (7), AOM (4)<br>Mean 4.7, range 2-7y<br><br>Nasopharyngeal swab, FLOQSwab *(Copan)*<br>4°C <48h, then -80°C<br><br>Bead-beating with MagNA Pure *(Roche)*<br>341F/806R<br>16S rRNA, V3-V4, MiSeq (*Illumina)*<br>2x300, median 119,693, range 149-262,880 reads/sample<br>SILVA v138	**Composition**<br>• Mean 14 (range 1-73) genera/sample<br>• Positive association between abundance of *Dolosigranulum and Corynebacterium (D. pigrum* and *C. pseudodiphtheriticum)*<br>• Positive association between abundance of *Haemophilus* and *Streptococcus*<br>• Positive association between abundance of *Ornithobacterium* and *Helococcus, Dichelobacter, Cardiobacteriaceae*<br>• Positive association between *Dolosigranulum* and *Moraxella* and *Neisseriaceae* in children with history of AOM and perforated tympanic membrane<br>• In children without rhinorrhoea negative correlation between *Moraxella* and *Staphylococcus*<br><br>**Rhinorrhoea**<br>• No difference in diversity<br>• Lower abundance of *Staphylococcus (S. aureus)* and *Neisseriaceae*<br><br>**Prone to AOM**<br>• No difference in diversity<br>• Higher abundance of *Moraxella*<br>• Children with middle ear effusion higher abundance of *Ornithobacterium* compared with children who never had AOM<br><br>**No association between household size, season, and composition**	• 90% vaccinated with PCV13<br>• 0% AB exposure 3w prior to enrolment<br>• 57% live in remote, 43% in rural communities<br>• Mean 5 people/household<br>• 22% day-care attendance, 52% preschool, 18% school<br>• Season of collection: 39% autumn, 12% winter. 49% spring<br>• Inclusion of healthy controls<br>• Sufficient sequencing depth<br><br>• Cross-sectional study design<br>• Small cohort<br>• Storage medium nr<br>• Includes children across a wide age range<br>• No information on pets and tobacco smoke exposure<br><br>• 0% AB exposure 3w prior to enrolment
Chun *et al.*(11)<br><br>USA<br><br>2021	Single-centre, cross-sectional study (4)	132, 132<br>61	Asthma (132, 132)<br>Median 12.0y, IQR 5.0y<br><br>Nasopharyngeal swab, nr, nr<br>nr<br><br>DNeasy Mini Kit (*Qiagen*)<br>nr<br>16S rRNA, V3-V4, MiSeq (*Illumina)*<br>2x250, >2,000 reads/sample<br>SILVA v138	**Composition**<br>• Negative correlation between abundance of *Corynebacterium* and *S. epidermidis* and expression of genes involved in inflammatory processes<br><br>**Cat allergy**<br>• Lower diversity (Shannon index)<br>• Lower abundance of *Corynebacterium* and *S. epidermidis*<br><br>**Dog allergy**<br>• Lower abundance of *Corynebacterium*<br><br>**No association between bacterial composition and pollen allergy**	• 69% cat-sensitised, 73% dog-sensitised<br><br>• -<br><br>• Includes children with corticosteroid treatment<br>• Cross-sectional study design<br>• Includes children across a wide age range<br>• Swabs, storage medium and storage conditions nr<br>• Sequencing depth nr<br>• No information on vaccination status, AB exposure, pets, tobacco smoke exposure, season
Elling *et al.*(12)<br><br>USA<br><br>2021	Single-centre, cross-sectional study (4)	65, 65<br>68	Recurrent or chronic otitis media receiving tympanoplasty or ventilation tubes (65, 65)<br>Median 2y (range for whole cohort 9m-14.9y)<br><br>Nasopharyngeal swab, nr, nr<br>nr	**Prone to AOM**<br>• Higher abundance of *Cutibacterium* and a lower abundance of *Actinobacillus*, *Selenomonas* and *Saccharibacteria* in children with FUT2 variant (associated with increased risk for AOM)<br>• Higher abundance of *Cutibacterium, Escherichia-Shigella* and *Staphylococcus* and a lower abundance of	• 89% breastfed<br>• 14% tobacco smoke exposure<br>• 80% White, 9% Hispanic, 2% Asian, 9% other<br><br>• -

			MasterPure DNA Purification Kit (*Epicentre*)<br>16S rRNA, V1-V2, MiSeq (*Illumina)*<br>nr, median 115,176, IQR 46,275-170,300 reads/sample<br>SILVA 115	*Acintobacillus* in children with RASIP1 variant (associated with increased risk for AOM)	• Cross-sectional study design<br>• Small cohort<br>• Includes children across a wide age range<br>• Swabs, storage medium and storage conditions nr<br>• Sequencing length nr<br>• No information age range, delivery mode, gestational age, vaccination status, AB exposure, siblings, day-care attendance, pets
Folino *et al.*(13)<br><br>Italy<br><br>2021	Single-centre, cross-sectional study (4)	132, 132<br>69	Recurrent AOM (50, 50), recurrent AOM with tympanic membrane perforation (46, 46), atopy, allergic rhinitis, or recurrent wheezing (36, 36)<br>Median 3.5, range 2.1-4.7y<br><br>Nasopharyngeal swab, eNAT *(Copan)*<br>4°C <4h, then -80°C<br><br>nr<br>nr<br>16S rRNA, V3-V4, MiSeq (*Illumina)*<br>2x300, >5,000 (mean 135,485) reads/sample<br>RDP v16	**Composition**<br>• Most abundant genera *Moraxella* (42%)*, Streptococcus* (20%)*, Haemophilus* (11%)*, Dolosigranulum* (17%)*,* and *Corynebacterium* (9%)<br><br>**Prone to AOM**<br>• No difference in diversity<br>• Lower abundance of *Dolosigranulum* and *Corynebacterium*<br>• Abundance of *Dolosigranulum* and *Corynebacterium* even lower in children with recurrent AOM with tympanic membrane perforation<br>• Higher abundance of *Haemophilus* in children with recurrent AOM and recurrent AOM with tympanic membrane perforation<br>• Higher abundance of *Aelloiococcus* in children with recurrent AOM (not in children with recurrent AOM with tympanic membrane perforation)	• 83% vaginally born<br>• 81% breastfed<br>• 0% AB exposure or infection 4w prior to enrolment<br>• 61% older siblings<br>• 93% day-care attendance<br>• 28% tobacco smoke exposure<br>• Season of collection: 100% winter<br><br>• -<br><br>• Cross-sectional study design<br>• DNA extraction method nr<br>• Control children had atopy, allergic rhinitis, or recurrent wheezing<br>• No information on vaccination status and pets
Henares *et al.*(14)<br><br>Spain<br><br>2021	Single-centre, prospective case-control study (3b)	140, 140<br>52	Healthy (65, 65), invasive pneumococcal disease (27, 27), viral URTI (48, 48)<br>Median 3.3y, IQR 1.6-4.1y<br><br>Nasal aspirate, phosphate buffered saline<br>- 80°C time nr<br><br>MagNA Pure Compact Total Nucleic Acid Kit I (*Roche*)<br>16S rRNA, V3-V4, MiSeq (*Illumina*)<br>2x465, >30,000 reads/sample<br>RDP v2.12	**Composition**<br>• 602 OTUs<br><br>**Invasive pneumococcal disease**<br>• More frequently colonised with *S. pneumoniae*<br>• Lower abundance of *D. pigrum* and *M. lincolnii*<br><br>**Viral URTI**<br>• Lower diversity<br><br>**PCV vaccination**<br>• Higher diversity<br><br>**No association between age, sex, and breastfeeding duration and bacterial richness or diversity**	• 71% vaginally born<br>• 79% breastfed<br>• 70% ≥1dose of PCV<br>• 100% no AB > 24h prior<br>• 46% kindergarten attendance, 71% school<br>• 31% household members <5y<br>• 42% tobacco smoke exposure<br>• 68% White<br><br>• Inclusion of healthy controls<br>• Analysis at species level*<br><br>• Includes children across a wide age range<br>• Information on AB exposure only 24h prior
McCauley *et al.*(15)<br><br>USA<br><br>2021	Multi-centre, prospective cohort study (2b)	58, 354<br>57	Healthy (31, 141, Feb, April, Sep, Dec)<br>Children with URTI (27, 126 d3-4, 87 d10-15)<br>Range 4.0-6.6y<br><br>Nasopharyngeal swab, flocked swab, nr<br>DNAase/RNase-free cryovials with RNALater (*Ambion*)<br>4°C <24h, then -80°C<br><br>Bead beating plus AllPrep kit (*Qiagen*)<br>515F, 806R	**Composition**<br>• 524 OTUs<br>• 6 profiles: *Moraxella*-, *Dolosigranulum-, Haemophilus-Corynebacterium*-, *Staphylococcus*-, *Streptococcus*-dominated, and mixed<br>• Composition changed over time in almost all children<br><br>**Season**<br>• *Dolosigranulum/Corynebacterium*-dominated profile more frequent in autumn and summer	• 78% White or Caucasian, 10% Black or African, 3% American Asian, 2% American Indian/Alaska Native<br>• 7% Hispanic, 93% Non-Hispanic<br>• Season of collection: 28% autumn, 36% winter, 27% spring, 10% summer<br><br>• Inclusion of healthy controls<br><br>• Storage medium nr<br>• Sequencing length and depth nr

			16S rRNA, V4, NextSeq 500 (*Illumina*)<br>nr, nr<br>GreenGenes May 2013	• *Haemophilus*-dominated profile more frequent in spring and winter<br><br>**URTI/sinusitis**<br>• *Moraxella*-dominated profile associated with increased risk of URTIs and sinusitis<br>• *Haemophilus*-dominated profile associated with increased risk of URTIs but reduced detection of rhinovirus<br>• *Dolosigranulum/Corynebacterium*-dominated profile (also richer and more diverse) associated with less frequent URTI and sinusitis<br>• Children who developed URTI or sinusitis higher abundance of *Moraxella* and lower abundance of *Prevotella, Acetobacteraceae,* and *Chryseobacterium*<br><br>**Association between dominate genus, season, ethnicity, and composition**	• No information on vaccination status, AB exposure, siblings, day-care attendance, pets, tobacco smoke exposure
Raita *et al.*(16)<br><br>USA<br><br>2021	Multi-centre, cross-sectional study (4)	244, 244<br>60	Severe bronchiolitis with hospitalisation (244, 244 during hospitalisation)<br>Median 3m, IQR 2-6m<br><br>Nasal wash (*Medline Industries)*, nr<br>On ice immediately, then -80°C<br><br>Trizol LS reagent (*ThermoFisher Scientific*) with Direct-zol RNA Miniprep Kit (*Zymo Research*)<br>nr<br>Metatranscriptomics, NovaSeq6000 (*Illumina*)<br>2x250, >8x10^6 reads/sample<br>Expanded Human Oral Microbiome	**Antibiotic exposure**<br>• Higher abundance of *H. influenzae*<br><br>**Viral infection**<br>• Rhinovirus A infection associated with *Haemophilus*-dominant profile<br>• Rhinovirus C infection associated with *Moraxella*-dominant profile<br>• RSV infection associated with *S. pneumoniae*-dominated profile<br><br>**Asthma**<br>• Higher abundance of *H. influenzae* and *S. pneumoniae* associated with higher risk of developing asthma at the age of 5y<br>• High abundance of *M. nonliquefaciens* associated with lower risk of developing asthma at the age of 5y	• 14% maternal smoking during pregnancy<br>• 19% preterm born<br>• 65% vaginally born<br>• 32% AB exposure<br>• 29% day-care attendance<br>• 14% tobacco smoke exposure<br>• 65% infected with RSV, 5% with rhinovirus, 12% with RSV and rhinovirus, 14% with RSV and another pathogen<br>• 25% asthma at age 5 years<br>• 42% Non-Hispanic White, 23% non-Hispanic Black, 31% Hispanic, 4% other<br><br>• Large cohort<br>• High sequencing depth<br>• Analysis at species level*<br><br>• Cross-sectional study design<br>• Storage medium nr<br>• No information on breastfeeding, vaccination status, siblings<br>• Overlap of participants with(17-26)
Reyman *et al.*(27)<br><br>Netherlands<br><br>2021	Single-centre, prospective birth cohort study (2b)	112, 430<br>nr	Healthy (112, 430)<br>1w, 2, 4, 6m<br><br>Nasopharyngeal swab, ESwab (*Copan*)<br>Frozen immediately, then -80°C<br><br>Modified Mag Forensics Extraction Kit protocol (*LGC Genomics*)<br>nr<br>16S rRNA, V4, MiSeq (*Illumina*)	**Composition**<br>• 895 OTUs<br>• Composition over time less stable than in the oropharynx or intestine<br>• Factors than influenced composition most were age, pets, mode of delivery, duration of hospital stay after delivery, number of siblings < 5y of age, breastfeeding, season of enrolment, ABs in 30d before sampling, pacifier use, and day-care attendance<br><br>**Age**	• 0% preterm born<br>• 63% vaginally born<br>• 31% AB exposure<br>• 59% siblings <5y of age<br>• 70% day-care attendance<br>• 3% tobacco smoke exposure<br><br>• Longitudinal sample collection<br>• Analysis at species level*

			2x240-260, 3,000 reads/sample<br>SILVA v119	• Decrease of *S. aureus* over time, increase of *C. propinquum*, *Dolosigranulum, Moraxella* and *Haemophilus*	• Low sequencing depth<br>• No information on breastfeeding, vaccination status, pets<br>• Overlap of participants with(28-30)
Tang *et al.*(31)<br><br>USA<br><br>2021	Single-centre, prospective birth cohort study (2b)	289, 2,922<br>55	Birth cohort, health status nr (289, 1,059 during health at 2, 4, 6, 9, 12, 18, 24m, 1,863 during ARTI)<br><br>Nasopharyngeal swab or aspirate, nr, nr<br>nr<br><br>Wizard SV Genomic DNA System (*Promega*)<br>515F, 806R<br>16S rRNA, V4, MiSeq (*Illumina*)<br>253 bp, nr<br>GreenGenes 13_5	**Composition**<br>• Most abundant genera *Dolosigranulum, Corynebacterium, Haemophilus, Moraxella, Streptococcus,* and *Staphylococcus*<br>• Most abundant species *D. pigrum, C. pseudodipththeriticum, H. influenzae, M. catarrhalis, S. pneumoniae, S. aureus, S. epidermidis,* and *S. mitis*<br><br>**Age**<br>• 4 profiles in first 6m: *Dolosigranulum/Corynebacterium-, Moraxella-, Staphylococcus-, Streptococcus*-dominated<br>• At 24m all children had mixed composition (many dominated by *Moraxella)*<br><br>**ARTI**<br>• More often *H. influenzae-, M. catarrhalis-, and S. pneumoniae-*dominated profiles, less frequently *D. pigrum-, C. pseudodipththeriticum-, S. mitis- and Staphylococcus*-dominated profiles<br><br>**Acute wheezing**<br>• More often *S. pneumoniae*-dominated profile, less frequently *D. pigrum*-dominated profile	• 87% vaginally born<br>• 31% exclusively breastfed first 6m<br>• 55% siblings<br>• 46% day-care attendance<br>• 35% dog at home at birth, 29% cat<br><br>• Longitudinal sample collection<br>• Large sample number<br>• Analysis at species level*<br><br>• Swab, medium and storage condition nr<br>• Sequencing depth nr<br>• No information on gestational age, vaccination status, AB exposure
Tozzi *et al.*(32)<br><br>Italy<br><br>2021	Single-centre, cross-sectional study (4)	54, 54<br>57	Symptoms compatible with pertussis (54, 54 during hospitalisation)<br><12m, median 1.5m, IQR 1-3m<br><br>Nasopharyngeal aspirate, nr<br>-80°C immediately<br><br>EZ1 DNA tissue kit (*Qiagen*)<br>nr<br>16S rRNA, V3-V4, MiSeq (*Illumina*)<br>2x300, mean 44,328 reads/sample<br>GreenGenes 13_08	**Composition**<br>• 15 phyla, 95 families, 245 OUTs<br><br>**Pertussis infection**<br>• Higher abundance of Alcaligenaceae and *Achromobacter*<br><br>**Rhinovirus infection**<br>• Higher abundance of Moraxellaceae and *Moraxella*	• 19% preterm born<br>• 51% vaginally born<br>• 43% exclusively breastfed<br>• 25% AB exposure prior to sampling<br><br>• -<br><br>• Cross-sectional study design<br>• Small cohort<br>• Storage medium nr<br>• No information on vaccination status, siblings, day-care attendance, pets, tobacco smoke exposure
Xu *et al.*(33, 34)<br><br>USA<br><br>2021	Multi-centre, prospective cohort study (2b)	96, 157<br>nr	Children without AOM (68)<br>Otitis-prone children (28)<br>6m (74), 12m (85)<br><br>Nasal wash, virus transfer media<br>-80°C timing nr<br><br>nr<br>515F, 806R<br>16S rRNA, V4, MiSeq (Illumina)<br>nr, nr<br>SILVA	**Composition**<br>• Infants colonised with *S. pneumoniae* had a lower abundance of Actinobacteria and *Corynebacteriaceae* at 6 and 12m, a lower abundance of *Carnobactericeae* at 12m and a higher abundance of *Moraxellaceae* at 6m<br>• Infants colonised with *S. pneumoniae* had a lower abundance of *Actinomyces, Prevotella, Dolosigranulum, Veillonella, Corynebacterium_1, Gemella* and *Anoxybacillus* at 6m and a lower abundance of *Corynebacterium* and a higher abundance of *Streptococcus* at 12m	• 100% vaccinated with either PCV7 or PCV13<br><br>• Analysis at species level*<br><br>• Small cohort<br>• DNA extraction method nr<br>• Sequencing depth and length nr<br>• Unclear which infants were included both at 6 and 12m<br>• No information on delivery mode, gestational age, breastfeeding, AB exposure, siblings, day-care attendance, pets, tobacco smoke exposure

				• Infants colonised with *S. pneumoniae* but not *M. catarrhalis* had a lower abundance of *Corynebacterium_1* and *Actinomyces* and a higher abundance of *Alloprevotella* at 6m<br>• No difference in diversity (Shannon, Simpson, and inverted Simpson index) in infants colonised or not colonised with *S. pneumoniae* at 6 and 12m<br><br>**Day-care attendance**<br>• Infants who attended day-care attendance were more frequently colonised with *S. pneumoniae*<br>• Less genera remained differentially abundant between infants colonised with S. pneumoniae and these not colonised when adjusted for day-care attendance<br><br>**URTI**<br>• Infants with URTI colonised with *S. pneumoniae* had a higher abundance of *Haemophilus* and *Streptococcus*<br>• Infants without URTI colonised with *S. pneumoniae* had a higher abundance of *Streptococcus* and a lower relative abundance of *Corynebacterium_1* at 12m<br><br>**Recurrent AOM**<br>• Infants prone to AOM had a lower diversity (Shannon, Simpson, and inverted Simpson index) at 6m but not 12m<br>• Infants prone to AOM had a higher abundance of Proteobacteria at 6m and a higher abundance of Firmicutes at 12m, a lower abundance *Fusobacteriaceae, Prevotellaceae* and *Veillonellaceae* and a higher abundance of *Moraxellaceae* at 6m and a higher abundance of *Carnobacteriaceae* at 12m, a lower abundance of *Fusobacterium, Prevotella* and *Veillonella* and a higher abundance of *Moraxella* at 6m and lower abundance *Bacillus, Veillonella, Gemella* and *Prevotella* and a higher abundance of *Dolosigranulum* at 12m<br>• Infants without AOM colonised with *S. pneumoniae* had a lower abundance of *Corynebactierum_1, Dolosigranulum* and *Actinomyces* and a higher abundance of *Bacillus* than infants without AOM not colonised with *S. pneumoniae*	
Accorsi *et al.*(35)<br><br>Israel<br><br>2020	Single-centre, prospective birth cohort study (2b)	33, 144<br>45	Healthy (33, 144)<br><2d, monthly until 12m<br><br>Nasopharyngeal swab, cotton swab, Amies transport medium (*Copan*)<br>nr<br><br>Qiagen DNeasy PowerLyzer PowerSoil Kit (*Qiagen*)<br>Nextera XT DNA library preparation kit (*Illumina*)<br>Shotgun metagenomics, HiSeq (*Illumina*)<br>2x101, <5x10^4, mean 9.47x10^5 reads/sample<br>MetaPhlAn2 v2.6.0	**Composition**<br>• Increase in stability of species composition with increasing age<br>• Positive correlation between the abundance of *Acinetobacter, S. parasanguinis, S. salivarius* and *Veillonella* and acquisition of *S. aureus* and negative correlation b between the abundance *D. pigrum* and acquisition of *S. aureus*<br><br>**Day-care attendance**<br>• Increase in abundance of *M. catarrhalis* and *H. influenzae*	• 80% vaginally born (data not available for 8)<br><br>• Longitudinal sample collection<br>• Shotgun metagenomics<br>• Analysis at species level*<br><br>• Small cohort<br>• Storage conditions nr<br>• No information on gestational age, breastfeeding, vaccination status, AB exposure, siblings, day-care attendance, pets, tobacco smoke exposure

Chapman *et al.*(36)<br><br>USA<br><br>2020	Single-centre, retrospective cohort study (analysis of data from a prospective cohort study) (3b)	34, 34<br>nr	Healthy (20, 20), prone to infection and allergy (14, 14)<br>6m<br><br>Nasal wash, phosphate buffered saline<br>-80°C immediately<br><br>nr<br>515F, 806R<br>16S rRNA, V4, MiSeq (*Illumina*)<br>nr, >1,000/sample<br>GreenGenes 13_08	**Composition**<br>• 29 genera with >1% abundance<br>• Most abundant genera *Moraxella, Alloicoccus, Streptococcus, Corynebacterium, Veillonella,* and *Prevotella*<br>• Relative abundance of *M. catarrhalis* negatively associated with bacterial diversity<br>• Colonisation with a pathogen had no impact on abundance of commensals<br><br>**Infection and allergy prone infants**<br>• Higher abundance of *M. catarrhalis*<br>• Trend towards lower diversity	• -<br><br>• Inclusion of healthy controls<br>• Analysis at species level*<br><br>• Small cohort<br>• DNA extraction method nr<br>• Sequencing length nr<br>• No information on delivery mode, gestational age, breastfeeding, vaccination status, AB exposure, siblings, day-care attendance, pets, tobacco smoke exposure
Enoksson *et al.*(37)<br><br>Sweden<br><br>2020	Single-centre, prospective cohort study (2b)	27, 20<br>52	Otitis media with effusion (27, 20)<br>Median 4.8y<br><br>Nasopharyngeal swab, ESwab (*Copan*)<br>-80°C immediately<br><br>Modified Mag Forensics Extraction Kit Protocol (L*GC Genomics*)<br>nr<br>16S rRNA, V4, MiSeq (*Illumina*)<br>nr, nr<br>SILVA v119	**Composition**<br>• Most abundant genera *Moraxella, Corynebacterium, Dolosigranulum, Haemophilus,* and *Streptococcus*<br>• Presence of *Haemophilus* and *C. propinquum* associated with anti-inflammatory mediators<br>• Presence of *Turicella* and *Dolosigranulum* associated with pro-inflammatory mediators	• -<br><br>• -<br><br>• Small cohort<br>• Sequencing length and depth nr<br>• Age range nr<br>• No information on vaccination status, AB exposure, siblings, day-care attendance, pets, tobacco smoke exposure
Haro *et al.*(38)<br><br>Japan<br><br>2020	Single-centre, prospective cohort study (2b)	40, 40<br>55	Rhinorrhoea (40, 40)<br>2.1y (range 0.2-7y)<br><br>Aspiration of nasal discharge (*AS ONE Corporation*), sterile container (*Nippon Covidien Ltd*)<br>4°C <7d<br><br>*In-house* DNA extraction protocol<br>E341F, E907R<br>16S rRNA, V3-V5, 3130xl Genetic Analyzer (*Applied Biosystems*)<br><500 bp, mean 163 reads/sample<br>RDP	**Composition**<br>• 69 OTUs, *Haemophilus* (40%)*, Moraxella* (31%)*, Streptococcus (15%)* highest abundance, followed by *Pseudomonas* (4%), *Novosphingobium* (3%)*, Corynebacterium* (2%)*, Dolosigranulum* (1%)<br>• 6 profiles: mixed, *M. catarrhalis/nonliquefaciens*-dominated*, H. aegyptius/influenzae* (OTU3)-dominated*, S. pneumoniae*-dominated*, H. aegyptius/influenzae* (OTU4)-dominated, *H. aegyptius/influenzae* (OTU0)-dominated	• 60% vaccinated against Hib and PCV<br>• 33% AB 4 weeks prior<br>• 58% day-care attendance<br><br>• Analysis at species level*<br><br>• Small cohort<br>• Samples not kept frozen<br>• Includes children across a wide age range<br>• Low sequencing depth<br>• No information on siblings, pets, tobacco smoke exposure
Liu *et al.*(39)<br><br>China<br><br>2020	Single-centre, cross-sectional study (4)	155, 155<br>59	Healthy (155, 155)<br>Mean 5.5y, SD 0.5y, range 5-6y<br><br>Nasal wash with sterile saline, nr<br>nr<br><br>QIAamp DNA Mini Kit *(Qiagen)*<br>341F, 805R<br>16S rRNA, V3-V4, HiSeq 2500 (*Illumina*)<br>nr, 12,773-464,276 reads/sample (includes adult samples)	**Composition**<br>• Compared with adults, children had higher diversity and a higher abundance of Proteobacteria and *D. pigrum*<br>• Moraxella only present in children<br>• Compared with adults, children had a lower abundance of *Staphylococcus*<br><br>**No association between sex and composition**	• -<br><br>• High sequencing depth<br>• Analysis at species level*<br><br>• Cross-sectional study design<br>• Storage medium and conditions nr<br>• Sequencing length nr<br>• No information on delivery mode, gestational age, breastfeeding, vaccination status, AB exposure, siblings, pets, tobacco smoke exposure

			SILVA		
Man *et al.*(40)<br><br>Netherlands<br><br>2020	Multi-centre, single-blinded, randomised, placebo-controlled trial (1b)	145, 145<br>59	Infants born preterm (32-35w) receiving palivizumab or placebo (145, 145)<br>1y<br><br>Nasopharyngeal swab, nr, nr<br>nr<br><br>Modified Mag Forensics Extraction Kit protocol (*LGC Genomics*)<br>533F, 806R<br>16S rRNA, V4, MiSeq (*Illumina*)<br>nr, > 9,000 reads/sample (mean 36,412)<br>SILVA v119	**Composition**<br>• Most abundant genera or species *E. faecium, M. osloensis, Chryseobacterium, Rothia, Brevundimonas* and *S. salivarius*<br><br>**Palivizumab**<br>• Infants who received palivizumab higher frequency of *Staphylococcus*-dominated profiles, higher abundance of biomarker species, such as *Klebsiella*, and more diverse set of oral taxa, including *Streptococcus* at 1y<br><br>**RSV**<br>• Infants without RSV infection until 1y of age had a lower abundance of *Moraxella* and *Neisseriaceae* at 1y	• 18% maternal smoking during pregnancy<br>• 64% breastfed<br>• 46% pets<br>• 39% tobacco smoke exposure<br><br>• -<br><br>• Swab, storage medium and condition nr<br>• DNA extraction method nr<br>• Sequencing length nr<br>• No information on delivery mode, gestational age, vaccination status, day-care attendance, siblings
		342, 342<br>56	Infants born preterm (32-35w) receiving palivizumab or placebo (342, 342)<br>6y<br><br>Nasopharyngeal swab, nr, nr<br>nr<br><br>Modified Mag Forensics Extraction Kit protocol (*LGC Genomics*)<br>533F/806R<br>16S rRNA, V4, MiSeq (*Illumina*)<br>nr, > 9,000 reads/sample (mean 36,412)<br>SILVA v119	**Composition**<br>• Most abundant genera or species *Haemophilus, Moraxella, S. pyogenes, Corynebacterium,* and *Dolosigranulum*<br><br>**Palivizumab**<br>• Children who received palivizumab higher abundance of *Haemophilus* and lower abundance of *Moraxella* and *Neisseriaceae* at 6y<br><br>**RSV**<br>• Children without RSV at 1y had a higher abundance of *Haemophilus* at 6y<br><br>**Reversible airway obstruction**<br>• Children with reversible airway obstruction at 6y had a higher abundance of *Haemophilus* and *S. pneumoniae* and lower abundance of *Moraxella, Corynebacterium, Dolosigranulum*, and *Staphylococcus* at 6y	• 14% maternal smoking during pregnancy<br>• 70% breastfed<br>• 46% pets<br>• 35% tobacco smoke exposure<br><br>• Large cohort<br><br>• Swab, storage medium and condition nr<br>• Sequencing length nr<br>• No information on delivery mode, gestational age, vaccination status, day-care attendance, siblings
Mansbach *et al.*(20)<br><br>USA<br><br>2020	Multi-centre, prospective cohort study (2b)	842, 2,086<br>60	Bronchiolitis with hospitalisation (842, 2,086 During hospitalisation, 3w after hospitalisation, during summer, 1y after hospitalisation)<br>Median 3.2m, IQR 1.7-5.8m<br><br>Nasopharyngeal wash with normal saline, nr (*Medline Industries*)<br>4°C within 1h, -80°C within 24h<br><br>PowerSoil DNA Isolation Kit (*Mo Bio*)<br>nr<br>16S rRNA, V4, MiSeq (*Illumina*)<br>2x250, >1,000 reads/sample<br>SILVA	**Recurrent wheezing**<br>• Infants with a higher abundance of *Moraxella* or *Streptococcus* 3w after hospitalisation and a higher abundance of *Streptococcus* in summer had a higher risk for recurrent wheezing at 3y	• 14% maternal smoking during pregnancy<br>• 67% vaginally born<br>• 45% breastfed<br>• 31% prior AB, time nr<br>• 23% day-care attendance<br>• 15% tobacco smoke exposure<br><br>• Longitudinal sample collection<br>• Large cohort<br>• Large sample number<br><br>• Low sequencing depth<br>• Storage medium nr<br>• No information on gestational age, vaccination status, siblings, pets

					• Overlap of participants with(16-19, 21-26)
Salgado *et al.*(41)<br><br>Brazil<br><br>2020	Single-centre, cross-sectional study (4)	84, 84<br>nr	ARTI (84, 84)<br>6-23m<br><br>Nasopharyngeal aspirate, sterile tube with NucliSENS lysis buffer (Biomerieux)<br>nr<br><br>nr<br>nr<br>16S rRNA, V3-V4, MiSeq (Illumina)<br>300 bp, sequencing depth nr<br>SILVA v123	**Composition**<br>• Genera with the highest abundance were *Streptococcus, Haemophilus,* and *Moraxella*<br><br>**Vaccination**<br>• Children vaccinated with Hib/PCV10 had a higher richness but similar diversity<br>• No difference in the abundance of different bacteria between children vaccinated with Hib/PCV10 and non-vaccinated children	• 57% vaccinated with PCV10<br><br>• -<br><br>• Cross-sectional study design<br>• Small cohort<br>• Storage conditions nr<br>• DNA extraction method nr<br>• Sequencing depth nr<br>• No information on delivery mode, gestational age, breastfeeding, vaccination status, AB exposure, siblings, day-care attendance, pets, tobacco smoke exposure
Shilts *et al.*(42)<br><br>USA<br><br>2020	Single-centre, cross-sectional study (4)	20, 40<br>40	Healthy (20, 40)<br>Mean 3.1m, SD 0.1m<br><br>Nasal filter (Leukosorb, Pall Life Sciences)<br>Nasal wash with normal saline, nr<br>-80°C time nr<br><br>PowerSoil DNA Isolation Kit (*Qiagen*)<br>16S rRNA, V4, MiSeq (Illumina)<br>2x250 bp, >1,000 reads/sample, median 15,858 (IQR 4777-21,149) reads/sample<br>RDP<br><br>Shotgun metagenomics (n=8)<br>Nextera XT DNA Library Prep Kit (*Illumina*)<br>NovaSeq6000 (*Illumina*)<br>2x150, median 738,821 (IQR 538,420-1,855,491)<br>GOTTCHA	**Composition**<br>• 81 genera, 60 species<br>• Nasal filters: most abundant genera *Prevotella* (29%), *Veillonella* (14%), *Streptococcus* (10%), *Moraxella* (10%), *Haemophilus* (5%), *Neisseria* (4%), *Actinomyces* (3%)<br>• Nasal washes: most abundant genera *Moraxella* (21%), *Prevotella* (20%), *Veillonella* (12%), *Haemophilus* (11%), *Streptococcus* (6%), *Dolosigranulum* (4%), *Neisseria* (3%), *Corynebacterium* (3%)<br>• The only genus differently abundant between nasal filters and washes was *Sphingobium*<br>• Nasal filters: most abundant species *M. catarrhalis* (37%), *H. influenzae* (21%), *S. pneumoniae* (14%), *R. pickettii* (13%) and *P. acnes* (9%)<br>• Nasal washes: most abundant species *M. catarrhalis* (35%), *S. pneumoniae* (19%) *H. influenzae* (16%), *R. pickettii* (8%), and *P. acnes* (8%)	• -<br><br>• Shotgun metagenomics<br>• Analysis at species level*<br><br>• Cross-sectional study design<br>• Small cohort<br>• Storage medium nr<br>• No information on delivery mode, gestational age, breastfeeding, vaccination status, AB exposure, siblings, day-care attendance, pets, tobacco smoke exposure
Thapa *et al.*(43)<br><br>USA<br><br>2020	Single-centre, cross sectional study (4)	49, 49<br>57	Receiving tympanostomy tubes (34, 34), tonsillectomy and adenoidectomy (15, 15)<br>Mean 3y, SD 1.8y, range 1-6y<br><br>Nasopharyngeal swab, FLOQSwab (*Copan*), sterile empty tube<br>-80°C immediately<br><br>MoBio Power Soil kit (*Qiagen*)<br>nr<br>16S rRNA, V4, MiSeq (*Illumina*)<br>2x250, >100 reads/sample<br>SILVA v123	**Composition**<br>• 27 OTUs<br>• Most abundant genera *Haemophilus, Veillonella, Corynebacterium, Dolosigranulum, Prevotella,* and *Fusobacterium*<br><br>**Antibiotics prior 3m**<br>• Higher abundance of *Haemophilus*<br><br>**No association between sex, delivery mode, feeding method, and probiotic and composition**	• 60% vaginally born<br>• 65% exclusively breastfed<br>• 76% AB exposure prior 3m<br>• 16% probiotic intake<br><br>• -<br><br>• Small cohort<br>• Includes children across a wide age range<br>• Low sequencing depth<br>• No information on vaccination status, siblings, day-care attendance, pets, tobacco smoke exposure
Verhagen *et al.*(44)	Multi-centre, cross	191, 191<br>46	Healthy (60, 60) ARTI (103, 103), gastrointestinal infection (28, 28)<br>Median 15m, IQR 7-21m, maximum 5y	**Composition**<br>• 121 OTUs	• 79% breastfed<br>• 8% AB exposure prior 1w

Venezuela<br><br>2020	sectional study (4)		Nasopharyngeal swab, swab (*Copan*), STGG<br>4°C ≤3d, -20°C < 4w, -70°C<br><br>Phenol/bead-beating and magnetic bead separation (*LGC Genomics)*<br>nr<br>16S rRNA, V4, MiSeq (*Illumina*)<br>2x250, median 10,375, range 107-74,630 read/sample<br>SILVA v119	• Most abundant genera *Moraxella, Corynebacterium, Dolosigranulum, Haemophilus,* and *Streptococcus*<br><br>**Respiratory tract infection**<br>• Higher abundance of *Klebsiella, Anoxybacillus, Bacillus, Thermus thermophilus, Arthroabcter, Xanthobacteraceae, Actinomyces* and lower abundance of *Caulobacteraceae, Schlegelella, Pseudomonas, P. putida, P. syringae, Corynebacterium, C. propinquum, Acinetobacter, A. soli, S. maltophilia, Wautersiella*<br><br>**Gastrointestinal infection**<br>• Lower diversity<br>• More often had a *Klebsiella*- and *Leuconostoc*-dominated clusters<br>• Higher abundance of *Klebsiella, Leuconostoc, L. algidus, Vagococcus, Staphylococcaceae, Pedoabcter, T. thermophilus, Anoxybacillus, Shewanella, Flavobacterium, Myroides* and lower abundance of *Acinetobacter, Aerococcus, A. soli, Wautersiella, Moraxella, Haemophilus, C. propinquum, Bergeyella, Psychrobacter, Cloacibacterium, Helococcus, Schlegelella, A. porcinus, Moraxella, Lsyobacter, S. maltophilia, Rothia, Veillonella, Alloprevotella*<br><br>**Age**<br>• Richness lower in younger children	• Large cohort<br><br>• Cross-sectional study design<br>• Includes children across a wide age range<br>• Includes samples with low sequencing depth<br>• No information on delivery mode, vaccination status, siblings, day-care attendance, pets, tobacco smoke exposure
Zhou *et al.*(45)<br><br>China<br><br>2020	Single-centre, prospective cohort study (2b)	165, 165<br>nr	Healthy (59, 59), *M. pneumoniae* pneumonia (40, 40), influenza infection (66, 66)<br>nr<br><br>Nasopharyngeal swab, 25-800-A-50 (*Puritan*)<br>-80°C within 10min<br><br>Power Soil DNA Isolation Kit (*Mo Bio*)<br>nr<br>16S rRNA, V3-V4, MiSeq (*Illumina*)<br>nr, nr<br>Live Tree Project	**Influenza infection**<br>• Increase in diversity<br>• 5 profiles: *Moraxella-, Streptococcus-, Staphylococcus-, Corynebacterium-, Dolosigranulum*-dominant<br>• Higher abundance of *Phyllobacterium compared with healthy children and children with M. pneumoniae* pneumonia<br>• Higher abundance of *Ralastonia and* unclassified_*Acidobacteria* compared with healthy children<br><br>***M. pneumoniae* pneumonia**<br>• Decrease in diversity<br>• *Staphylococcus*-dominated profile more frequent<br>• Higher abundance of *Ralastonia and* unclassified_*Acidobacteria* compared with healthy children<br><br>**No association between age, sex, delivery mode, and feeding method and composition**	• 0% AB 4w prior to enrolment<br><br>• Inclusion of healthy controls<br><br>• Sequencing length and depth nr<br>• No information on age, vaccination status, siblings, day-care attendance, pets, tobacco smoke exposure<br>• Overlap with participants from(46-48)
Boelsen *et al.*(49)<br><br>Fiji<br><br>2019	Multi-centre, retrospective cohort study (participants from a single-blinded, open-label phase II	132, 132<br>54	Healthy Indigenous infants (42, 42), Indigenous infants with URTI (25, 25), healthy infants of Indian descent (51, 51), infants of Indian descent with URTI (14, 14)<br>12m<br><br>Nasopharyngeal swab, aluminium shaft-buffered cotton swabs (*Sarstedt*), STGG	**Composition**<br>• 4036 OTUs (847 OTUs with unique taxonomic names)<br>• Most abundant genera *Dolosigranulum, Pseudomonas, Corynebacterium, Moraxella, Haemophilus, Streptococcus* and *Staphylococcus*<br>• Positive association between abundance of *Streptococcus* and *Moraxella* and *Streptococcus* and *Haemophilus*	• 64% breastfed<br>• 50% vaccinated with 3 doses of PCV7<br>• 10% AB exposure 2w prior<br>• 46% tobacco smoke exposure<br><br>• Inclusion of healthy controls

	vaccine trial) (3b)		-70°C time nr<br><br>*QIAamp* DNA mini kit (*Qiagen*)<br>515F, 806R<br>16S rRNA, V4, MiSeq (*Illumina*)<br>250 bp, >50,000/sample<br>MiSeq SOP and SILVA v119	• Negative association between abundance of *Streptococcus* and *Pseudomonas*<br>• No association between abundance of *Streptococcus* and *Dolosigranulum*<br><br>**Ethnicity**<br>• Infants of Indian descent had a higher diversity compared with Indigenous infants, no influence on richness<br>• Indigenous infants higher abundance of *Moraxella*, *Haemophilus* and *Helcococcus* and lower abundance of *Staphylococcus, Dolosigranulum* and *Corynebacterium* compared with infants of Indian descent<br><br>**URTI**<br>• Higher abundance of *Moraxella, Haemophilus, Streptococcus,* and lower abundance of *Dolosigranulum* and *Corynebacterium*<br>• Composition of Indigenous Fijian infants without URTI similar to composition of Fijian infants of Indian descent with URTI symptoms<br><br>**PCV7**<br>• No change in diversity, richness, or overall composition<br>• Lower abundance of *S. pneumoniae*<br><br>**No association between sex, breastfeeding, AB exposure (drug ns) in 2w prior, season, and tobacco smoke exposure, and composition**	• Differences in vaccination status<br>• No information on delivery mode, gestational age, siblings, day-care attendance, pets
Man *et al.*(50)<br><br>Netherlands<br><br>2019	Single-centre, cross-sectional study (4)	29, 29<br>52	Children admitted to ICU with LRTI (29, 29)<br>Median 2.2m, IQR 1.6-3.6m<br><br>Nasopharyngeal swab, nr, nr<br>-20°C immediately, -80°C time nr<br><br>Modified Mag Forensics Extraction Kit protocol (*LGC Genomics*)<br>533F, 806R<br>16S rRNA, V4, MiSeq (*Illumina*)<br>nr, >15,000 reads/sample<br>SILVA v119	**Composition**<br>• Most abundant taxa *M. catarrhalis/nonliquefaciens-, H. influenzae/haemolyticus,* and *S. pneumoniae*<br><br>**No association between AB before sampling or bacterial LRTI and composition**	• 67% vaginally born<br>• 37% currently breastfed and/or breastfed >3m<br>• 13% AB 6m prior<br>• 17% AB before sampling<br>• 55% tobacco smoke exposure<br><br>• Analysis at species level*<br><br>• Cross-sectional study design<br>• Small cohort<br>• Swab and storage medium nr<br>• Sequencing length nr<br>• No information on vaccine status
	Multi-centre, prospective, matched case-control study (3b)	461, 457<br>60	Healthy (307, 307), children admitted to ICU with LRTI (154, 154)<br>Median 13.6m, IQR 4.9-27.4m<br><br>Nasopharyngeal swab, nr, nr<br>-20°C immediately, -80°C time nr<br><br>Modified Mag Forensics Extraction Kit protocol (*LGC Genomics*)<br>533F, 806R	**Composition**<br>• 7 profiles: *S. aureus/epidermidis-, C. macginleyi/accolens-, H. influenzae/haemolyticus-, M. catarrhalis/nonliquefaciens-, V. dispar and A. porcinus-, S. pneumoniae-, C. propinquum and D pigrum*-dominated<br><br>**LRTI**<br>• *H. influenzae/haemolyticus*- and *S. pneumoniae*-dominated profiles more frequent	• 5% preterm born<br>• 83% vaginally born<br>• 13% prior 6m<br>• 17% tobacco smoke exposure<br><br>• Large cohort<br>• Inclusion of healthy controls<br>• Analysis at species level*<br><br>• Swab and storage medium nr

			16S rRNA, V4, MiSeq (*Illumina*)<br>nr, >15,000 reads/sample<br>SILVA v119	• *M. catarrhalis/nonliquefaciens*- and *C. propinquum and D pigrum*-dominated profiles less frequent<br>• Higher abundance of *H. influenzae/haemolyticus, S. pneumoniae, Actinomyces, Prevotella*<br>• Lower abundance of *Moraxella, C. propinquum, D. pigrum, Helococcus*<br><br>**Association between season, age, day-care attendance, breastfeeding, history of previous ARTI, and AB prior 6m and composition**<br><br>**No association between sex and composition**	• Sequencing length nr<br>• No information on vaccine status
Man *et al.*(51)<br><br>UK<br><br>2019	Single-centre, cross-sectional study (4)	94, 94<br>61	Children with AOM with tympanostomy tubes: AOM (32, 32), otitis media with effusion (43, 43), both (19, 19)<br>Mean 3.4y, SD 1.4y<br><br>Nasopharyngeal swab, nr, nr<br>Frozen immediately, then -80°C<br><br>Modified Mag Forensics Extraction Kit protocol (*LGC Genomics*)<br>533F, 806R<br>16S rRNA, V4, MiSeq (*Illumina*)<br>2x240-260, >10,000, mean 43,147 reads/sample<br>SILVA v119	**Composition**<br>• 8 phyla, 66 genera, 138 OTUs<br><br>**Otorrhoea**<br>• Abundance of *Acinetobacter, Klebsiella, Neisseria, Haemophilus* associated with longer duration of otorrhoea, abundance of *Corynebacterium, Dolosigranulum, Haemophilus* associated with shorter duration<br><br>**Correlation between bacteria found in nasopharyngeal swabs and middle ear fluid, especially for *Streptococcus, Corynebacterium, Klebsiella,* and *Haemophilus***	• 72% breastfed<br>• 79% vaccinated with PCV7<br>• 0% AB exposure prior 2w<br>• Mean number of siblings 1.3, SD 0.6<br>• 57% day-care attendance, 33% school attendance<br>• 13% tobacco smoke exposure<br><br>• -<br><br>• Cross-sectional study design<br>• Small cohort<br>• Swab and storage medium nr<br>• No information on pets
Mansbach *et al.*(19)<br><br>USA<br><br>2019	Multi-centre, prospective cohort study (2b)	557, 1,114<br>58	RSV bronchiolitis with hospitalisation (557, 1,114)<br>Median 3m, IQR 2-6m<br>During hospitalisation, 3w after hospitalisation<br><br>During hospitalisation:<br>Nasal wash with saline (*Medline Industries*), nr<br>On ice immediately, 4°C within 1h, -80°C within 24h<br>3w after hospitalisation:<br>Nasopharyngeal swab, FLOQSwab (*Copan*), nr<br>Ambient temperature time nr, -80°C<br><br>PowerSoil DNA Isolation Kit (*Mo Bio*)<br>nr<br>16S rRNA, V4, MiSeq (*Illumina*)<br>2x250, >1,000 reads/sample<br>SILVA	**Composition**<br>• 4 profiles: *Moraxella-, Streptococcus-, Haemophilus*-dominated, and mixed<br><br>**Delayed clearance of RSV (>3w)**<br>• Delayed clearance of RSV higher in infants with *Haemophilus*-dominated profile	• 11% maternal smoking during pregnancy<br>• 19% preterm born<br>• 65% vaginally born<br>• 51% breastfed<br>• 18% exposed to AB for current infection<br>• 13% tobacco smoke exposure<br>• 54% non-Hispanic white, Non-Hispanic black 17%, Hispanic 24%, other 5%<br><br>• Large sample number<br><br>• Storage medium nr<br>• No information on vaccination status, siblings, pets<br>• Overlap with participants from(16-18, 20-26)
McCauley *et al.*(52)<br><br>USA<br><br>2019	Multi-centre, prospective cohort study (participants from a randomised placebo-	413, 3,122<br>62	Asthma (413, 3,122)<br>Range 6-17y, bi-weekly during 3m<br><br>Nasal wash with saline, M4RT transport media (*Thermo Fisher Scientific*)<br>nr	**Composition**<br>• 6 profiles: *Moraxella-, Staphylococcus-, Corynebacterium-, Streptococcus-, Dolosigranulum-, Haemophilus*-dominated<br><br>**Exacerbation risk and ARTIs**	• Back non-Hispanic 58%, Hispanic 34%, mixed 6%, White non-Hispanic 2%<br>• Season of collection: 100% autumn/winter<br><br>• Longitudinal sample collection<br>• Large cohort

	controlled trial) (2b)		Modified cetyltrimethylammonium bromide polyethylene glycol protocol<br>515F, 806R<br>16S rRNA, V4, NextSeq 500 (*Illumina*)<br>2x250, >2,000 reads/sample<br>GreenGenes 13_5	• Stable composition over time despite viral infections or exacerbations<br>• *Moraxella*-dominated profiles associated with increased exacerbation risk<br>• *Staphylococcus*- or *Corynebacterium*-dominated profiles associated with reduces respiratory illness and exacerbation risk<br>• *Streptococcus*-dominated profiles associated with increased risk of rhinovirus infection	• Large sample number<br><br>• Includes children with corticosteroid treatment<br>• Includes children across a wide age range<br>• Storage conditions nr<br>• Sequencing depth nr<br>• No information on vaccination status, AB exposure, siblings, pets, tobacco smoke exposure
Stewart *et al.*(25)<br><br>USA<br><br>2019	Multi-centre, cross-sectional study (4)	140, 140<br>60	Bronchiolitis with hospitalisation (140) (RSV 63 rhinovirus 28, RSV plus rhinovirus 16)<br>Median 3m, IQR 1-6m<br><br>Nasal wash (*Medline Industries*), nr<br>On ice immediately, then -80°C<br><br>PowerSOIL DNA isolation kit (*Mo Bio*)<br>515F, 806R<br>16S rRNA, V4, MiSeq (*Illumina*)<br>2x250, 1,500 reads/sample<br>SILVA<br><br>Shotgun metagenomics (70)<br>nr<br>HiSeq (*Illumina*)<br>2x100, nr<br>Custom database	**Composition**<br>• Most abundant genera *Moraxella, Streptococcus,* and *Haemophilus*<br><br>**Positive pressure ventilation**<br>• Abundance of *Streptococcus* positively correlated with metabolites (glucuronate and 1-palmitoyl-2-palyitoleoyl-GPC 16:0/16:1) associated with a higher risk of needing positive pressure ventilation and negatively correlated with metabolites (plasmalogen sub-pathway) associated with a lower risk of needing positive pressure ventilation, abundance of *Moraxella* had opposite correlation	• 12% maternal smoking during pregnancy<br>• 23% preterm born<br>• 63% vaginally born<br>• 47% breastfed first 3m<br>• 11% passive tobacco smoke exposure<br>• Non-Hispanic white 39%, non-Hispanic black 21%, Hispanic 36%, other 3%<br><br>• Analysis at species level*<br><br>• Cross-sectional study design<br>• Storage medium nr<br>• No information vaccination status, antibiotic exposure, siblings, pets<br>• Overlap with participants from(16-24, 26)
Walker *et al.*(53)<br><br>New Zealand<br><br>2019	Multi-centre, prospective case-control study (3b)	178, 178<br>58	Healthy (105, 105, mean 50m, SD 6.6m, range 3-4y)<br>Chronic otitis media with effusion (73, 73, mean 48m, SD 6.7m, range 3-4y)<br><br>Nasopharyngeal swab, FLOQSwab (*Copan*), STGG<br>-80°C, time nr<br><br>Allprep Kit (*Qiagen*)<br>27F, 534R<br>16S rRNA, V1-V3, MiSeq (*Illumina*)<br>2x300, median 21,255, range 1,011-80,297 reads/sample in children with chronic otitis, median 9,562, range 1,100-82,013 in healthy children<br>GreenGenes	**Composition**<br>• 300 OTUs<br>• Most abundant genera *Corynebacterium, Moraxella, Streptococcus, unknown Bacilli (Staphylococcus), Dolosigranulum pigrum,* and *Neisseria*<br>• 4 profiles: *Corynebacterium-*, *Moraxella-, Streptococcus-*dominated, and a mixed profile<br><br>**Chronic otitis media with effusion**<br>• Lower diversity<br>• More frequently *Corynebacterium-*, *Moraxella-,* and *Streptococcus*-dominated profiles<br>• Higher abundance of *M. catarrhalis, M. caprae, S. pneumoniae, and H. influenzae*<br>• Lower abundance of *Streptococcus* OTUs of uncertain species, *S. infantis, C. acnes, Lactococcus, Neisseria, Lautropia, Capnocytophaga,* and two *Oxalobacteraceae* OTUs	• 66% vaginally born<br>• 88% fully vaccinated<br>• 10% AB prior 4w<br>• 60% siblings<br>• European and other 54%, Asian 13%, Māori 19%, Pacific Islander 15%<br>• Season of collection: 16% autumn, 35% winter, 60% spring, 15% summer<br><br>• Inclusion of healthy controls<br>• Analysis at species level*<br><br>• No information on pets, tobacco smoke exposure
Yau *et al.*(54)<br><br>Hong Kong<br><br>2019	Multi-centre prospective case-control study<br>(3b)	40, 40<br>nr	Healthy (17, 17), allergic rhinoconjunctivitis (23, 23)<br>Range 6-18y<br><br>Nasopharyngeal swab, FLOQSwab (*Copan*), nr<br>4°C immediately, -80°C time nr	**Composition**<br>• 3389 SVs<br>• Most abundant phyla Proteobacteria (55%), Firmicutes (24%), and Actinobacteria (20%)	• -<br><br>• Inclusion of healthy controls<br><br>• Small cohort<br>• Includes children across a wide age range

			PureLink Microbiome DNA Purification Kit (*ThermoFisher Scientific*)<br>314F, 806R<br>16S rRNA, V3-V4, MiSeq (*Illumina*)<br>2x300, mean 32,857 reads/sample (includes ocular samples)<br>SILVA 132	• Most abundant genera *Moraxella* (45%), *Corynebacterium* (18%), *Streptococcus* (5%), *Staphylococcus* (7%), and *Dolosigranulum* (11%)<br><br>**Allergic rhinoconjunctivitis**<br>• Higher diversity<br>• Association between higher diversity and disease severity	• Storage medium nr<br>• No information on sex, vaccination status, AB exposure, siblings, pets, tobacco smoke exposure
Ederveen *et al.*(55)<br><br>Netherlands<br><br>2018	Multi-centre, cross-sectional study (4)	75, 100<br>43	Healthy (21, 21), RSV infection (54, 11% co-infection with coronavirus, 20% with rhinovirus), During hospitalisation (54), 4-6w after (25)<br>Mean 82d, range 12d-6.5m<br><br>Nasal wash with sterile saline, nr<br>Kept cold, -80°C time nr<br><br>Phenol/bead beating method combined with the AGOWA Mag Mini DNA Extraction Kit (*AGOWA, LGC Genomics)*<br>341F/785R<br>16S rRNA, V3-V4, MiSeq (*Illumina)*<br>2x300, mean 25,000, SD 15,000 reads/sample<br>RDP 16S rRNA training set 9	**Composition**<br>• 156 genera, mean 152 OTUs/sample<br>• Most abundant genera *Haemophilus* (31%), *Streptococcus* (29%), *Moraxella* (11%), *Corynebacterium* (6.89%), *Prevotella* (4%)*, Achromobacter* (4%), *Staphylococcus* (3%), *Neisseria* (2%), and *Veillonella* (1%)<br><br>**RSV infection**<br>• Association between RSV viral load an overall composition of the microbiome<br>• Lower abundance of *Veillonella,* higher abundance of *Haemophilus,* and *Achromobacter*<br>• Positive correlation between abundance of *Haemophilus* and CXCL8 levels (indicative for higher disease severity)	• 45% preterm born<br>• 56% breastfed<br>• 8% currently exposed to AB<br><br>• Inclusion of healthy controls<br><br>• Cross-sectional study design<br>• Small cohort<br>• Storage medium nr<br>• Includes a large number of preterm born infants<br>• No information on delivery mode, vaccination status, siblings, day-care attendance, pets, tobacco smoke exposure
Kelly *et al.*(56)<br><br>Botswana<br><br>2018	Multi-centre, cross-sectional study (4)	170, 170<br>49	Healthy (88, 88), URTI (82, 82)<br>Mean 8.3m, range 1-23m<br><br>Nasopharyngeal swab, FLOQ (*Copan*), nr<br>On ice immediately, -80°C time nr<br><br>*In-house* protocol<br>16S rRNA, V3, MiSeq (*Illumina)*<br>341F, 518R<br>nr, mean 53,039 reads/sample<br>GreenGenes 2011	**Composition**<br>• 10 phyla 155 genera, 336 OTU<br>• Positive association between abundance of *S. pneumoniae* and *Moraxella*<br>• Negative association between abundance of *S. pneumoniae* and *Corynebacterium* and *Staphylococcus*<br><br>**Colonisation with *S. pneumoniae***<br>• No difference in diversity<br>• More frequent in older children, children who live without electricity or in households which use wood as cooking fuel<br><br>**ARTIs**<br>• More frequent in children colonised with *S. pneumoniae*	• 19% perinatally HIV-exposed<br>• 55% currently breastfed<br>• 69% >1 dose PCV13<br>• 5% AB prior 7d<br>• 64% during dry season, 36% during wet season<br><br>• Inclusion of healthy controls<br>• Analysis at species level*<br><br>• Cross-sectional study design<br>• Storage medium nr<br>• Sequencing length nr<br>• No information on delivery mode, gestational age, siblings, day-care attendance, pets, tobacco smoke exposure<br>• Overlap with participants from(57)
Lappan *et al.*(58)<br><br>Australia<br><br>2018	Multi-centre, cross-sectional study (4)	196, 196<br>57	Prone to AOM receiving tympanostomy tubes (93, 93, median 1.9y, IQR 1.3-2.8), controls (92 healthy, 11 with chronic illness, 103, median 1.6y, IQR 1.5-3.2y)<br><br>Nasopharyngeal swab, FLOQSwab (*Copan*), STGG<br>On ice immediately, -80°C time nr<br><br>Wizard SV Genomic DNA System (*Promega*)<br>27F, 515F	**Composition**<br>• 123 OTUs<br>• Positive correlation between abundance of *Corynebacterium* and *Dolosigranulum* in healthy children<br><br>**Prone to AOM**<br>• Higher diversity<br>• Lower abundance of *Corynebacterium* and *Dolosigranulum*<br>• Higher abundance of *Neisseria, Gemella, Porphyromonas, Alloprevotella, Fusobacterium* (almost absent in controls)	• 92% breastfed (11% currently)<br>• 37% antibiotic exposure prior 4w<br>• 59% sibling <5y<br>• 79% day-care attendance<br>• 12% tobacco smoke exposure<br>• Season of collection: 18% autumn, 50% winter, 26%, spring, 6% summer<br><br>• Inclusion of controls

			16S rRNA, V3-V4, MiSeq (*Illumina*)<br>2x465, >1,499 reads, mean 161,734 reads/sample (includes samples from middle ear and ear canal)<br>SILVA v123	• Positive correlation between abundance of *Haemophilus* and *Moraxella*, Gemella and *Porphyromonas, Gemella* and *Neisseria*	• Cross-sectional study design<br>• No information on vaccination status, pets
Luna *et al.*(21)<br><br>USA<br><br>2018	Multi-centre, prospective cohort study (2b)	815, 1,630<br>60	Bronchiolitis with hospitalisation (815, 1,630)<br>Median 3m, IQR 2-6m<br>During hospitalisation, 3w after hospitalisation, during summer, 1y after hospitalisation<br><br>Nasopharyngeal swab, FLOQSwab (*Copan*), nr<br>Nasal wash normal saline, sterile tubes (*Medline Industries*)<br>4°C within 1h, -80°C within 24h<br><br>PowerSoil DNA Isolation Kit (*Mo Bio*)<br>nr<br>16S rRNA, V4, MiSeq (*Illumina*)<br>2x250, >1,000 reads/sample<br>SILVA	**Composition**<br>• Nasopharyngeal swab: *S. aureus* (41%)*, Corynebacterium* (10%)*, Moraxella* (9%)*, Haemophilus* (7%)*, Dolosigranulum* (5%)*, Streptococcus* (5%)*, Enterobacter* (5%), lower richness and diversity compared with nasopharyngeal aspirate<br>• 6 profiles: *Haemophilus*-dominant (7.2%), *Moraxella*-dominant (13.0%), *Staphylococcus*-dominant (44.5%), *Corynebacterium*-dominant (13.4%), *Enterobacter*-dominant (7.5%), and mixed (14.4%) profiles<br>• Nasopharyngeal aspirate: *Moraxella* (31%)*, Streptococcus* (31%), and *Haemophilus* (20%)<br><br>**Age**<br>• Infants less than 6 months more frequently had a *Staphylococcus*-dominant profile<br><br>**Antibiotics**<br>• Infants with AB exposure before hospitalisation more frequently had a *Haemophilus*-dominant profile<br><br>**Bronchiolitis**<br>• *Haemophilus-dominant* profile associated with increased severity of bronchiolitis and length of hospital stay<br>• *Moraxella-dominant* profile associated with less frequent admission to ICU	• 14% maternal smoking during pregnancy<br>• 17% preterm born<br>• 66% vaginally born<br>• 44% breastfed<br>• 31% prior AB, time nr<br>• 23% day-care attendance<br>• 16% tobacco smoke exposure<br><br>• Longitudinal sample collection<br>• Large cohort<br>• Large sample number<br><br>• Storage medium nr<br>• No information on vaccination status, siblings, pets<br>• Overlap of participants with(16-20, 22-26)
Pérez-Losada *et al.*(59)<br><br>USA<br><br>2018	Multi-centre, prospective cohort study (2b)	163, 205<br>53	Asthma (163)<br>Mean 11.0y, range 6-18y<br>Baseline (163), 6m after (42)<br><br>Nasal wash with sterile saline buffer, nr<br>On ice immediately, then -80°C time nr<br><br>QIAamp DNA Kit (Qiagen)<br>nr<br>16S rRNA, V4, MiSeq (*Illumina*)<br>2x250, mean 25,932, range 530-160,718 reads/sample<br>SILVA 128	**Composition**<br>• 8034 OTUs<br>• Most abundant genera *Moraxella* (28%), *Staphylococcus* (18%), *Corynebacterium* (10%), *Dolosigranulum* (8%), *Prevotella* (6%), *Streptococcus* (6%), *Fusobacterium* (3%), and *Haemophilus* (3%)<br>• Most abundant OTUs *Moraxella, Staphylococcus, Streptococcus,* and *Haemophilus*<br><br>**Asthma**<br>• Association between asthmatic phenotype and microbiome composition<br>• Children who were older when diagnosed, had persisting symptoms despite treatment and a higher BMI had a higher abundance of *Corynebacterium* and *Prevotella* and a lower abundance of *Moraxella* and *Dolosigranulum*<br>• Children who were youngest at diagnosis, had a high rate of positive allergen tests, high blood eosinophil and IgE levels and a high rate of using inhaled corticosteroids had a lower diversity and a higher abundance of *Moraxella* and a lower abundance of and *Corynebacterium, Staphylococcus,* and *Prevotella*	• 81% African Americans, 2% White, 17% other<br><br>• -<br><br>• Includes children with corticosteroid treatment<br>• Includes children across a wide age range<br>• Includes samples with low number of reads<br>• Storage medium nr<br>• No information on vaccination status, AB exposure, siblings, pets, tobacco smoke exposure<br>• Overlap with participants from(60, 61)

				• Children with lower BMI, low rate of positive skin prick test and better response to treatment with bronchodilators had an intermediate abundance of the five genera	
Rosas-Salazar *et al.*(4)<br><br>USA<br><br>2018	Multi-centre, prospective cohort study (2b)	118, 125<br>58	RSV infection (118, 125)<br>21.8w, IRQ 12.1-27.1w<br><br>Nasal wash with sterile saline, nr<br>-80°C time nr<br><br>Phenol:chloroform:isoamyl alcohol extractions<br>515F, 806R<br>16S rRNA, V4, MiSeq (*Illumina*)<br>2x300, median 18,130, range 13,240-25,970 reads/sample<br>SILVA 138	**Composition**<br>• 357 genera (median 19, IQR 13-31)<br>• Most abundant genera *Moraxella* (38%), *Streptococcus* (20%), *Haemophilus* (14%), *Corynebacterium* (19%), and *Dolosigranulum* (5%)<br><br>**Recurrent wheezing**<br>• Lower abundance of *Lactobacillus* and *Staphylococcus* during RSV infection associated with recurrent wheezing at 24m	• 0% preterm born<br>• 58% vaginally born<br>• 73% breastfed<br>• 62% AB exposure (*in utero* or before enrolment)<br>• 26% tobacco smoke exposure through mother<br>• Black non-Hispanic 17%, White non-Hispanic 63%, Hispanic 9%, other 11%<br><br>• -<br><br>• Storage medium nr<br>• No information on vaccination status, day-care attendance, pets<br>• Overlap with participants from(1-3)
Teo *et al.***(62)**<br><br>Australia<br><br>2018	Multi-centre, prospective birth cohort study (2b)	244, 3,014<br>57	Healthy at risk for allergic sensitisation (244)<br>Birth to 5y every 2m, afterwards every 6m (1,331), during ARTI (URTI 996, LRTI 1,055)<br><br>Nasopharyngeal aspirate, nr<br>-80°C time nr<br><br>Wizard SV Genomic DNA System (*Promega*)<br>515F, 806R<br>16S rRNA, V4, MiSeq (I*llumina*)<br>2x151, median 147,000, IQR 45,000-230,000 reads/sample<br>GreenGenes 13_5	**Composition**<br>• Most abundant genera *Moraxella* (40.1%), *Streptococcus* (13.3%), *Corynebacterium* (12.1%), *Dolosigranulum* (11.1%), *Haemophilus* (8.6%), *and Staphylococcus* (4.2%)<br>• 15 profiles: *Moraxella-, Streptococcus-, Haemophilus-, Corynebacterium-, Dolosigranulum/Corynebacterium-, Staphylococcus-, Streptococcus-, Moraxellaceae*-dominated or mixed<br><br>**Age**<br>• Diversity increased with age, especially >2y<br><br>**ARTI**<br>• Higher frequency of *Haemophilus-, Streptococcus-,* and *Moraxella*-dominated profiles during ARTIs, a higher abundance of one of these bacteria during ARTI was associated with a decrease in diversity and increase in ARTI severity<br>• Lower abundance of *Staphylococcus* (<4y), *Corynebacterium (*<3y), and *Dolosigranulum*<br>• Abundance of *Streptococcus* closely matching to *S. gordonni, S. thermophilus/salivarius/vestibularis* negatively associated with risk for ARTI<br>• Increase in abundance of *Moraxella* 1-2m prior to ARTI	• 0% vaccinated with PCV<br><br>• Large sample number<br>• Analysis at species level*<br><br>• Storage medium nr<br>• No information on delivery mode, gestational age, feeding method, AB exposure, siblings, day-care attendance, pets, tobacco smoke exposure<br>• Overlap with participants from(63)
Wen *et al.***(47)**<br><br>China<br><br>2018	Single-centre, prospective cohort study (2b)	180, 180<br>56	Healthy (59, 59), influenza A infection (121, 121)<br>nr<br><br>Nasopharyngeal swab, 25-800-A-50 (*Puritan*)<br>-80°C within 10min<br><br>Power Soil DNA Isolation Kit (Mo Bio)<br>16S rRNA, V3-V4, MiSeq (*Illumina*)	**Influenza A infection**<br>• Higher diversity<br>• Lower abundance of *Moraxella, Staphylococcus, Corynebacterium,* and *Dolosigranulum,*<br>• Higher abundance of *Phyllobacterium*, *Acinetobacter,* unclassified *Acidobacteria, Ralstonia, Pseudomonas, Lachnoclostridium* and *Halomonas*	• 67% vaginally born<br>• 48% exclusively breastfed<br><br>• Inclusion of healthy controls<br><br>• Sequencing length nr<br>• No information on age, AB exposure, siblings, day-care attendance, pets, tobacco smoke exposure<br>• Overlap of participants with(45, 46, 48)

			nr, mean 26,063 reads/sample for healthy children, 25,592 for children with influenza A infection<br>Live Tree Project		
Bosch *et al.*(29)<br><br>Netherlands<br><br>2017	Single-centre, prospective birth cohort study (2b)	112, 1,121<br>48	Healthy (112, 1,121)<br>0, 1, 7, 14 d, 1, 2, 3, 4, 6, 9, 12m, within 48h of ARTI<br><br>Nasopharyngeal swab, ESwab (*Copan*)<br>Frozen immediately, then -80°C<br><br>Modified Mag Forensics Extraction Kit protocol (*LGC Genomics*)<br>nr<br>16S rRNA, V4, MiSeq (*Illumina*)<br>2x240-260, median 20,670 reads/sample, range 3,911-97,870<br>SILVA v119	**Composition**<br>• 14 phyla, 576 OTUs<br>• Factors that influenced composition most were age, presence of siblings <5y of age, season of birth, breastfeeding for at least 3m, delivery mode, AB exposure prior 30d<br><br>**Delivery mode**<br>• Infants born vaginally had a prolonged predominance of *Corynebacterium* and *Dolosigranulum,* and a late enrichment of *Moraxella*<br>• Infants born by CS had a higher abundance of *Gemella* and *Streptococcus* from birth<br>• Infants born by CS had a prolonged predominance of *Neisseria, Prevotella, Granulicatella,* and *Actinomyces*<br><br>**Feeding method**<br>• Breastfed infants had an early abundance of Dolosigranulum and prolonged predominance of *Corynebacterium* and *Dolosigranulum,* as well as a late enrichment of *Moraxella*<br>• Higher abundance of *Gemella* and *Streptococcus* in formula-fed infants from birth<br>• Higher abundance of *Streptococcus, Prevotella, Porphyromonas,* and *Veillonella* in formula-fed infants >1m<br>• Prolonged predominance of *Neisseria, Prevotella, Granulicatella, Actinomyces* in formula-fed infants >1m<br><br>**Age**<br>• 1d: most abundant phyla Firmicutes with *Staphylococcus, Dolosigranulum,* and *Streptococcus*<br>• 1d-1m: increase in absolute bacterial abundance<br>• 12m: most abundant phyla Protobacteria with *Moraxella* and *Haemophilus*<br><br>**ARTI**<br>• Less stable composition<br>• Higher abundance of *Moraxella* and *Haemophilus* early in life<br>• Higher abundance of *Neisseria*, *Prevotella,* and *Alloprevotella* from 2m of life<br>• Absence or lower abundance of *Corynebacterium*, *Dolosigranulum,* and *Streptococcus*<br><br>**ARTI**(30) (843)<br>• Increase in abundance of *N. lactamica, Streptococcus, P. nanceiensis, Fusobacterium,* and *J. lividum* before and during ARTIs<br>• Decrease in presence and abundance of *Corynebacterium, Dolosigranulum,* and *Moraxella* before and during ARTIs	• 63% vaginally born<br>• 31% AB exposure<br>• 59% siblings <5y of age<br>• 3% tobacco smoke exposure<br>• 70% day-care attendance<br><br>• Longitudinal sample collection<br>• Large number of samples<br>• Analysis at species level*<br><br>• Overlap of participants with(27, 28, 30)

				**Antibiotic exposure**<br>• Lower abundance of *Corynebacterium* and *Dolosigranulum*<br><br>**Siblings**<br>• Accelerated microbiome maturation<br>• Higher abundance of *Pasteurellaceae*<br><br>**Day-care attendance**<br>• Accelerated microbiome maturation<br>• Higher abundance of *Moraxella* and lower abundance of *Staphylococcus*	
Chonmaitree *et al.*(64)<br><br>USA<br><br>2017	Single-centre, prospective birth cohort study (2b)	139, 948<br>60	Healthy (74, 680)<br>URTI (65, 223 during URTI, 45 URTI plus AOM), 1, 2, 3, 4, 5, 6, 9m<br><br>Nasopharyngeal swab, ESwab (*Copan*)<br>-80°C within 1h<br><br>PowerMicrobiome DNA/RNA isolation kit (*Mo Bio*)<br>nr<br>16S rRNA, V4, MiSeq (*Illumina*)<br>250, >1,000/sample<br>SILVA v119	**Composition**<br>• 13,982 OTUs, 21 genera with ≥0.5% abundance<br>• Most abundant phyla Proteobacteria (39%), Actinobacteria (26%), Firmicutes (26%) and Bacteroidetes (6%)<br>• Most abundant genera *Corynebacterium, Moraxella, Dolosigranulum, Staphylococcus, Acinetobacter, Haemophilus, Pseudomonas, Streptococcus, Bifidobacterium, Enterobacter, Micrococcus, Arhodomonas,* and *Bacteroides*<br>• Lower diversity when colonised with *S. pneumoniae* or *H. influenzae*<br><br>**Age**<br>• Positive correlation between diversity at 1m and at later age<br><br>**URTI**<br>• No influence on diversity<br>• Higher abundance of *Moraxella, Haemophilus, Streptococcus,* lower abundance of *Yersinia, Pseudomonas, Myroides,* and *Sphingomonas*<br>• Higher diversity at 1m associated with frequent URTIs within first 6m but not with number of AOM<br>• Higher abundance of *Moraxella* and *Streptococcus* during symptomatic infection with positive viral PCR<br><br>**AOM**<br>• No influence on diversity<br>• Higher abundance of *Moraxella*, *Haemophilus*, and *Streptococcus*<br>• Higher abundance of *Haemophilus, Enterobacter, Bifidobacterium*, *Yersinia*, and lower abundance of *Corynebacterium*, *Myroides*, and *Pseudomonas* during first 12m in infants who developed AOM<br>• Frequent AOM in first 6m associated with decreasing abundance of *Micrococcus*<br>• Reduced risk of AOM after URTI when higher abundance of *Staphylococcus* and *Sphingobium*<br><br>**Antibiotic exposure prior 6m (mostly amoxicillin for 7-10d)**<br>• Higher diversity, highest 7d after exposure	• 5% exclusively breastfeed for 3m<br>• 9% exclusively breastfeed for 6m<br>• 71% AB exposure prior 2m<br>• 40% ≥ 1 sibling<br>• 24% day-care attendance<br><br>• Longitudinal sample collection<br>• Inclusion of healthy controls<br>• Large sample number<br><br>• High number of infants exposed to AB<br>• No information on delivery mode, gestational age, vaccination status, pets, tobacco smoke exposure

				• Lower abundance of *Corynebacterium* and *Dolosigranulum* within 7d, *Enterobacter* within 7 to 14d, *Staphylococcus* 14 to 30d, and higher abundance of *Bifidobacterium* and *Firmicutes incertae sedis* at 7d<br>• Lower abundance of *Staphylococcus* within 14 to 30d<br><br>**No association between age, sex, ethnicity, mode of delivery, and breastfeeding and diversity**	
Kelly *et al.*(57)<br><br>Botswana<br><br>2017	Multi-centre, cross-sectional study (4)	319, 319<br>50	Healthy (60, 60, 8m), pneumonia (204, 14 HIV infected, 204, mean 8.2m), URTI (55, mean 11.4m)<br><br>Nasopharyngeal swab, FLOQ (*Copan*), nr<br>On ice immediately, -80°C time nr<br><br>*In-house* protocol<br>16S rRNA, V3, MiSeq (*Illumina)*<br>341F, 518R<br>nr, mean 47,075 reads/sample<br>GreenGenes 2011	**Composition**<br>• 12 phyla, 145 genera, 285 OTUs<br>• 5 profiles: *Corynebacterium/Dolosigranulum*- (23%), *Haemophilus*- (11%), *Moraxella*- (24%), *Staphylococcus*- (13%), and *Streptococcus*-dominant (28%)<br><br>**Age**<br>• Children >12m higher abundance of *Moraxella* and lower abundance of *Staphylococcus*<br><br>**URTI**<br>• *Moraxella*- and *Streptococcus*-dominant profiles more frequent<br>• Higher abundance of *Haemophilus, Streptococcus,* and *Moraxella*<br>• Lower abundance of *Staphylococcus*<br><br>**Pneumonia**<br>• *Haemophilus-, Staphylococcus-,* and *Streptococcus*-dominant profiles more frequent<br>• Higher abundance of *Haemophilus, Streptococcus, Escherichia,* and *Klebsiella*<br>• Lower abundance of *Corynebacterium* and *Dolosigranulum*<br><br>**HIV infection**<br>• Associated with a lower abundance of *Dolosigranulum* in infants with pneumonia<br><br>**Perinatal HIV exposure**<br>• Higher abundance of *Klebsiella*	• 22% perinatally HIV-exposed<br>• 44% currently breastfed<br>• 46% > 1 dose PCV13<br>• 15% AB exposure prior 7d<br>• Samples: dry season 65%, wet season 35%<br><br>• Inclusion of healthy controls<br><br>• Cross-sectional study design<br>• Storage medium nr<br>• Sequencing length nr<br>• No information on delivery mode, gestational age, siblings, day-care attendance, pets, tobacco smoke exposure<br>• Overlap with participants from(56)
Lu *et al.*(46, 48)<br><br>China<br><br>2017	Single-centre, prospective cohort study (2b)	119, 119<br>56	Healthy (59, 59), pneumonia unknown pathogen (31, 31), *M. pneumoniae* pneumonia (29, 29)<br>nr<br><br>Nasopharyngeal swab, 25-800-A-50 (*Puritan*)<br>-80°C within 10min<br><br>Power Soil DNA Isolation Kit (Mo Bio)<br>16S rRNA, V3-V4, MiSeq (*Illumina*)<br>nr, mean 37,987 reads/sample for healthy children, 42,972 for children with pneumonia<br>Live Tree Project	**Pneumonia**<br>• Lower diversity<br>• Higher abundance of Firmicutes, lower abundance of Bacteroidetes<br>• Lower abundance of *Prevotella*, *Moraxella and Dolosigranulum,* higher abundance of *Mycoplasma, Streptococcus, Staphylococcus, Lactobacillus, Ralstonia, Acinetobacter* and *Actinomyces*	• 65% vaginally born<br>• 49% exclusively breastfed<br><br>• Inclusion of healthy controls<br><br>• Sequencing length<br>• No information on gestational age, age, vaccination status, AB exposure, siblings, day-care attendance, pets, tobacco smoke exposure<br>• Overlap with participants from(45, 47)

Perez *et al.*(65)<br><br>USA<br><br>2017	Single-centre, cross-sectional study (4)	13, 19<br>54	Hospitalisation with rhinovirus infection (13, 13): term born (6, median 1.8y, IQR 0.8-2.5y), preterm born (7, median 1.4y, IQR 0.8-1.9y), 2-3m after hospitalisation (6, 6)<br><br>Nasal wash with sterile saline, nr<br>nr<br><br>*In-house* protocol<br>B27F, U1492R<br>16S rRNA, V1-V2, PacBio RS II (*Pacific Biosystems*)<br>1466, mean 5,157, range 1,037-8,914 reads/sample<br>SILVA v123	**Composition**<br>• 8 phyla, 75 genera, 592 OTUs (mean 53/sample)<br>• Most abundant genera *Streptococcus* (34%), *Moraxella* (19%), *Staphylococcus* (10%), *Burkholderia* (9%), *Neisseria* (6%), *Haemophilus* (6%), and *Janthinobacterium* (5%)	• 54% preterm born<br>• 46% Black<br>• 0% AB prior 4w to enrolment<br><br>• -<br><br>• Cross-sectional study design<br>• Small cohort<br>• Storage medium and conditions nr<br>• No information on delivery mode, feeding methods, vaccination status, siblings, day-care attendance, pets, tobacco smoke exposure
Pérez-Losada *et al.*(60)<br><br>USA<br><br>2017	Multi-centre, prospective cohort study (2b)	40, 80<br>60	Asthma (40)<br>Mean 11.0y, SD 3.4y, range 6-18y<br>Baseline (40), 6m after (40)<br><br>Nasal wash with sterile saline buffer, nr<br>On ice immediately, then -80°C time nr<br><br>QIAamp DNA Kit (Qiagen)<br>nr<br>16S rRNA, V4, MiSeq (*Illumina*)<br>2x250, mean 26,207, range 2,288-60,806 reads/sample<br>SILVA 123	**Composition**<br>• 396 genera, mean 167 OTUs/sample, range 34-394<br>• Most abundant genera *Moraxella* (35%), *Staphylococcus* (14%), *Dolosigranulum* (9%), *Corynebacterium* (9%), *Prevotella* (6%), *Streptococcus* (5%), *Haemophilus* (4%), and *Fusobacterium* (3%)<br><br>**Age**<br>• Influenced abundance of *Haemophilus, Staphylococcus,* and *Corynebacterium*<br><br>**Season**<br>• No association between season and diversity<br>• Higher abundance of *Haemophilus* in summer	• 95% Non-Hispanic or Latino, 5% Hispanic or Latino<br><br>• -<br><br>• Includes children with corticosteroid treatment<br>• Includes children across a wide age range<br>• Storage medium nr<br>• No information on vaccination status, AB exposure, siblings, pets, tobacco smoke exposure<br>• Overlap with participants from(59, 61)
Salter *et al.*(66)<br><br>Thailand<br><br>2017	Single-centre, prospective cohort study (2b)	21, 517<br>24	Healthy (14, 477, monthly from birth to 24m; 7, 40 during LRTI)<br><br>Nasopharyngeal swab, dacron tipped swab (*Medical Wire and Equipment Co*), STGG<br>-80°C within 8h<br><br>FastDNA spin kit for soil (*MP Biomedicals*)<br>338F, 926R<br>16S rRNA, V3-V4, GS FLX Titanium (*454 Life Sciences/Roche)*<br>400 bp, 200 reads/sample<br>RDP and SILVA v123	**Composition**<br>• 297 OTUs<br>• Most abundant genera *Moraxella, Streptococcus, Haemophilus, Corynebacterium,* unclassified *Flavobacteriaceae* I, and *Helococcus*<br>• Positive association between abundance of *Streptococcus* I and *Corynebacterium* II (including *C. accolens)*, *Streptococcus* I and *Staphylococcus*, *Staphylococcus* and *Corynebacterium* II (including *C. accolens*)<br>• Negative association between abundance of *Streptococcus* I and *Dolosigranulum* and *Staphylococcus* and *Corynebacterium* I (including *C. pseudodiphtheriticum*)<br>• Composition similar between children<br><br>**Age**<br>• First 3m: *Staphylococcus, Streptococcus, Corynebacterium, Moraxella;* higher abundance of *Staphylococcus* and *Corynebacterium* compared with later<br>• First 12m: increase in abundance of unclassified *Flavobacteriaceae*<br>• First 21m: increase in abundance of *Moraxella*	• 5% preterm born<br>• 90% born vaginally<br>• 100% breastfed until 12m, introduction of solid foods at 4 to 6m<br>• 0% vaccinated against *H. influenzae* or *S. pneumoniae*<br>• 86% ≥ 1 sibling<br>• 8% AB exposure prior 7d<br>• 33% tobacco smoke exposure<br><br>• Longitudinal sample collection<br>• Large sample number<br>• Inclusion of healthy controls<br>• Analysis at species level*<br><br>• Small cohort<br>• Low sequencing depth<br>• No information on gestational age, siblings, pets

				• 2 to 12m: decrease in abundance of *Streptococcus*<br><br>**LRTI**<br>• Perturbation different from infant to infant and illness episode (both increase and decrease of diversity)<br>• Acquisition of new S. *pneumoniae* serotype not associated with disease<br><br>**During AB exposure (mostly oral amoxicillin for 7d)**<br>• Lower abundance of *Moraxella* and higher abundance of *Brachybacterium*, *Dolosigranulum,* and *Streptococcus*	
Stewart *et al.*(26)<br><br>USA<br><br>2017	Multi-centre, cross-sectional study (4)	144, 144<br>15	Bronchiolitis with hospitalisation (144, 144, RSV 108, rhinovirus 40)<br>Median 3m, IQR 1-6m<br><br>Nasal wash (*Medline Industries*), nr<br>On ice immediately, then -80°C<br><br>PowerSOIL DNA isolation kit (*Mo Bio*)<br>515F, 806R<br>16S rRNA, V4, MiSeq (*Illumina*)<br>2x250, 1,500 reads/sample<br>SILVA<br><br>Shotgun metagenomics (70)<br>nr<br>HiSeq (*Illumina*)<br>2x100, nr<br>Custom database	**Positive pressure ventilation**<br>• Infants needing positive pressure ventilation had a higher abundance of *Streptococcus, Rothia, Klebsiella, Haemophilus* and a lower abundance of *M. catarrhalis*<br>• Sphingolipid metabolites were enriched in infants needing positive pressure ventilation and correlated to the abundance of *S. pneumoniae*	• 8% maternal smoking during pregnancy<br>• 22% preterm born<br>• 63% vaginally born<br>• 33% prior AB exposure<br>• Median number of siblings 1, IQR 0-5<br>• Non-Hispanic white 49%, non-Hispanic black 17%, Hispanic 28%, other 6%<br><br>• Analysis at species level*<br><br>• Cross-sectional study design<br>• Storage medium nr<br>• Includes children with corticosteroid treatment<br>• No information on feeding method, vaccination status, pets, tobacco smoke exposure<br>• Overlap with participants from(16-25)
Bosch *et al.*(28)<br><br>Netherlands<br><br>2016	Single-centre, prospective birth cohort study (2B)	102, 761<br>50	Healthy (102, 761)<br>0, 1, 7, 14 d, 1, 2, 3, 4, 6m<br><br>Nasopharyngeal swab, ESwab (*Copan*)<br>Frozen immediately, then -80°C<br><br>Modified Mag Forensics Extraction Kit protocol (*LGC Genomics*)<br>16S rRNA, V4, MiSeq (*Illumina*)<br>2x240-260, median 23,838 reads/sample, range 9,973-91,376<br>SILVA v119	**Composition**<br>• 1,354 oligotypes, (mean 103/sample at 1m, 129/sample at 3m)<br>• 9 profiles: *M. lincolnii*-, *M. catarrhalis/nonliquefaciens*-, *H. influenzae*-. *C. pseudodiphteriticum*-, *S. viridans*-, *Staphylococcus-Corynebacterium*-, *Corynebacterium-Dolosigranulum*-, *Moraxella-Corynebacterium-Dolosigranulum*-dominated, and a profile with high abundance of *S. aureus* at early time points and *M. catarrhalis/nonliquefaciens* later<br>• Most abundant genera *Corynebacterium* (25%), *Moraxella* (21%), *Staphylococcus* (19%), *Streptococcus* (11%), *Dolosigranulum* (11%), and *Haemophilus* (4%)<br>• Most abundant oligotypes *S. aureus* (19%), *M. catarrhalis /nonliquefaciens* (18%), *C. pseudodiphteriticum/propinquum* (17%), *D. pigrum* (11%), *S. viridans* (6%), *Corynebacterium* spp. (4%), *M. lincolnii* (3%), *S. pneumoniae* (2%), *H. influenzae* (2%)<br>• *H. influenzae*-dominated profile associated with less stable composition over time<br><br>**Age**<br>• Early in life mainly *S. aureus*-dominated profile, gradually replaced by *Moraxella*-, *Corynebacterium*-, *Dolosigranulum*-, and *Haemophilus*-dominated profile	• 0% preterm born<br>• 61% vaginally born<br>• 49% breastfed >3m, 19% exclusively breastfed first 6m<br>• 4% AB exposure first w, 12% in first 6m<br>• 55% siblings <5y of age<br>• 47% day-care attendance<br>• 1% tobacco smoke exposure<br>• Season of birth: spring 28%, summer 29%, autumn 20%, winter 23%<br>•<br><br>• Longitudinal sample collection<br>• Large sample number<br>• Analysis at species level*<br><br>• No information on vaccination status, pets<br>• Overlap of participants with(27, 29, 30)

				• Microbiome changed most during first 2m of life<br><br>**Delivery mode**<br>• No difference in diversity<br>• Infants born by CS stayed longer in a *S. aureus*-dominated profile- and changed later to a *Moraxella*- and *Corynebacterium/Dolosigranulum*-dominated profile<br>• Infants born by CS had a higher abundance of *S. aureus, S. viridans, Gemella,* and *S. Salivarius* and a lower abundance of *C. pseudodiptheriticium/propinquum* and *D. pigrum*<br><br>**ARTI**<br>• Abundance of *Haemophilus* associated with more frequent ARTIs	
Hasegawa *et al.*(17, 18, 22-24)<br><br>USA<br><br>2016	Multi-centre, prospective cohort study (2b)	1005, 1005 60	Bronchiolitis with hospitalisation (844)<br>Bronchiolitis with ICU admission (161)<br>Median 3.2m, IQR 1.6-5.9m<br><br>Nasal wash (*Medline Industries)*, nr<br>On ice immediately, then -80°C<br><br>PowerSOIL DNA isolation kit (*Mo Bio*)<br>nr<br>16S rRNA, V4, MiSeq (*Illumina*)<br>2x250, >2,128 reads/sample<br>SILVA	**Composition**<br>• 24 phyla, 379 genera<br>• Most abundant genera *Streptococcus* (31%), *Moraxella* (30%), *Haemophilus* (20%), *Prevotella* and *Staphylococcus*<br>• 4 profiles: *Haemophilus*-, *Moraxella-, Streptococcus*-dominated and a mixed profile with a high richness<br>• Negative association between abundance of *Haemophilus, Moraxella,* and *Streptococcus*<br>• Positive association between abundance of *Streptococcus* and *Veillonella*<br>• Positive association between abundance *Veillonella* and *Prevotella* and *Alloprevotella*<br><br>**Age**<br>• *Haemophilus*-dominated profile more frequent in older infants<br><br>**Antibiotic exposure**<br>• More frequently *Haemophilus*-dominated profile<br><br>**Ethnicity**<br>• Non-Hispanic white with more frequently *Haemophilus*-dominated profile<br><br>**Weight**<br>• Infants with *Haemophilus*-dominated profile had higher body weight<br><br>**Co-infection**<br>• Infants with *Haemophilus*-dominated profile more often infected with rhinovirus only (compared with co-infection with other or several viruses)<br><br>**Vitamin D serum level**(23)<br>• Infants with low vitamin D levels had a lower richness and diversity, higher abundance of *Staphylococcus*<br>• In infants with low vitamin D levels a *Haemophilus*-dominant profile was associated with a higher risk of ICU admission	• 14% maternal smoking during pregnancy<br>• 18% preterm born<br>• 64% vaginally born<br>• 42% breastfed<br>• 31% AB exposure before admission<br>• 15% corticosteroid exposure before admission<br>• 80% siblings<br>• 23% day-care attendance<br>• 15% tobacco smoke exposure<br>• 81% infected with RSV, 21% infected with Rhinovirus<br>• 22% admitted to ICU<br>• 43% non-Hispanic White, 23% non-Hispanic Black, 30% Hispanic, 4% other<br><br>• Large cohort<br>• Large sample number<br><br>• Storage medium nr<br>• No information on vaccination status, pets<br>• Overlap of participants with(16, 18-22, 25, 26)

				**Bronchiolitis** (sub-study with 60 infants)(18)<br>• Infants infected with alpha coronaviruses more often had a *Haemophilus*-dominated profile compared with infants infected with beta coronaviruses<br><br>**Bronchiolitis** (sub-study with 774)(22)<br>• Association between *Streptococcus*-dominated profile and RSV infection<br>• Association between *Haemophilus*-dominated profile and rhinovirus A infection<br>• Association between *Streptococcus*-dominated profile and rhinovirus C infection<br>• Infants infected with rhinovirus had lower abundance *Streptococcus*<br>• Infants infected with rhinovirus A had a higher abundance of *Haemophilus*<br><br>**ICU admission for severe bronchiolitis**<br>• Highest in infants with *Haemophilus*-dominated profile (this group also had a longer hospital stay)<br>• Lowest in infants with *Moraxella*-dominated profile<br><br>**Nasopharyngeal CCL5 levels(24)**<br>• In infants with low local CCL5 levels a *Haemophilus*-dominant profile was associated with a higher risk of ICU admission and longer hospital stay compared to a *Moraxella*-dominant profile	
Pérez-Losada *et al.*(61)<br><br>USA<br><br>2016	Multi-centre, prospective cohort study (2b)	30, 60<br>nr	Asthma (30, 60)<br>Range 6-17y<br><br>Nasal wash with sterile saline buffer, nr<br>On ice immediately, then -80°C time nr<br>Nasal brush, CytoSoft Cytology Brush No. CYB-1 (*Medical Packaging Corporation*), nr<br><br>QIAamp DNA Kit (*Qiagen)*<br>nr<br>16S rRNA, V4, MiSeq (*Illumina*)<br>2x250, mean 24,575, range 1,100-62,148 reads/sample<br>SILVA 123	**Composition**<br>• Nasal wash: mean 157 OTUs/sample, range 33-272<br>• Nasal brush: mean 186 OTUs/sample, range 120-408<br>• Higher diversity in nasal brush samples than nasal washes<br>• Higher abundance of *Moraxella, Staphylococcus, Prevotella, Haemophilus, Treponema, Streptococcus, Fusobacterium* and lower abundance of *Pseudomonas and Bacteroides* in nasal wash compared with nasal brush	• -<br><br>• -<br><br>• Includes children with corticosteroid treatment<br>• Includes children across a wide age range<br>• Storage medium nr<br>• No information on sex, vaccination status, AB exposure, siblings, pets, tobacco smoke exposure<br>• Overlap with participants from(59, 60)
Rosas-Salazar *et al.*(2)<br><br>USA<br><br>2016	Multi-centre, prospective cohort study (2b)	135, 135<br>58	ARTI (135, 135, human rhinovirus (52, median 18w, IQR 11-23w), RSV (83, median 19w, IQR 10-26w))<br><br>Nasal wash with sterile saline, nr<br>-80°C time nr<br><br>Phenol:chloroform:isoamyl alcohol extractions<br>515F, 806R<br>16S rRNA, V4, MiSeq (*Illumina*)	**Composition**<br>• Most abundant genera *Moraxella* (33%), *Streptococcus* (18%), *Corynebacterium* (11%), *Haemophilus* (10%), and *Dolosigranulum* (4.6%)<br><br>**RSV infection**<br>• Lower richness and diversity at OTU level<br>• 19 bacterial genera differently abundant between infants with human rhinovirus and RSV infection	• 0% preterm born<br>• 64% vaginally born<br>• 75% breastfed<br>• 12% prior AB exposure<br>• 22% current AB exposure<br>• 21% maternal tobacco smoking<br>• 61% White non-Hispanic, 19% Black non-Hispanic, 9% Hispanic, 12% other

			2x300, median 20,000, range 5,302-80,070 reads/sample<br>SILVA 138		• -<br><br>• Storage condition nr<br>• Overlap with participants from(1, 3, 4)
Rosas-Salazar *et al.*(3)<br><br>USA<br><br>2016	Single-centre, prospective cohort study (2b)	132, 132<br>nr	Healthy (33, 33, median 22w, IQR 13-27w), RSV infection (99, 99, median 5w, IQR 2-9w)<br><br>Healthy infants: Filter paper Leucosorb B (*Pall Life Sciences*), sterile container<br>Infants with RSV infection: Nasal wash, nr<br>-20°C immediately, -80°C within 24h<br><br>Phenol:chloroform:isoamyl alcohol extractions<br>27F, 534R<br>16S rRNA, V1-V3, GS FLX Titanium (*454 Life Sciences/Roche)*<br>nr, mean >2,000 reads/sample, range 424-20,987<br>SILVA 138	**Composition**<br>• Most abundant genera in healthy infants *Streptococcus, Corynebacterium, Staphylococcus,* and *Dolosigranulum*<br><br>**RSV infection**<br>• Lower richness<br>• Most abundant genera *Streptococcus, Moraxella, Corynebacterium,* and *Haemophilus*<br>• Lower abundance of *Staphylococcus, Corynebacterium* and higher abundance of *Haemophilus, Moraxella,* and *Streptococcus* compared with healthy infants	• -<br><br>• Inclusion of healthy controls<br><br>• Samples collected with different methods in infants with RSV infection and healthy infants<br>• Storage medium nr<br>• Sequencing length nr<br>• No information on delivery mode, gestational age, feeding method, vaccination status, AB exposure, siblings, day-care attendance, pets, tobacco smoke exposure<br>• Overlap with participants from(1, 2, 4)
Shilts *et al.*(67)<br><br>USA<br><br>2016	Single-centre, prospective cohort study (2b)	33, 33<br>52	Healthy (33, 33)<br>Mean 51d, range 5d-4.5m<br><br>Filter paper Leucosorb B (*Pall Life Sciences*), sterile container<br>-20°C immediately, -80°C within 24h<br><br>Phenol:chloroform:isoamyl alcohol extractions<br>27F, 534R<br>16S rRNA, V1-V3, GS FLX Titanium (*454 Life Sciences/Roche)*<br>nr, mean 2x387, mean 14,471, range 2,617-21,098<br>SILVA 138	**Composition**<br>• Mean 62 OTUs/sample, range 21-135<br>• 328 genera<br>• Most abundant genera *Corynebacterium* (28%), *Streptococcus* (22%), *Staphylococcus* (20%), *Dolosigranulum* (6%), and *Moraxella* (4%)<br><br>**Delivery mode**<br>• Higher richness and diversity in infants born by CS<br>• Lower abundance of *Corynebacterium* and higher abundance of *Staphylococcus* in infants born by CS<br><br>**Feeding method**<br>• Higher richness in infants fed formula<br>• Higher abundance of *Moraxella* in infants fed formula<br><br>**No association between maternal AB during pregnancy, sex, pets, ethnicity, and composition**	• 0% preterm born<br>• 76% vaginally born<br>• 67% breastfed<br>• 6% prior AB exposure<br>• 58% pets<br>• 73% White, 24% Black<br><br>• -<br><br>• Small cohort<br>• Sequencing length nr<br>• No information on vaccination status, siblings, day-care attendance, tobacco smoke exposure
Zhou *et al.*(68)<br><br>USA<br><br>2016	Randomised, double-blinded, placebo-controlled trial (1b)	39, 74<br>59	RSV bronchiolitis with hospitalisation (39, 74), oral azithromycin or placebo for 14d<br>Mean 3.8m, SD 2.9m<br><br>Nasal wash, nr<br>nr<br><br>NucliSENS easyMAG extractor kit (*BioMerieux*)<br>16S rRNA, V1-V3, GS FLX Titanium (*454 Life Sciences/Roche)*<br>nr<br>>200, >1,000, median 6268 reads/sample<br>RDP v2.5	**Composition**<br>• Median 24-27 genera/sample<br>• Most abundant genera *Moraxella* and *Streptococcus*<br><br>**Antibiotics (azithromycin)**<br>• Decrease in abundance of *Moraxella* after 14d of azithromycin<br>• Placebo: Increase in abundance of *Dlosigranulum* and *Corynebacterium,* decrease in abundance of *Streptococcus*<br><br>**Wheezing**<br>• Lower abundance of *Moraxella* associated with lower rates of recurrent wheezing	• 8% maternal smoking during pregnancy<br>• 0% preterm born<br>• 28% breastfed<br>• 49% exposed to azithromycin<br>• 62% pets<br>• 36% exposure to tobacco smoke<br>• 64% White, 36% Black<br><br>• Randomisation and placebo-control<br><br>• Small cohort<br>• Storage medium and condition nr<br>• Low sequencing depth

					• No information on delivery mode, vaccination status, siblings, day-care attendance
Feazel *et al.*(69)<br><br>Kenya<br><br>2015	Multi-centre, retrospective cohort study (participants from a single-blinded, randomised controlled vaccine trial) (3b)	54, 108<br>44	Healthy (54, 108)<br>Mean 31m, SD 16m, range 12-59m<br>At vaccination and 6m after<br><br>Nasopharyngeal swab, rayon tipped swabs (*Medical Wire and Equipment Co*), sterile tubes with ethanol<br>-80°C time nr<br><br>UltraClean fecal DNA kit (*MoBio*)<br>27FYM+3, 534R<br>16S rRNA, V1-V3, GS FLX Titanium (*454 Life Sciences/Roche)*<br>>200, median 3,114 reads/sample<br>RDP and SILVA v104	**Composition**<br>• Median 22 genera/sample<br>• Most abundant genera *Moraxella, Streptococcus, Haemophilus,* and *Corynebacterium*<br>• Most abundant OTU *M. catarrhalis, M. nonliquefaciens, S. pneumoniae, H. influenzae* and *Corynebacterium*<br>• Positive association between abundance of *S. pneumoniae* and *H. influenzae*<br><br>**Age**<br>• Composition less stable in older children<br><br>**Vaccination**<br>• No influence on diversity or composition<br><br>**No association between sex, school attendance, AB exposure, and composition**	• 30% tobacco smoke exposure<br>• 50% vaccinated with 1 dose of Hib/PCV10<br>• Delivery mode, AB exposure or day-care attendance nr<br><br>• Analysis at species level*<br><br>• Small cohort<br>• Differences in vaccination status
Jervis-Bardy *et al.*(70)<br><br>Australia<br><br>2015	Single-centre prospective cohort study (participants from randomised controlled trial) (2b)	11, 11<br>55	Otitis media with effusion (11) randomised to receive either medical management, adenoidectomy and grommet insertion or adenoidectomy and myringotomy<br>Mean 5.3y, range 3-9y<br><br>Nasopharyngeal swab, FLOQSwab (*Copan*), STGG<br>On ice immediately, -80°C within 2h<br><br>*QIAamp* DNA Kit (*Qiagen)*<br>27F, 519R<br>16S rRNA, V1-V3, MiSeq (*Illumina*)<br>520 bp, >400 reads/sample, median 1,320 (range 436-35,935) (includes adenoid swabs and middle ear fluid)<br>SILVA 111	**Composition**<br>• 21 OTUs<br>• Most abundant OTUs *M. catarrhalis, H. influenzae, Streptococcus, Ornithobacterium, D. pigrum,* and *C. pseudodiphtheriticum*<br>• Overlap between microbiome in nasopharyngeal and adenoid swabs	• 100% vaccinated with PCV10 or PCV13<br>• 0% AB prior 4w<br>• Season of collection: 100% autumn/winter<br><br>• Small cohort<br>• Low sequencing depth<br>• Analysis at species level*<br><br>• Includes children across a wide age range<br>• No information on siblings, pets, tobacco smoke exposure
Stearns *et al.*(71)<br><br>Canada<br><br>2015	Multi-centre, prospective cohort study (2b)	51, 51<br>nr	Healthy (51, 51)<br>Median 13m, range 12-54m<br><br>Nasopharyngeal swab, ESwab (*Copan*)<br>Processed immediately or -20°C within 6-8h<br><br>Mechanical and enzymatic lysis (*MoBio*)<br>341F, 518R<br>16S rRNA, V3, MiSeq (*Illumina)*<br>nr, nr<br>GreenGenes 2011	**Age**<br>• Children have a lower diversity and higher evenness with a higher abundance of Proteobacteria, such as *Moraxella, Enterobacteriaceae* and *Haemophilus,* as well as a higher abundance of Firmicutes, such as *Enterococcus* compared with adults	• -<br><br>• -<br><br>• Small cohort<br>• Sequencing length and depth nr<br>• Delivery mode, feeding method, AB exposure, tobacco smoke exposure, siblings or day-care attendance nr
Teo *et al.*(63)	Multi-centre, prospective	234, 1,021<br>56	Healthy at risk for allergic sensitisation (234)<br>2, 6, 12m (487), during ARTI (URTI 154, LRTI 380)	**Composition**<br>• Most abundant genera: *Moraxella* (31%), *Streptococcus* (15.5%), *Corynebacterium* (13.5%), *Staphylococcus* (10.3%),	• 72% vaginally born<br>• 84% breastfed<br>• 0% vaccinated with PCV

Australia<br><br>2015	birth cohort study (2b)		Nasopharyngeal aspirate, nr<br>-80°C time nr<br><br>Wizard SV Genomic DNA System (*Promega*)<br>515F, 806R<br>16S rRNA, V4, MiSeq (I*llumina*)<br>2x151, mean > 200,000 reads/sample (IQR 108,000-255,000), eight samples <1,000 reads/sample<br>GreenGenes 13_5	*Haemophilus* (9.7%), and *Dolosigranulum* (8.8%; genus Dolosigranulum in some databases)<br>• Six profiles: *Moraxella*-dominated, *Streptococcus*-dominated, *Corynebacterium*-dominated, *Staphylococcus*-dominated, *Haemophilus*-dominated, and *Dolosigranulum*-dominated (also had a high abundance of *Corynebacterium)*<br><br>**Age**<br>• Higher abundance of *Staphylococcus* and *Corynebacterium* at 2m compared with 12m<br>• Lower abundance of *Dolosigranulum* and *Moraxella* at 2m compared with 12m<br><br>**ARTI**<br>• *Streptococcus-, Haemophilus-* and *Moraxella*-dominated profiles more frequent during ARTI<br>• *Dolosigranulum-, Staphylococcus-, Corynebacterium*-dominated profiles less during ARTI<br>• *Neisseria* was more commonly found in samples taken during ARTI<br>• Early colonisation with *Moraxella* associated with earlier URTI<br>• Early colonisation with *Streptococcus* associated with earlier LRTI<br>• Infants colonised with *Dolosigranulum* had fewer RSV infections, especially RSV LRTIs<br>• Frequent ARTIs associated with higher abundance of *Moraxella* and lower abundance of *Dolosigranulum* and *Corynebacterium*<br><br>**Antibiotics prior 4m**<br>• Higher abundances of *Haemophilus, Streptococcus,* and *Moraxella* and lower abundance of *Dolosigranulum* and *Corynebacterium*<br><br>**Day-care attendance**<br>• Higher abundances of *Haemophilus, Streptococcus,* and *Moraxella* and lower abundance of *Staphylococcus*<br><br>**Siblings**<br>• Higher abundances of *Haemophilus* and *Moraxella* and lower abundance of *Staphylococcus*<br><br>**Sex**<br>• Males higher abundance of *Moraxella* during healthy periods<br><br>**Furry pets**<br>• Lower abundance of *Streptococcus*<br><br>**Season**<br>• Higher number of *Haemophilus*-dominated profiles in spring/summer, higher number of *Moraxella*-dominated profiles in autumn-winter	• 47% siblings<br>• 29% day-care attendance<br>• 48% furry pet <1y<br>• 19% tobacco smoke exposure<br><br>• Longitudinal sample collection<br>• Large sample number<br><br>• Storage medium nr, antibiotic exposure nr<br>• Overlap with participants from(62)

				**Chronic wheezing**<br>• High abundance of *Streptococcus* before first ARTI associated with development of chronic wheezing<br><br>**No association between delivery mode and breastfeeding and composition**	
Biesbroek *et al.***(72)**<br><br>Netherlands<br><br>2014	Multi-centre, retrospective cohort study (participants from a single-blinded, randomised controlled vaccine trial)<br>(3b)	60, 240<br>nr	Healthy (60, 240)<br>1.5, 6, 12, 24m<br><br>Nasopharyngeal swab, Transwab Pernasal Plain (*Medical Wire and Equipment Co*), modified Amies Medium 483CE (Copan)<br>Room temperature <24h, -80°C<br><br>Phenol/bead-beating and magnetic bead separation<br>nr<br>16S rRNA, V5-V7, GS FLX Titanium (*454 Life Sciences/Roche)*<br>nr, >439/sample<br>RDP-II	**Composition**<br>• 10 phyla, 314 OTUs<br>• Most abundant genera *Moraxella, Streptococcus, Haemophilus, Dolosigranulum,* and *Corynebacterium*<br>• 8 profiles<br>• Negative correlation between *Staphylococcus* and *Streptococcus* and *Corynebacterium* and *Dolosigranulum* at 1.5m<br>• Composition more stable over time when dominated early on by *Moraxella*, *Dolosigranulum* and *Corynebacterium*, less stable when dominated by *Streptococcus, Haemophilus* and *Bacteroidetes*<br><br>**Age**<br>• No changes in diversity or density<br>• Higher abundance of *Corynebacterium, Dolosigranulum* and *Staphylococcus* and lower presence of *Haemophilus* at 1.5m<br>• Five different profiles at 1.5m: dominated by either *Streptococcus, Moraxella, Staphylococcus, Corynebacterium* or *Corynebacterium/Dolosigranulum*<br>• After 1.5m *Staphylococcus*-dominated profile disappeared, a *Haemophilus*-dominated profile emerged, *Corynebacterium/Dolosigranulum*-dominated profile was replaced by *Moraxella/Dolosigranulum*-dominated profile<br>• After 6m two *Moraxella*-dominated profiles, one with *M. catarrhalis* and one *M. lincolnii*<br>• Infants with a *Corynebacterium/Dolosigranulum*-dominated profile at 1.5m proceeded in almost all cases to *Moraxella*-dominated profile at 6m and older<br><br>**Breastfeeding**<br>• Composition more stable over time<br>• More frequently *Corynebacterium/Dolosigranulum*-dominated profile at 1.5m<br><br>**URTI**<br>• Infants with *Corynebacterium/Dolosigranulum*-dominated profile at 1.5 and 6 m and *Moraxella*-dominated profiles at 1.5, 6 and 12m had less frequent URTIs<br>• Composition more stable over time when less frequent URTIs<br><br>**PCV7**<br>• No influence on composition	• 93% vaginally born<br>• 62% exclusively breastfeed at 1.5m<br>• 50% exclusively breastfeed for 6m<br>• 63% ≥1 sibling<br>• 57% day-care attendance at 24m<br>• 7% tobacco smoke exposure<br>• 5% AB exposure in 4w prior to 12m sample<br>• 3% AB exposure in 4w prior to 24m sample<br>• 50% vaccinated ≥1 dose PCV7<br><br>• Longitudinal sample collection<br><br>• Small cohort<br>• Sequencing length nr<br>• Low sequencing depth<br>• Differences in vaccination status<br>• Parental-reported clinical outcomes<br>• Possible participants overlap with(73-75)

Biesbroek *et al.*(73)<br><br>Netherlands<br><br>2014	Multi-centre, retrospective cohort study (participants from a single-blinded, randomised controlled vaccine trial)<br>(3b)	202, 390<br>50	Healthy (202, 390)<br>1.5, 6m<br><br>Nasopharyngeal swab, Transwab Pernasal Plain (*Medical Wire and Equipment Co*), modified Amies Medium 483CE (Copan)<br>Room temperature <24h, -80°C<br><br>Phenol/bead-beating plus *AGOWA* Mag mini DNA extraction kit *(LGC Genomics)*<br>nr<br>16S rRNA, V5-V7, GS FLX Titanium (*454 Life Sciences/Roche)*<br>nr, mean 2,798 reads/sample<br>RDP-II, SILVA, and NCBI	**Composition**<br>• 13 phyla, 1,031 OTUs<br>• Positive association between abundance of *Dolosigranulum* and *Corynebacterium*<br>• Negative association between abundance of *Corynebacterium, Dolosigranulum* and *Moraxella, Staphylococcus*<br>• Negative association between abundance of *Corynebacterium, Dolosigranulum* and *Streptococcus* (including *S. pneumoniae*), *Veillonella, Rothia, Granulicatella, Actinomyces, Prevotella, Gemella, Leptotrichia, Fusobacterium,* and *Klebsiella*<br><br>**Age**<br>• Decrease in diversity and evenness with increasing age<br>• Decrease in colonisation rates by *S. aureus* and increase in colonisation rates by *H. influenzae, S. pneumoniae* and *M. catarrhalis*<br><br>**Breastfeeding**<br>• Higher evenness, no difference in diversity<br>• Higher abundance of *Corynebacterium* and *Dolosigranulum* at 1.5m (*D. pigrum*, *C. pseudodiphteriticum, C. propinquum, C. accolens, C. fastidiosum, C. segmentosum)* at 1.5m<br>• Lower abundance of *Staphylococcus, Actinomyces, Gemella, Veillonella, Prevotella, Rothia* and *Granulicatella* at 1.5m<br>• Lower abundance of *Granulicatella* at 6m<br><br>**URTI and wheezing**<br>• Higher abundance of *Dolosigranulum* associated with less frequent URTIs and wheezing<br>• Higher abundance of *Gemella* associated with more frequent URTIs	• 89% vaginally born<br>• 62% exclusively breastfeed at 1.5m<br>• 50% exclusively breastfeed at 6m<br>• 63% vaccinated ≥1 dose PCV7<br>• 5% AB exposure in 4w prior to 1.5m sample<br>• 4% AB exposure in 4w prior to 6m sample<br>• 70% ≥1 sibling<br>• 51% day-care attendance at 24m<br>• 11% tobacco smoke exposure<br><br>• Analysis at species level*<br><br>• Sequencing length nr<br>• Differences in vaccination status<br>• Possible participants overlap with(72, 74, 75)
Biesbroek *et al.*(75)<br><br>Netherlands<br><br>2014	Multi-centre, retrospective cohort study (participants from a single-blinded, randomised controlled vaccine trial)<br>(3b)	200, 200<br>54	Healthy (200, 200)<br>12, 24m<br><br>Nasopharyngeal swab, Transwab Pernasal Plain (*Medical Wire and Equipment Co*), modified Amies Medium 483CE (Copan)<br>Room temperature <24h, -80°C<br><br>Phenol/Bead-beating, magnetic bead separation<br>785F 1061R<br>16S rRNA, V5-V7, GS FLX Titanium (*454 Life Sciences/Roche)*<br>nr, mean 2,561, SD 767 reads/sample<br>RDP-II	**Composition**<br>• 10 phyla, 424 OTUs<br><br>**PCV7**<br>• Higher diversity<br>• Higher abundance of *Haemophilus, Staphylococcus, Veillonella, Prevotella, Bacteroidetes, Leptorichia,* and *Streptococcus* at 12m<br>• No difference at 24m	• 49% vaccinated ≥1 dose PCV7<br>• 65% siblings<br>• 75% day-care attendance at 24m<br>• 6% tobacco smoke exposure<br>• 5% AB exposure in 4w prior to 12m sample<br>• 2% AB exposure in 4w prior to 6m sample<br><br>• Analysis at species level*<br><br>• Sequencing length nr<br>• No information on gestational age, delivery mode, feeding method<br>• Overlap with participants from(72-74)
Sakwinska *et al.*(76)<br><br>Switzerland	Multi-centre prospective case-control study<br>(3b)	100, 100<br>59	Healthy (50, 50), pneumonia (50, 50)<br>Median 30m, range 2m-16y<br><br>Nasopharyngeal swab, nr (*Copan*), Amies medium (*Copan*)	**Composition**<br>• 250 OTUs<br><br>**Pneumonia**<br>• Lower bacteria richness and diversity	• Exclusion of children with chronic disease and RTI in past 21d<br><br>• Inclusion of healthy controls<br>• Analysis at species level*

2014			-80°C immediately<br><br>*In-house* DNA extraction methods<br>nr<br>16S rRNA, V1-V2, GS FLX Titanium (*454 Life Sciences/Roche)*<br>nr, nr<br>RDP v2.3	• Higher abundance of *Moraxella, Haemophilus, Streptococcus* during non-viral pneumonia<br>• Higher abundance of *M. lacunata* during viral pneumonia	• Includes children across a wide age range<br>• Swab nr<br>• Sequencing length and depth nr
Hilty *et al.*(77)<br><br>Switzerland<br><br>2012	Multi-centre, prospective cohort study (2b)	163, 163<br>51	Healthy (10, 10), AOM (153, 153)<br>Range 0-24m<br><br>Nasopharyngeal swab, Rayon tipped swabs (*Copan*), nr<br>nr<br><br>*QIAamp* DNA mini kit (*Qiagen)*<br>341F, 926R<br>16S rRNA, V3-V5, GS FLX Titanium (*454 Life Sciences/Roche)*<br>nr, nr<br>nr	**Composition**<br>• 58 bacterial families<br>• Most abundant families *Moraxellaceae, Streptococcaceae,* and *Pasteurellaceae*<br><br>**AOM**<br>• Lower richness and diversity, higher density<br>• Lower abundance of *Staphylococcaceae, Comamonadaceae*, *Acidaminococcaceae, Corynebacteriaceae*<br><br>**Antibiotics prior 2m**<br>• Lower abundance of Moraxellaceae, *Streptococcaceae,* and *Staphylococcaceae*<br>• Higher abundance *Pasteurellaceae*<br><br>**PCV7**<br>• Less children colonised with *Streptococcaceae* and *Corynebacteriacea*<br><br>**No association between age, sex, day-care attendance, recurrent AOM and composition**	• 25% AB exposure prior 8w<br>• 33% day-care attendance<br>• 58% vaccinated with ≥ 1 PCV7<br><br>• Inclusion of healthy controls<br><br>• Small control group, some control children were HIV or Hepatitis C exposed or had suffered from repeated skin abscesses<br>• Storage medium and condition nr<br>• Sequencing length and depth nr<br>• Database used for taxonomic identification nr<br>• Results only reported on family levels<br>• Delivery mode, feeding method, tobacco smoke exposure or siblings nr
Bogaert *et al.*(74)<br><br>Netherlands<br><br>2011	Multi-centre, retrospective cohort study (participants from a single-blinded, randomised controlled vaccine trial) (3b)	96, 96<br>49	Healthy (52, 52), AOM (4, 4), URTI (40, 40)<br>18m<br><br>Nasopharyngeal swab, Transwab Pernasal Plain (*Medical Wire and Equipment Co*), modified Amies Medium 483CE (Copan)<br>Room temperature < 24h, -80°C<br><br>Phenol/Bead-beating, magnetic bead separation<br>785F 1061R<br>16S rRNA, V5-V6, GS FLX Titanium (*454 Life Sciences/Roche)*<br>nr, mean 11,000 reads/sample<br>RDP-II and SILVA	**Composition**<br>• 13 phyla, 243 OTUs (mean 40 OTUs/sample)<br>• Most abundant phyla Proteobacteria (64%), Firmicutes (21%), Bacteroidetes (11%), Actinobacteria (3%), and Fusobacteria (1%)<br>• Most abundant genera *Moraxella* (40%)*, Haemophilus* (20%), *Streptococcus* (12%), *Flavobacteria* (10%)*, Dolosigranulum* (5%), *Corynebacterium* (2%)*, Neisseria* (2%), and Fusobacterium (1%)<br>• 3 profiles: *Moraxella*-, *H. influenzae*-, and *Streptococcus*-dominated<br><br>**Season**<br>• Higher abundance of Proteobacteria, Fusobacteria, and Cyanobacteria in autumn/winter<br>• Higher abundance of Bacteroides in spring<br>• Higher abundance of *Bacillus, Brevibacillus, Flavobacterium* and *Lactobacillus* in spring<br>• Higher abundance of *B. fragilis* in spring	• 0% PCV vaccinated<br>• 21% prior AB exposure<br>• 58% siblings<br>• 67% day-care attendance<br>• 8% tobacco smoke exposure<br><br>• Analysis at species level*<br><br>• Includes children with corticosteroid treatment<br>• Sequencing length nr<br>• Overlap with participants from(72, 73, 75)

					**No association between AB exposure, day-care attendance, tobacco smoke exposure, presence of respiratory viruses and composition**	

[*] or OTUs on species level
[**] measured my maternal alpha1,2-fucosyltransferase 2 and alpha 1.3/4-fucosyltransferase 3 polymorphism

AB – antibiotics
AOM – acute otitis media
ARTI – acute respiratory tract infection
ASV - amplicon sequence variant
CCL5 - chemokine ligand 5
d – days
h – hours
HIV – human immunodeficiency virus
ICU – intensive care unit
IQR – interquartile range
ITS – internal transcribed spacer
m – months
nr – not reported
LRTI – lower respiratory tract infection
NCBI – National Center for Biotechnology Information
OTU – operational taxonomic unit
PCV – pneumococcus conjugated vaccine
RDP – ribosomal database project
RSV – respiratory syncytial virus
SD – standard deviation
STGG – skim milk, tryptone, glucose, glycerol
URTI – upper respiratory tract infection
w – weeks
y – years

1. Tan Y, Shilts MH, Rosas-Salazar C, Puri V, Fedorova N, Halpin RA, et al. Influence of Sex on Respiratory Syncytial Virus Genotype Infection Frequency and Nasopharyngeal Microbiome. Journal of virology. 2023;97(3):e0147222.
2. Rosas-Salazar C, Shilts MH, Tovchigrechko A, Schobel S, Chappell JD, Larkin EK, et al. Differences in the Nasopharyngeal Microbiome During Acute Respiratory Tract Infection With Human Rhinovirus and Respiratory Syncytial Virus in Infancy. The Journal of infectious diseases. 2016;214(12):1924-8.
3. Rosas-Salazar C, Shilts MH, Tovchigrechko A, Chappell JD, Larkin EK, Nelson KE, et al. Nasopharyngeal Microbiome in Respiratory Syncytial Virus Resembles Profile Associated with Increased Childhood Asthma Risk. American journal of respiratory and critical care medicine. 2016;193(10):1180-3.
4. Rosas-Salazar C, Shilts MH, Tovchigrechko A, Schobel S, Chappell JD, Larkin EK, et al. Nasopharyngeal Lactobacillus is associated with a reduced risk of childhood wheezing illnesses following acute respiratory syncytial virus infection in infancy. The Journal of allergy and clinical immunology. 2018;142(5):1447-56.e9.
5. Hou J, Song Y, Leung ASY, Tang MF, Shi M, Wang EY, et al. Temporal Dynamics of the Nasopharyngeal Microbiome and its Relationship with Childhood Asthma Exacerbation. Microbiol Spectr. 2022;10(3):e0012922.
6. Kelly MS, Plunkett C, Yu Y, Aquino JN, Patel SM, Hurst JH, et al. Non-diphtheriae Corynebacterium species are associated with decreased risk of pneumococcal colonization during infancy. The ISME journal. 2022;16(3):655-65.
7. McCauley KE, Flynn K, Calatroni A, DiMassa V, LaMere B, Fadrosh DW, et al. Seasonal airway microbiome and transcriptome interactions promote childhood asthma exacerbations. The Journal of allergy and clinical immunology. 2022.
8. Aydin M, Weisser C, Rué O, Mariadassou M, Maaß S, Behrendt AK, et al. The Rhinobiome of Exacerbated Wheezers and Asthmatics: Insights From a German Pediatric Exacerbation Network. Front Allergy. 2021;2:667562.
9. Binia A, Siegwald L, Sultana S, Shevlyakova M, Lefebvre G, Foata F, et al. The Influence of FUT2 and FUT3 Polymorphisms and Nasopharyngeal Microbiome on Respiratory Infections in Breastfed Bangladeshi Infants from the Microbiota and Health Study. mSphere. 2021;6(6):e0068621.
10. Coleman A, Zaugg J, Wood A, Cottrell K, Håkansson EG, Adams J, et al. Upper Respiratory Tract Microbiome of Australian Aboriginal and Torres Strait Islander Children in Ear and Nose Health and Disease. Microbiol Spectr. 2021;9(2):e0036721.
11. Chun Y, Do A, Grishina G, Arditi Z, Ribeiro V, Grishin A, et al. The nasal microbiome, nasal transcriptome, and pet sensitization. The Journal of allergy and clinical immunology. 2021;148(1):244-9.e4.
12. Elling CL, Scholes MA, Streubel SO, Larson ED, Wine TM, Bootpetch TC, et al. The FUT2 Variant c.461G>A (p.Trp154*) Is Associated With Differentially Expressed Genes and Nasopharyngeal Microbiota Shifts in Patients With Otitis Media. Front Cell Infect Microbiol. 2021;11:798246.
13. Folino F, Fattizzo M, Ruggiero L, Oriano M, Aliberti S, Blasi F, et al. Nasopharyngeal Microbiota Analysis in Healthy and Otitis-prone Children: Focus on History of Spontaneous Tympanic Membrane Perforation. Pediatr Infect Dis J. 2021;40(1):16-21.
14. Henares D, Brotons P, de Sevilla MF, Fernandez-Lopez A, Hernandez-Bou S, Perez-Argüello A, et al. Differential nasopharyngeal microbiota composition in children according to respiratory health status. Microb Genom. 2021;7(10).
15. McCauley KE, DeMuri G, Lynch K, Fadrosh DW, Santee C, Nagalingam NN, et al. Moraxella-dominated pediatric nasopharyngeal microbiota associate with upper respiratory infection and sinusitis. PloS one. 2021;16(12):e0261179.
16. Raita Y, Pérez-Losada M, Freishtat RJ, Hahn A, Castro-Nallar E, Ramos-Tapia I, et al. Nasopharyngeal metatranscriptome profiles of infants with bronchiolitis and risk of childhood asthma: a multicentre prospective study. The European respiratory journal. 2021.

17. Hasegawa K, Mansbach JM, Ajami NJ, Espinola JA, Henke DM, Petrosino JF, et al. Association of nasopharyngeal microbiota profiles with bronchiolitis severity in infants hospitalised for bronchiolitis. The European respiratory journal. 2016;48(5):1329-39.
18. Fujiogi M, Camargo CA, Jr., Raita Y, Toivonen L, Freishtat RJ, Mansbach JM, et al. Association of endemic coronaviruses with nasopharyngeal metabolome and microbiota among infants with severe bronchiolitis: a prospective multicenter study. Pediatric research. 2021;89(7):1594-7.
19. Mansbach JM, Hasegawa K, Piedra PA, Avadhanula V, Petrosino JF, Sullivan AF, et al. Haemophilus-Dominant Nasopharyngeal Microbiota Is Associated With Delayed Clearance of Respiratory Syncytial Virus in Infants Hospitalized for Bronchiolitis. The Journal of infectious diseases. 2019;219(11):1804-8.
20. Mansbach JM, Luna PN, Shaw CA, Hasegawa K, Petrosino JF, Piedra PA, et al. Increased Moraxella and Streptococcus species abundance after severe bronchiolitis is associated with recurrent wheezing. The Journal of allergy and clinical immunology. 2020;145(2):518-27.e8.
21. Luna PN, Hasegawa K, Ajami NJ, Espinola JA, Henke DM, Petrosino JF, et al. The association between anterior nares and nasopharyngeal microbiota in infants hospitalized for bronchiolitis. Microbiome. 2018;6(1):2.
22. Toivonen L, Camargo CA, Jr., Gern JE, Bochkov YA, Mansbach JM, Piedra PA, Hasegawa K. Association between rhinovirus species and nasopharyngeal microbiota in infants with severe bronchiolitis. The Journal of allergy and clinical immunology. 2019;143(5):1925-8.e7.
23. Toivonen L, Hasegawa K, Ajami NJ, Celedón JC, Mansbach JM, Petrosino JF, Camargo CA, Jr. Circulating 25-hydroxyvitamin D, nasopharyngeal microbiota, and bronchiolitis severity. Pediatr Allergy Immunol. 2018;29(8):877-80.
24. Hasegawa K, Mansbach JM, Ajami NJ, Petrosino JF, Freishtat RJ, Teach SJ, et al. The relationship between nasopharyngeal CCL5 and microbiota on disease severity among infants with bronchiolitis. Allergy. 2017;72(11):1796-800.
25. Stewart CJ, Mansbach JM, Ajami NJ, Petrosino JF, Zhu Z, Liang L, et al. Serum Metabolome Is Associated With the Nasopharyngeal Microbiota and Disease Severity Among Infants With Bronchiolitis. The Journal of infectious diseases. 2019;219(12):2005-14.
26. Stewart CJ, Mansbach JM, Wong MC, Ajami NJ, Petrosino JF, Camargo CA, Jr., Hasegawa K. Associations of Nasopharyngeal Metabolome and Microbiome with Severity among Infants with Bronchiolitis. A Multiomic Analysis. American journal of respiratory and critical care medicine. 2017;196(7):882-91.
27. Reyman M, Clerc M, van Houten MA, Arp K, Chu M, Hasrat R, et al. Microbial community networks across body sites are associated with susceptibility to respiratory infections in infants. Commun Biol. 2021;4(1):1233.
28. Bosch A, Levin E, van Houten MA, Hasrat R, Kalkman G, Biesbroek G, et al. Development of Upper Respiratory Tract Microbiota in Infancy is Affected by Mode of Delivery. EBioMedicine. 2016;9:336-45.
29. Bosch A, de Steenhuijsen Piters WAA, van Houten MA, Chu M, Biesbroek G, Kool J, et al. Maturation of the Infant Respiratory Microbiota, Environmental Drivers, and Health Consequences. A Prospective Cohort Study. American journal of respiratory and critical care medicine. 2017;196(12):1582-90.
30. Man WH, Clerc M, de Steenhuijsen Piters WAA, van Houten MA, Chu M, Kool J, et al. Loss of Microbial Topography between Oral and Nasopharyngeal Microbiota and Development of Respiratory Infections Early in Life. American journal of respiratory and critical care medicine. 2019;200(6):760-70.
31. Tang HHF, Lang A, Teo SM, Judd LM, Gangnon R, Evans MD, et al. Developmental patterns in the nasopharyngeal microbiome during infancy are associated with asthma risk. The Journal of allergy and clinical immunology. 2021;147(5):1683-91.
32. Tozzi AE, Del Chierico F, Pandolfi E, Reddel S, Gesualdo F, Gardini S, et al. Nasopharyngeal microbiota in hospitalized children with Bordetella pertussis and Rhinovirus infection. Scientific reports. 2021;11(1):22858.

33. Xu L, Earl J, Pichichero ME. Nasopharyngeal microbiome composition associated with Streptococcus pneumoniae colonization suggests a protective role of Corynebacterium in young children. PloS one. 2021;16(9):e0257207.
34. Xu L, Earl J, Bajorski P, Gonzalez E, Pichichero ME. Nasopharyngeal microbiome analyses in otitis-prone and otitis-free children. International journal of pediatric otorhinolaryngology. 2021;143:110629.
35. Accorsi EK, Franzosa EA, Hsu T, Joice Cordy R, Maayan-Metzger A, Jaber H, et al. Determinants of Staphylococcus aureus carriage in the developing infant nasal microbiome. Genome biology. 2020;21(1):301.
36. Chapman TJ, Morris MC, Xu L, Pichichero ME. Nasopharyngeal colonization with pathobionts is associated with susceptibility to respiratory illnesses in young children. PloS one. 2020;15(12):e0243942.
37. Enoksson F, Ruiz Rodriguez A, Peno C, Balcazar Lopez C, Tjernström F, Bogaert D, et al. Niche- and Gender-Dependent Immune Reactions in Relation to the Microbiota Profile in Pediatric Patients with Otitis Media with Effusion. Infection and immunity. 2020;88(10).
38. Haro K, Ogawa M, Saito M, Kusuhara K, Fukuda K. Bacterial composition of nasal discharge in children based on highly accurate 16S rRNA gene sequencing analysis. Scientific reports. 2020;10(1):20193.
39. Liu Q, Liu Q, Meng H, Lv H, Liu Y, Liu J, et al. Staphylococcus epidermidis Contributes to Healthy Maturation of the Nasal Microbiome by Stimulating Antimicrobial Peptide Production. Cell host & microbe. 2020;27(1):68-78.e5.
40. Man WH, Scheltema NM, Clerc M, van Houten MA, Nibbelke EE, Achten NB, et al. Infant respiratory syncytial virus prophylaxis and nasopharyngeal microbiota until 6 years of life: a subanalysis of the MAKI randomised controlled trial. The Lancet Respiratory medicine. 2020;8(10):1022-31.
41. Salgado VR, Fukutani KF, Fukutani E, Lima JV, Rossi EA, Barral A, et al. Effects of 10-valent pneumococcal conjugate (PCV10) vaccination on the nasopharyngeal microbiome. Vaccine. 2020;38(6):1436-43.
42. Shilts MH, Rosas-Salazar C, Lynch CE, Tovchigrechko A, Boone HH, Russell PB, et al. Evaluation of the upper airway microbiome and immune response with nasal epithelial lining fluid absorption and nasal washes. Scientific reports. 2020;10(1):20618.
43. Thapa S, Runge JK, Venkatachalam A, Denne C, Luna RA, Anon JB. The Nasopharyngeal and Gut Microbiota in Children in a Pediatric Otolaryngology Practice. Pediatr Infect Dis J. 2020;39(9):e226-e33.
44. Verhagen LM, Rivera-Olivero IA, Clerc M, Chu M, van Engelsdorp Gastelaars J, Kristensen MI, et al. Nasopharyngeal Microbiota Profiles in Rural Venezuelan Children Are Associated With Respiratory and Gastrointestinal Infections. Clin Infect Dis. 2021;72(2):212-21.
45. Zhou Q, Xie G, Liu Y, Wang H, Yang Y, Shen K, et al. Different nasopharynx and oropharynx microbiota imbalance in children with Mycoplasma pneumoniae or influenza virus infection. Microbial pathogenesis. 2020;144:104189.
46. Lu Z, Dai W, Liu Y, Zhou Q, Wang H, Li D, et al. The Alteration of Nasopharyngeal and Oropharyngeal Microbiota in Children with MPP and Non-MPP. Genes (Basel). 2017;8(12).
47. Wen Z, Xie G, Zhou Q, Qiu C, Li J, Hu Q, et al. Distinct Nasopharyngeal and Oropharyngeal Microbiota of Children with Influenza A Virus Compared with Healthy Children. Biomed Res Int. 2018;2018:6362716.
48. Dai W, Wang H, Zhou Q, Feng X, Lu Z, Li D, et al. The concordance between upper and lower respiratory microbiota in children with Mycoplasma pneumoniae pneumonia. Emerg Microbes Infect. 2018;7(1):92.
49. Boelsen LK, Dunne EM, Mika M, Eggers S, Nguyen CD, Ratu FT, et al. The association between pneumococcal vaccination, ethnicity, and the nasopharyngeal microbiota of children in Fiji. Microbiome. 2019;7(1):106.
50. Man WH, van Houten MA, Mérelle ME, Vlieger AM, Chu M, Jansen NJG, et al. Bacterial and viral respiratory tract microbiota and host characteristics in children with lower respiratory tract infections: a matched case-control study. The Lancet Respiratory medicine. 2019;7(5):417-26.

51. Man WH, van Dongen TMA, Venekamp RP, Pluimakers VG, Chu M, van Houten MA, et al. Respiratory Microbiota Predicts Clinical Disease Course of Acute Otorrhea in Children With Tympanostomy Tubes. Pediatr Infect Dis J. 2019;38(6):e116-e25.
52. McCauley K, Durack J, Valladares R, Fadrosh DW, Lin DL, Calatroni A, et al. Distinct nasal airway bacterial microbiotas differentially relate to exacerbation in pediatric patients with asthma. The Journal of allergy and clinical immunology. 2019;144(5):1187-97.
53. Walker RE, Walker CG, Camargo CA, Jr., Bartley J, Flint D, Thompson JMD, Mitchell EA. Nasal microbial composition and chronic otitis media with effusion: A case-control study. PloS one. 2019;14(2):e0212473.
54. Yau JW, Hou J, Tsui SKW, Leung TF, Cheng NS, Yam JC, et al. Characterization of ocular and nasopharyngeal microbiome in allergic rhinoconjunctivitis. Pediatr Allergy Immunol. 2019;30(6):624-31.
55. Ederveen THA, Ferwerda G, Ahout IM, Vissers M, de Groot R, Boekhorst J, et al. Haemophilus is overrepresented in the nasopharynx of infants hospitalized with RSV infection and associated with increased viral load and enhanced mucosal CXCL8 responses. Microbiome. 2018;6(1):10.
56. Kelly MS, Surette MG, Smieja M, Rossi L, Luinstra K, Steenhoff AP, et al. Pneumococcal Colonization and the Nasopharyngeal Microbiota of Children in Botswana. Pediatr Infect Dis J. 2018;37(11):1176-83.
57. Kelly MS, Surette MG, Smieja M, Pernica JM, Rossi L, Luinstra K, et al. The Nasopharyngeal Microbiota of Children With Respiratory Infections in Botswana. Pediatr Infect Dis J. 2017;36(9):e211-e8.
58. Lappan R, Imbrogno K, Sikazwe C, Anderson D, Mok D, Coates H, et al. A microbiome case-control study of recurrent acute otitis media identified potentially protective bacterial genera. BMC microbiology. 2018;18(1):13.
59. Pérez-Losada M, Authelet KJ, Hoptay CE, Kwak C, Crandall KA, Freishtat RJ. Pediatric asthma comprises different phenotypic clusters with unique nasal microbiotas. Microbiome. 2018;6(1):179.
60. Pérez-Losada M, Alamri L, Crandall KA, Freishtat RJ. Nasopharyngeal Microbiome Diversity Changes over Time in Children with Asthma. PloS one. 2017;12(1):e0170543.
61. Pérez-Losada M, Crandall KA, Freishtat RJ. Two sampling methods yield distinct microbial signatures in the nasopharynges of asthmatic children. Microbiome. 2016;4(1):25.
62. Teo SM, Tang HHF, Mok D, Judd LM, Watts SC, Pham K, et al. Airway Microbiota Dynamics Uncover a Critical Window for Interplay of Pathogenic Bacteria and Allergy in Childhood Respiratory Disease. Cell host & microbe. 2018;24(3):341-52.e5.
63. Teo SM, Mok D, Pham K, Kusel M, Serralha M, Troy N, et al. The infant nasopharyngeal microbiome impacts severity of lower respiratory infection and risk of asthma development. Cell host & microbe. 2015;17(5):704-15.
64. Chonmaitree T, Jennings K, Golovko G, Khanipov K, Pimenova M, Patel JA, et al. Nasopharyngeal microbiota in infants and changes during viral upper respiratory tract infection and acute otitis media. PloS one. 2017;12(7):e0180630.
65. Perez GF, Pérez-Losada M, Isaza N, Rose MC, Colberg-Poley AM, Nino G. Nasopharyngeal microbiome in premature infants and stability during rhinovirus infection. J Investig Med. 2017;65(6):984-90.
66. Salter SJ, Turner C, Watthanaworawit W, de Goffau MC, Wagner J, Parkhill J, et al. A longitudinal study of the infant nasopharyngeal microbiota: The effects of age, illness and antibiotic use in a cohort of South East Asian children. PLoS neglected tropical diseases. 2017;11(10):e0005975.
67. Shilts MH, Rosas-Salazar C, Tovchigrechko A, Larkin EK, Torralba M, Akopov A, et al. Minimally Invasive Sampling Method Identifies Differences in Taxonomic Richness of Nasal Microbiomes in Young Infants Associated with Mode of Delivery. Microb Ecol. 2016;71(1):233-42.
68. Zhou Y, Bacharier LB, Isaacson-Schmid M, Baty J, Schechtman KB, Sajol G, et al. Azithromycin therapy during respiratory syncytial virus bronchiolitis: Upper airway microbiome alterations and subsequent recurrent wheeze. The Journal of allergy and clinical immunology. 2016;138(4):1215-9.e5.
69. Feazel LM, Santorico SA, Robertson CE, Bashraheil M, Scott JA, Frank DN, Hammitt LL. Effects of Vaccination with 10-Valent Pneumococcal Non-Typeable Haemophilus influenza Protein D Conjugate

Vaccine (PHiD-CV) on the Nasopharyngeal Microbiome of Kenyan Toddlers. PloS one. 2015;10(6):e0128064.
70. Jervis-Bardy J, Rogers GB, Morris PS, Smith-Vaughan HC, Nosworthy E, Leong LE, et al. The microbiome of otitis media with effusion in Indigenous Australian children. International journal of pediatric otorhinolaryngology. 2015;79(9):1548-55.
71. Stearns JC, Davidson CJ, McKeon S, Whelan FJ, Fontes ME, Schryvers AB, et al. Culture and molecular-based profiles show shifts in bacterial communities of the upper respiratory tract that occur with age. The ISME journal. 2015;9(5):1268.
72. Biesbroek G, Tsivtsivadze E, Sanders EA, Montijn R, Veenhoven RH, Keijser BJ, Bogaert D. Early respiratory microbiota composition determines bacterial succession patterns and respiratory health in children. American journal of respiratory and critical care medicine. 2014;190(11):1283-92.
73. Biesbroek G, Bosch AA, Wang X, Keijser BJ, Veenhoven RH, Sanders EA, Bogaert D. The impact of breastfeeding on nasopharyngeal microbial communities in infants. American journal of respiratory and critical care medicine. 2014;190(3):298-308.
74. Bogaert D, Keijser B, Huse S, Rossen J, Veenhoven R, van Gils E, et al. Variability and diversity of nasopharyngeal microbiota in children: a metagenomic analysis. PloS one. 2011;6(2):e17035.
75. Biesbroek G, Wang X, Keijser BJ, Eijkemans RM, Trzciński K, Rots NY, et al. Seven-valent pneumococcal conjugate vaccine and nasopharyngeal microbiota in healthy children. Emerging infectious diseases. 2014;20(2):201-10.
76. Sakwinska O, Bastic Schmid V, Berger B, Bruttin A, Keitel K, Lepage M, et al. Nasopharyngeal microbiota in healthy children and pneumonia patients. Journal of clinical microbiology. 2014;52(5):1590-4.
77. Hilty M, Qi W, Brugger SD, Frei L, Agyeman P, Frey PM, et al. Nasopharyngeal microbiota in infants with acute otitis media. The Journal of infectious diseases. 2012;205(7):1048-55.
